# Antiproliferative Activity Predictor: A New Reliable In Silico Tool for Drug Response Prediction against NCI60 Panel

**DOI:** 10.3390/ijms232214374

**Published:** 2022-11-19

**Authors:** Annamaria Martorana, Gabriele La Monica, Alessia Bono, Salvatore Mannino, Silvestre Buscemi, Antonio Palumbo Piccionello, Carla Gentile, Antonino Lauria, Daniele Peri

**Affiliations:** 1 Dipartimento di Scienze e Tecnologie Biologiche Chimiche e Farmaceutiche “STEBICEF”, Università Degli Studi di Palermo, Viale Delle Scienze Ed. 17, I-90128 Palermo, Italy; 2 Dipartimento di Ingegneria Dell’innovazione Industriale e Digitale, Università Degli Studi di Palermo, Viale Delle Scienze Ed. 6, I-90128 Palermo, Italy

**Keywords:** antiproliferative activity predictor, NCI60, DRUDIT, ligand-based tools, molecular descriptors, GI_50_

## Abstract

In vitro antiproliferative assays still represent one of the most important tools in the anticancer drug discovery field, especially to gain insights into the mechanisms of action of anticancer small molecules. The NCI-DTP (National Cancer Institute Developmental Therapeutics Program) undoubtedly represents the most famous project aimed at rapidly testing thousands of compounds against multiple tumor cell lines (NCI60). The large amount of biological data stored in the National Cancer Institute (NCI) database and many other databases has led researchers in the fields of computational biology and medicinal chemistry to develop tools to predict the anticancer properties of new agents in advance. In this work, based on the available antiproliferative data collected by the NCI and the manipulation of molecular descriptors, we propose the new in silico Antiproliferative Activity Predictor (AAP) tool to calculate the GI_50_ values of input structures against the NCI60 panel. This ligand-based protocol, validated by both internal and external sets of structures, has proven to be highly reliable and robust. The obtained GI_50_ values of a test set of 99 structures present an error of less than ±1 unit. The AAP is more powerful for GI_50_ calculation in the range of 4–6, showing that the results strictly correlate with the experimental data. The encouraging results were further supported by the examination of an in-house database of curcumin analogues that have already been studied as antiproliferative agents. The AAP tool identified several potentially active compounds, and a subsequent evaluation of a set of molecules selected by the NCI for the one-dose/five-dose antiproliferative assays confirmed the great potential of our protocol for the development of new anticancer small molecules. The integration of the AAP tool in the free web service DRUDIT provides an interesting device for the discovery and/or optimization of anticancer drugs to the medicinal chemistry community. The training set will be updated with new NCI-tested compounds to cover more chemical spaces, activities, and cell lines. Currently, the same protocol is being developed for predicting the TGI (total growth inhibition) and LC_50_ (median lethal concentration) parameters to estimate toxicity profiles of small molecules.

## 1. Introduction

In the search for new chemical compounds endowed with anticancer properties, in vitro antiproliferative screening remains one of the most commonly used approaches to identify new biologically active compounds. As demonstrated by the numerous research projects focused on the characterization of tumor cells (including National Cancer Institute Human Tumor Cell Lines Screen, NCI60 [1]; Cancer Cell Line Encyclopedia, CCLE [2]; Genomics of Drug Sensitivity in Cancer, GDSC [3]; The Cancer Genome Atlas Program, TCGA [4]; Cancer Therapeutics Response Portal, CTRP [5]), the study of cancer cell lines has led to gaining insights into the metabolic pathways involved in uncontrolled growth and the mechanisms of action of the tested anticancer compounds [6,7,8].

The NCI60 Human Tumor Cell Lines Screen is the pioneering and best-known project based on the large-scale screening of chemical compounds and cancer cell phenotypes. For more than 20 years, it has been of great benefit to the global research community and has proven to be a rich and reliable source of information. The NCI60 high-throughput screening program, in its current version, allows the screening of up to 3000 compounds per year and consists of a standardized assay performed on approximately sixty cancer cell lines belonging to nine different subpanels (each one related to a specific tumor: leukemia, non-small-cell lung (NSCLC), colon, central nervous system (CNS), melanoma, ovarian, renal, prostate, and breast cancer cells) characterized at the genomic, transcriptomic, and proteomic levels (Molecular Target Characterization Program) [1,9,10,11,12,13].

Considering the high failure rate in anticancer drug discovery research and the significant amount of time and resources required for in vitro screening, in silico predictions, especially in the early stages of the drug development, could represent a critical aid. To this end, several computational protocols and machine learning approaches have been proposed, as recently reviewed by Firoozbakht et al. [14].

In particular, COMPARE Analysis was the first tool that used the NCI database to correlate biological data with chemical entries (compound–compound; compound–target; target–target) permitting researchers to hypothesize the mechanism of action/resistance of a compound and the putative targets involved in its antiproliferative activity [15,16]. The great potential of this tool encouraged the development of other high-performing protocols, such as CellMiner, a web-accessible correlation application exploiting the NCI database [17,18,19]. CellMiner tools enable the rapid data retrieval of transcripts for genes and microRNAs, along with activity reports for thousands of chemical compounds, including several drugs approved by the Food and Drug Administration (FDA).

The elaboration of these data into quantitative patterns against the NCI60 dataset clarified their cross-correlational organization using a novel pattern matching tool. To this end, several case studies of the in silico discovery process afforded by CellMiner have been reported in the literature (i.e., analyses of multidrug resistance and doxorubicin activity; the identification of colon-specific genes, microRNAs, and drugs; etc.). 

Recently, Lind et al. developed a regression random forest model by the integration of mutational status data of 145 selected oncogenes from the GDSC database and more than 1200 molecular descriptors to reliably calculate the GI_50_ values of selected compounds against cancer cells [20]. Similarly, Zhang et al. designed a dual-layer integrated cell–drug network capable of predicting drug responses using the gene expression profiles of numerous cancer cell types available in the CCLE and the comprehensive genomic profiling (CGP), providing the chemical proprieties of drugs captured by molecular descriptors [21]. In addition, numerous web-based tools have been described, such as the PaccMann web service, which can estimate the chemosensitivity of a cancer cell line by integrating both cancer cell and chemical structure features [22], and CDRscan [23], a deep learning model that assesses anticancer drug responsiveness based on large-scale drug screening assay data. It employs a two-step convolution architecture, where the genomic mutational fingerprints of cell lines and the molecular fingerprints of drugs are processed individually then merged by ‘virtual docking’. An analysis of the extrapolation capability revealed a high accuracy (R^2^ > 0.84; AUROC > 0.98). The tool was applied to a large set of approved drugs, allowing the identification of 14 oncology and 23 non-oncology drugs. DeepIC_50_ [24], a one-dimensional convolution neural network model designed to predict drug responsiveness classes, was based on a large set of features. As a result, it showed better cell viability (IC_50_) prediction accuracy in pancancer cell lines over two independent cancer cell line datasets. More recently, pdCSM-cancer, which uses a graph-based signature representation, has been used to estimate the antiproliferative activity against multiple cancer cell lines [25]. 

In this work, considering our expertise in using molecular descriptors for biological purposes [26,27,28,29,30], we propose a new in silico Antiproliferative Activity Predictor (AAP) tool that can predict the antiproliferative activity of compounds against the NCI60 panel. The protocol, which is freely available on the DRUDIT web service (https://www.drudit.com (accessed on 15 November 2022) [28]), was based on both the molecular descriptors and in vitro antiproliferative data of tested compounds belonging to the NCI database.

In this article, the computational functions and the validation of the AAP tool are discussed in detail. Since our research group has focused on the synthesis of new small molecules with anticancer properties for many years [31,32,33], we decided to evaluate the performance of the AAP protocol by predicting the anticancer potential of an in-house small molecule database. The anticancer properties of several curcumin-like derivatives, some of which had already been evaluated as neuroprotective agents, were investigated [34,35,36]. To validate and confirm the AAP in silico data, in vitro antiproliferative assays of selected compounds were performed as a part of the NCI60 DTP screening program.

## 2. Results and Discussion

### 2.1. NCI60 Antiproliferative Activity Predictor Tool

The Antiproliferative Activity Predictor (AAP) tool is a new molecular-descriptor-based protocol for predicting the anticancer activities, expressed as GI_50_, of small molecules against the NCI60 panel. The AAP has been included as a module in DRUDIT, a web service that has already proven to be a reliable and valuable support for the development of new small molecules with biological activities [28,37].

#### 2.1.1. Description of the Tool Learning Process

**Figure 1:**
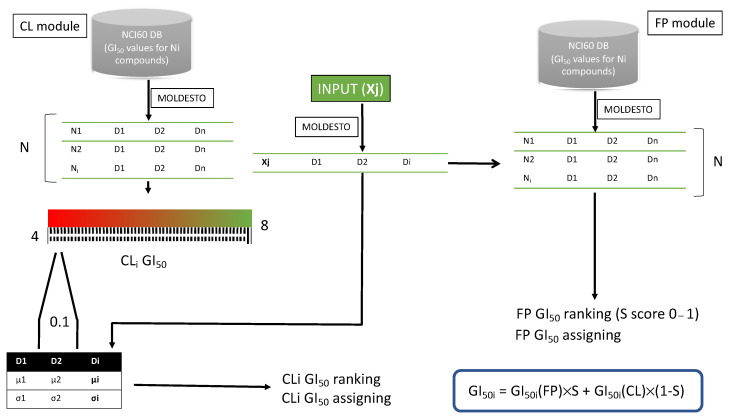
Flowchart of the antiproliferative predictor protocol: GI_50i_ is the GI_50_ value for cell line, S is the fingerprint score, GI_50i_(FP) is the GI_50_ value predicted by the FP module, and GI_50i_(CL) is the GI_50_ value assigned by the CL module.

The AAP tool consists of sequential steps, as reported in the flowchart (Figure 1).

**Figure 2:**
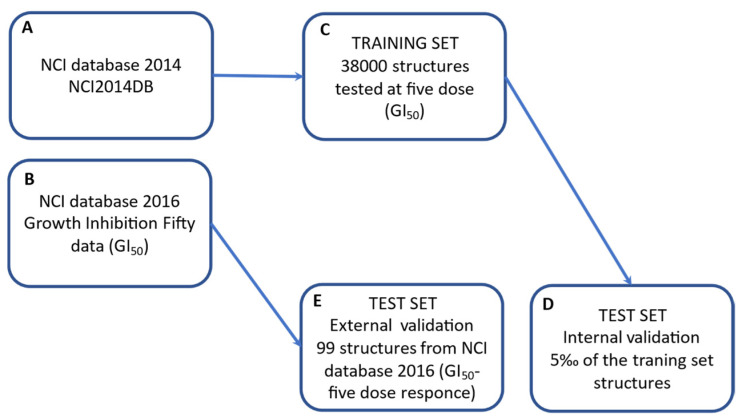
NCI selection data used in the AAP tool: compounds screened in five-dose assay and published until 2014 were used as training set; the 5‰ of these structures was used for the internal validation of the protocol (panels **A**,**C**,**D**); those structures screened in five-dose assay and published until 2016 were used as test set to evaluate the predictive performance of the entire protocol (panels **B**,**E**).

First, the NCI60 database, which contains in vitro data on the antiproliferative effects, expressed as GI_50_, of many compounds, was selected [10] (Figure 2).

From the thousands of structures tested by the NCI (Figure 2A), those that were screened in a five-dose assay were selected by considering the experimental GI_50_ values (Figure 2C). Then, according to publication dates, they were split into a training set (Figure 2C, data published until September 2014, referred to as NCI2014DB), which was used to build the model, and a test set (Figure 2E, data published until June 2016, referred to as NCI2016DB (Figure 2B)), which was used to validate the tool [38].

The AAP protocol results were obtained from the weighed contributions of two “modules”, called Finger-Print (FP) and Cell-Lines (CL); these contributed through a series of well-considered steps (Figure 1) to assign -logGI_50_ values (indicated as GI_50_ in the following sections) to input structures against the NCI60 panel cell lines (L_n_).

Preliminarily, a set of molecular descriptors (D_1_, D_2_, D_3_, and D_n_) was calculated for the training set (38 k structures, N, from NCI2014DB, Figure 2C). The molecular descriptor calculation was performed by MOLDESTO software (Supporting Information S1 contains a list of molecular descriptors implemented in MOLDESTO; see the Methods Section), which calculates more than 1000 1D, 2D, and 3D molecular descriptors (D_i_).

Then, using the molecular descriptors matrix (structures versus molecular descriptors) the above-described modules (FP and CL) were built as described below.

The **FP module** relies on structure similarity. It matches the molecular descriptor sequence of the input structure D_i_(X_j_) (Figure 1) with the D_i_ sequences of the structures belonging to the training set and assigns the S score as follows:

S = N(D_i_(X_j_))/N(D_i_); (1)

where N(D_i_(X_j_)) is the number of the molecular descriptors, D_i_(X_j_), which has a value in the range D_i_ ± D_i_ × 0.05, and N(D_i_) is the number of the molecular descriptors used.

By ranking according to the S score, the protocol assigns to the input structure X_j_ the experimental GI_50_ values of the best-scored structure of the training set (GI_50_(FP)). If experimental GI_50_ data of the training set structure are missing for one or more cell lines, a GI_50_ value is not assigned.

The **CL module**, on the other hand, is based on the cell lines. For each of the sixty NCI cell lines, 42 templates (CL_i_) were built: 40 for the GI_50_ values in the range of 4–8 (0.1 units each: 4.0–4.1; 4.1–4.2;….;7.9–8.0) and 2 for GI_50_ values <4 and >8.

**Figure 3:**
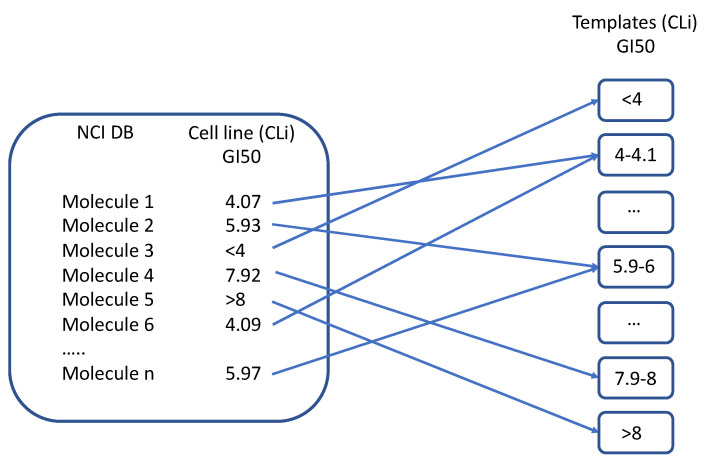
Template building process.

With this aim, the structures of the training set were assigned to each template according to the experimental GI_50_ values. In detail, all the structures that, for the specific cell line CL_i_, had a GI_50_ value in the appropriate range were assigned to the related template (Figure 3).

Then, the mean (μ_i_) and standard deviation (σ_i_) were computed for all molecular descriptors, considering the structures belonging to each template.

**Figure 4:**
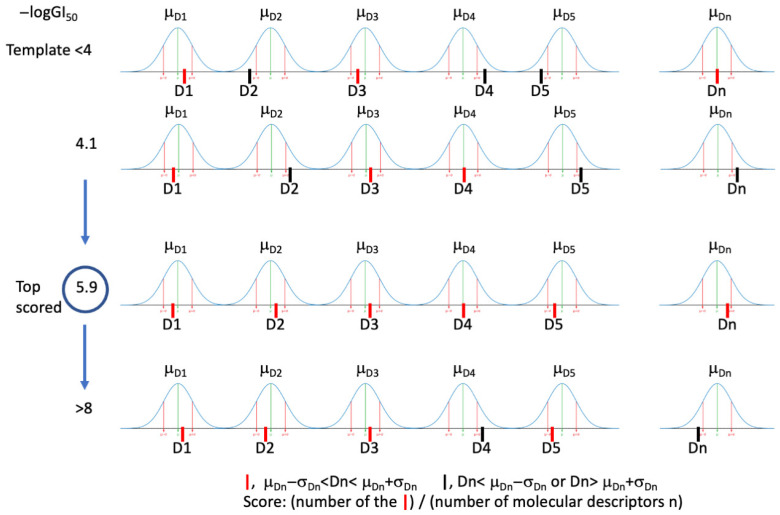
Graphical representation of GI_50_ prediction by the CL module for a cell line. D1, D2…Dn: molecular descriptors values for the input structure.

Thus, for each of the sixty cell lines, the molecular descriptor values of the input structure X_j_ were matched with the 42 cell line templates. The input structure X_j_ was assigned the GI_50_ value (GI_50_(CL)) of the corresponding template with the higher matching score (Figure 4).

This score was assigned by considering the sequence of D_i_ values of the input structure X_j_ matched to the sequence of the µ_i_(D_i_) ± σ_i_(D_i_) range of the cell line template. If a D_i_ value was in the range, a value of 1 was assigned; otherwise, a value of 0 was assigned. The sum of these binary scores, normalized to all molecular descriptors, gave the CL scores (Figure 4) and consequently the higher value, allowing GI_50_(CL) assignation (Figure 4).

Once an input structure, X_j_, was uploaded into the DRUDIT tools interface, MOLDESTO optimized the geometry in vacuo and calculated the molecular descriptors described above for the training set. Then, the molecular descriptor values were submitted to the FP and CL modules.

The output data from these modules were weighted as shown below:

GI_50i_ = GI_50i_(FP) × S + GI_50i_(CL) × (1 − S) (2)

where GI_50i_ is the GI_50_ value for that cell line, S is the fingerprint score, GI_50i_(FP) is the GI_50_ value predicted by the FP module, and GI_50i_(CL) is the GI_50_ value assigned by the CL module.

From this formula, if the structural similarity between the input structure and the best-scored structure of the training set was high (S score close to 1), it was assumed that the biological activity of the input structure was very similar to that of the compound from the training set (a similar structure could correspond to a similar biological activity). When S = 1, the input structure was included in the training set. Thus, the predicted GI_50_ corresponded to the experimental values. Instead, if S was not close to 1, GI_50_(CL) contributed more to the overall result, according to the S value. When the GI_50_(FP) was not available (unavailable data from the NCI screening), the GI_50_ corresponded to the GI_50_(CL).

#### 2.1.2. Validation of the AAP Tool

The predictive ability of the AAP was validated by internal and external validation. **Internal validation**: First, 5‰ of the training database structures (193 molecules), randomly selected from NCI2014DB (Figure 2D, Supporting Information S2), were used to validate the CL module by matching the calculated GI_50_(CL) values with the experimental GI_50_ data. Because these structures were used to generate the AAP protocol, their experimental GI_50_ values were indicated by the FP protocol, except for those that were not available (the experimental GI_50_ values are listed in Supporting Information S3; an empty box in the matrix indicates unavailable experimental data). The 193 structures were clustered into three groups according to their GI_50_ values: the most active compounds (more than 40/60 GI_50_ values equal to 8); the structures with GI_50_ values in the range of 4–8; and the cluster of less active/inactive compounds with GI_50_ values close to 4.

Therefore, GI_50_(CL) values were first calculated for the selected structures by setting the DRUDIT parameters (see the Methods Section for the meaning of the DRUDIT parameters). This step had two aims: it allowed us to verify the predictive capability of the CL module and, more importantly, to tune the DRUDIT parameters for the best prediction of antiproliferative activity for new compounds. Thus, runs 1–18 were performed by modulating the values of the parameters N, Z, and G, as reported in Table 1. The 18 outputs for the CL module are reported in Supporting Information S4).

The eighteen matrices from CL were matched with the experimental GI_50_ values to obtain 18 new matrices in which the |DTV (GI_50_)|, i.e., the absolute deviation of the calculated GI_50_(CL) value from the experimental GI_50_ value, was reported for each structure. Furthermore, for each entry, the average |DTV(GI_50_)| for all runs was examined. 

From the analysis of these data, it appears that the protocol allows the identification of potentially inactive or moderately active structures (below <4 or in the range of 4–7) with a remarkable degree of reliability, while it is less effective for structures with high activity (in the range of 7–8) but with acceptable errors. 

Moreover, it was demonstrated that the quality of the prediction was closely related to the amount of available biological data used to build the model. Since the number of highly active compounds (7 < GI_50_ < 8) was very low with respect to inactive or moderately active compounds, the prediction was negatively affected (higher error). Thus, the M19-MEL melanoma cancer cell line was kept out of the analysis due to a lack of sufficient biological data.

The matrix of GI_50_ values provided by each run was further elaborated to calculate the overall average |DTV(GI_50_)| for each cancer cell line. SK-MEL-28, belonging to the melanoma panel, gave the best predictions (|DTV(GI_50_)| of 1.14).

To select the optimized DRUDIT parameters, |DTV(GI_50_)| was calculated for each GI_50_(CL) matrix (Table 1). The parameters of run 1 (N = 240, Z = 50, G = a) were identified as the best, with an overall average error of 1.22 (Table 1). In this run, the renal cancer panel was the best prediction, with an average error of 1.18. The full results are reported in Supporting Information S5.

**External test validation**: A set of 99 molecules was collected from NCI2016DB (Figure 2B, see Methods Section for database selection and Supporting Information S6). Their known GI_50_ values were compared with the predicted ones, which were calculated using the optimized DRUDIT parameters (run 1, see above). The output matrix showed an interesting scenario with a |DTV(GI_50_)| of 0.87 and excellent predictions for structures with low activity (GI_50_ > 4 for at least 40 cell lines). On the other hand, significant errors were recorded for structures with high experimental GI_50_ values (GI_50_ above or close to 8). This evidence confirmed the capability of the protocol to better predict GI_50_ values for low-activity molecules. The average errors for each cancer cell line were also calculated, and they showed excellent prediction for the breast cancer panel (average error of 0.77 for the panel), especially against MDA-MB-231-ATCC (average error for the cancer line of 0.64).

**Figure 5:**
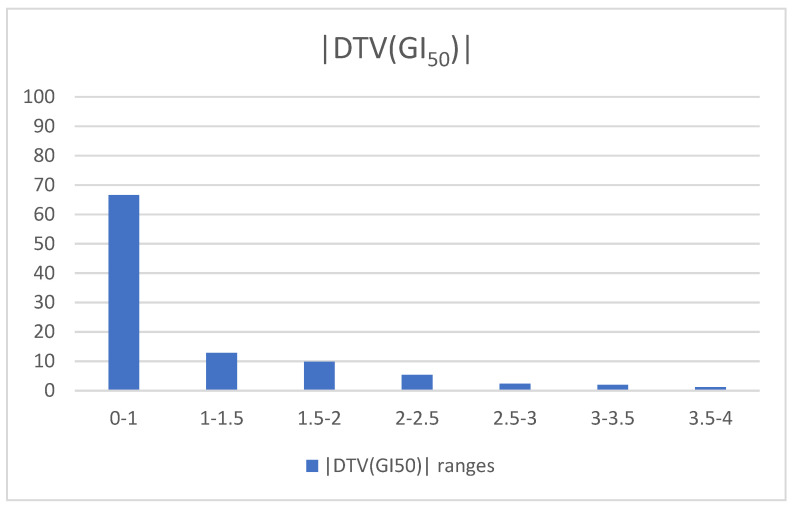
|DTV(GI_50_)| vs. frequencies of the data for the external test validation.

Analyzing the |DTV(GI_50_)| for all selected databases, considered by ranges, it was found that the protocol was able to assign the correct value returning ÷1 for 65% of the data (Figure 5).

A matrix that compares the experimental GI_50_ values with the calculated ones for all structures is reported in Supporting Information S7, in addition to the complete data analysis.

With the aim to demonstrate the relevant contribution of the CL module in the prediction, we also compared the experimental GI_50_ values with the calculated GI_50_(FP) values. The total average |DTV(GI_50_)| of 0.95, which is higher than that obtained by combining the predicted GI_50_ values given by both modules, shows that the CL module improves accuracy and leads to better predictions (Supporting Information S8). 

#### 2.1.3. Parameter Optimization for Cell Line/Subpanel Activity Prediction

Tuning the DRUDIT parameters (N, Z, and G) could also allow the optimization of the prediction for a specific cell line or subpanel (all the following results are shown in Supporting Information S9).

With this aim, the analysis was addressed to determinate the best combination of parameters (1–18) for the nine NCI subpanels and for each cancer cell line, as described above.

Then, the average |DTV(GI_50_)| values obtained for the cancer cell lines in all runs were analyzed (Supporting Information S5).

Regarding the optimization of parameters for each cancer cell line, SK-MEL-28 (melanoma panel) gave the best prediction, with an average error of 0.97 for parameter combinations 2 and 3. The full results are shown in Table 2.

To identify the best parameter combination for each panel, the average |DTV(GI_50_)| values of cell lines obtained in each run were grouped by the panel, and the average |DTV(GI_50_)| for the entire panel was calculated for runs 1–18. The results of the best combinations of parameters for each panel are given in Table 3.

The renal cancer panel resulted the best prediction of all, with an average error of 1.15 using parameter combination 10.

#### 2.1.4. Application of the AAP Tool for the Virtual Screening of an In-House Structure Database

**Figure 6:**
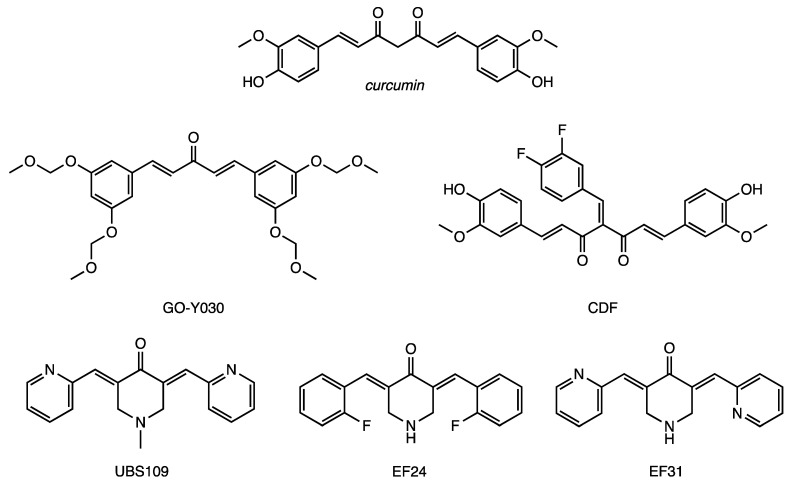
Curcumin and curcumin-like biologically active compounds.

The predictive capability of the AAP tool was exploited in the analysis of an in-house small molecule database to select compounds to be submitted to the NCI-DTP screening program. Recently, a few curcumin-like compounds were designed and biologically evaluated for neuroprotective and anti-Alzheimer properties, showing interesting results (Figure 6) [34,35,36]. 

Since curcumin has also been extensively studied in recent decades for its significant anticancer activity against various malignant cell types [39], the evaluation of curcumin-like compounds as antiproliferative agents against the NCI60 panel may be remarkably interesting. Indeed, many curcumin analogues have already been studied as antiproliferative agents, and some of them (e.g., EF24, UBS109, and CDF) showed higher activities and improved drug-like properties compared to curcumin (Figure 6) [40,41,42].

**Figure 7:**
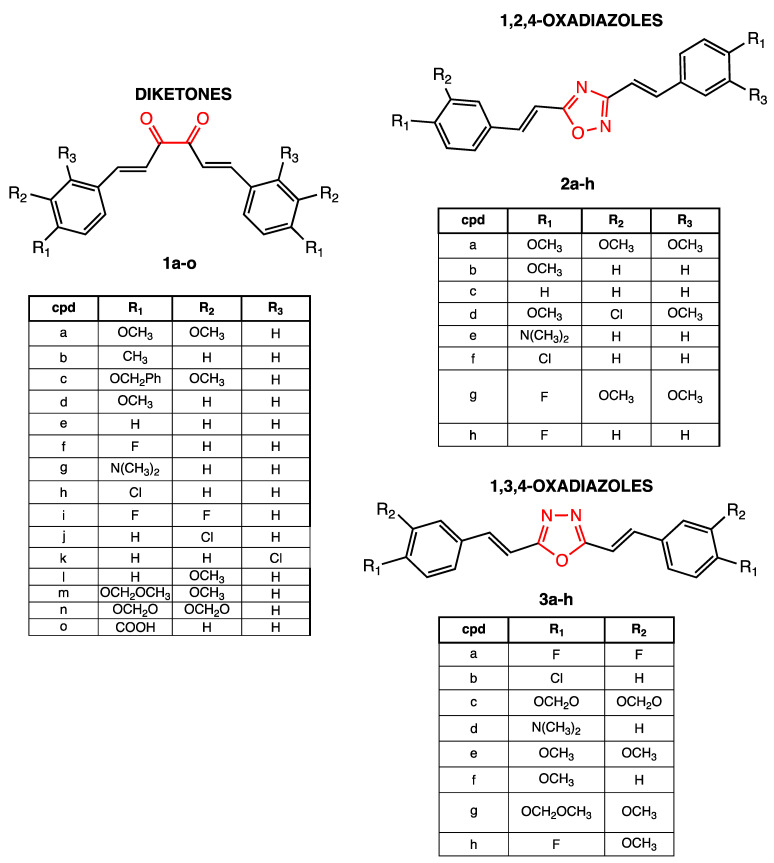
In-house structure database of curcumin-like compounds investigated by the AAP tool.

In detail, the selected in-house database included three different subclasses of curcumin-like derivatives, as reported in Figure 7: 1,2-diones (**1a**–**o**); 1,2,4-oxadiazoles (**2a**–**h**); and 1,3,4-oxadiazoles (**3a**–**h**). They were developed by replacing the symmetrical β-diketone core of curcumin, which is responsible for unfavorable physicochemical properties and a weak pharmacokinetic profile [43,44,45,46,47,48], with stacked moieties such as heterocycles or α-diketones; these replacements have been shown to improve stability, solubility, oral absorption, and bioavailability [49].

After selecting the molecule database, the next step was to tune the parameters of the AAP protocol. As mentioned earlier, the GI_50_ calculation can be targeted to a specific cell line or class of compounds by optimizing the parameters N, G, and Z. In this light, curcumin, tested by NCI (NSC code 32982) and included in the training set (NCI2014DB), was selected as a reference compound to determinate the best combination of parameters for the CL module. The tuning was performed with the parameters in the ranges 250 < N < 800 and 50 < Z < 100 while considering a, b, or c for the G function. Eighteen runs were started, following the procedure described above for the internal validation (the combinations are reported in Table 1). Consequently, the total absolute deviations from the experimental GI_50_ values were calculated for each run, and the set of N = 760, Z = 50, and G = c was identified (total |DTV(GI_50_)| = 0.44) and applied to the AAP tool for the selected database (see Supporting Information S10).

The output of the AAP tool with the calculated GI_50_ values of the 39 in-house compounds is listed in Supporting Information S11. The AAP GI_50_ values of the curcumin-like analogues were compared with the experimental ones determined by the NCI for the curcumin lead compound (Supporting Information S11). The analysis of the GI_50_ mean of each compound for the full panel highlighted several curcumin-like molecules with predicted antiproliferative activities better than that of the reference curcumin (average GI_50_ value of 5.17), such as **1a** (5.47), **1e** (5.65), and **1m** (5.82) for the diones class; **2a** (5.49), **2d** (5.57), and **2g** (5.44) for the 1,2,4-oxadiazole class; and **3a** (5.72), **3e** (5.49), **3g** (6.27), and **3h** (5.47) for the 1,3,4-oxadiazole class. To test the consistency of the AAP protocol, both compounds classified as active and inactive (with a GI_50_ mean of less than 4.5) were synthesized and subsequently proposed to the NCI for the in vitro evaluation of the antiproliferative activity against the NCI60 human tumor cell lines, assuming that a reliable protocol must be able to identify both active and inactive compounds. 

### 2.2. Chemistry

**Scheme 1:**
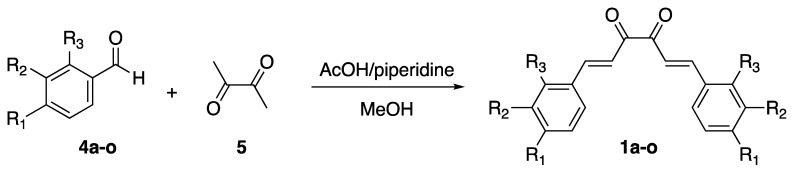
Synthesis of cinnamils **1a**–**o**.

The three classes of compounds of types **1**, **2**, and **3** were synthetized as previously described in the literature [35,36,50]. Cinnamils (1,6-diarylhexa-1,5-diene-3,4-diones) **1a**–**o** were achieved by the aldol condensation of aromatic aldehydes **4a**–**o** with diacetyl **5**, leading to the formation of the double bonds, both with E geometry (Scheme 1) [51].

**Scheme 2:**
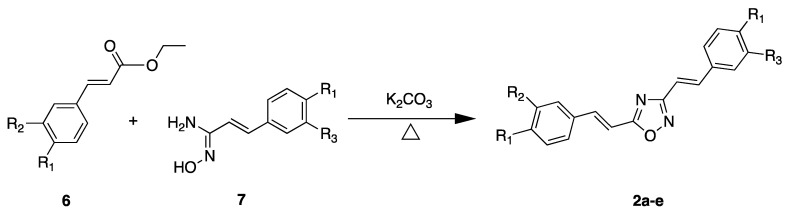
Synthesis of 1,2,4-oxadiazole derivatives **2a**–**e**.

The 1,2,4-oxadiazole derivatives **2a**–**e** were synthesized following the conventional amidoxime synthetic strategy [52], starting from the esters **6** and the amidoximes **7** (Scheme 2).

**Scheme 3:**
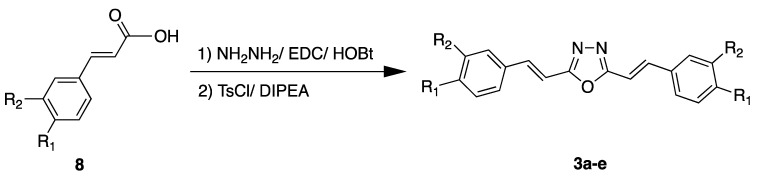
Synthesis of 1,3,4-oxadiazole derivatives **3a**–**e**.

The 1,3,4-oxadiazoles **3a**–**h** were achieved through the one-pot synthesis described in Scheme 3. Diacylhydrazine intermediates were obtained by the reaction of the cinnamic acid analogues **8** and hydrazine. The subsequent cyclization led to the isolation of the regio-isomers (E) **3a**–**h** in good overall yields [34] (synthetic details and spectroscopic characterization for all compounds are reported in Supporting Information S12) [34,35,36].

### 2.3. Biological Assays: NCI60 Human Tumor Cell Lines Screen Selected Compounds

All synthesized curcumin-like compounds were submitted to NCI cell-line-based in vitro screening for anticancer drugs. As described in the Methods Section (compound selection guidelines paragraph), the NCI applied specific criteria for compound selection. In the case of analogues, the selected compounds were those that were the most representative of the series and had significant structural novelty compared to the NCI collection.

**Figure 8:**
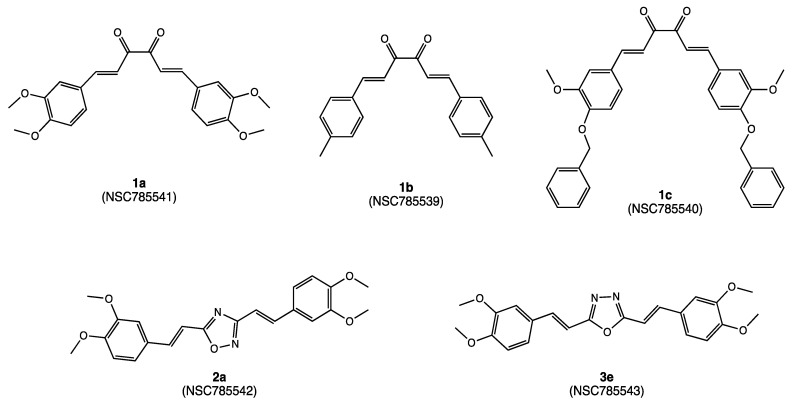
Chemical structures of the five curcumin-like compounds selected by NCI for the one-dose antiproliferative assay.

From the three series of curcumin-like derivatives, five molecules were selected by the NCI for the in vitro biological screening: **1a** (NSC785541), **1b** (NSC785539), and **1c** (NSC785540) for the dione series; **2a** (NSC785542) for the 1,2,4-oxadiazole series; and **3e** (NSC785543) for the 1,3,4-oxadiazole series (Figure 8). 

#### 2.3.1. One-Dose Antiproliferative Assay

The NCI screening protocol consisted of a preliminary one-dose assay (concentration of 10 μM) against the full NCI60 panel. Compounds that met the NCI selection criteria and had a significant growth-inhibitory effect on a minimum number of cell lines proceed to the five-dose screening (experimental details are described in the Methods Section). The results are expressed as the percent of growth (G%) of the treated cells when compared to the untreated control cells. This parameter accurately expresses the anticancer potential of the drug. At G% > 100, the compound has no effect on cancer cell proliferation (inactive). In the range, the compound inhibits cell proliferation by a percentage indicated by 100-G%. When G% is <0, the compound is cytotoxic and lethal to the cancer cells. To graphically represent the most sensitive panels/cell lines, a mean growth percentage is also provided.

The mean G% values of the five selected compounds for each of the nine subgroups of cancer cell lines are shown in Table 4. The full results and the mean graphs from the one-dose screening are reported in Supporting Information S13.

Consistent with the AAP-predicted GI_50_ values for these compounds, the biological data confirmed the dimethoxy-dione **1a** (NSC785541) and the dimethoxy-1,3,4-oxadiazole **3e** (NSC785543) to be the most active curcumin-like derivatives. They showed remarkable overall average G% values (26.29 and 26.93, respectively) and an average inhibition of cell growth of about 75% against the full NCI60.

Compound **1a** demonstrated high lethality against the colon cancer panel (average G% of −16.06), with the highest cytotoxic effect on cell lines HCT-116 (G% = −74.85) and HT29 (G% = −35.20) and against leukemia panel, with an average G% of 14.84 and an almost complete blockade of cell growth (G% ~0) for cell lines K562, RPMI-8226, and SR (see Supporting Information S13). Notable data were obtained for the LOX IMVI cell line (melanoma panel), with a G% of −66.41, and against the RXF 393 (renal cancer panel), with a G% of −63.27.

Similarly, dimethoxy-1,3,4-oxadiazole **3e** showed a strong antiproliferative effect against the colon cancer panel but with lower toxicity; it exhibited a G% close to 0, implying an arrest of cell growth with low/no lethality against the most sensitive cell lines, HCT116 and HT-29. Furthermore, the leukemia, melanoma, and CNS cancer panels also showed interesting sensitivity to the tested compound. Therefore, according to the selection criteria of the DTP NCI protocol, these two molecules progressed to the full five-dose assay (see the next section).

The other three compounds tested in the NCI one-dose protocol, **1b**, **1c,** and **2a**, generally exhibited less inhibitory activity, with an average G% close to 100.

These results confirm the AAP in silico data, according to which the compounds **1b** and **1c** were predicted to be almost inactive, with mean GI_50_ values of 4.38 and 4.54 (low millimolar range), respectively.

On the other hand, when it was analyzed for its antiproliferative effect on specific human cancer cell lines, dione **1b** reduced the growth of the RPMI-8226 and MCF-7 cell lines by 55% and 76%, respectively (G% = 45.15 and 23.84) and induced a remarkable death in the HCT-116 colon cancer cell line (G% = −36.84). Compound **1c**, instead, exhibited a G% of 69.01 against HCT-116 colon cancer cells.

The 1,2,4-oxadiazole **2a**, which was predicted to be more active than curcumin, did not exhibit any appreciable anticancer activity against the NCI60 database, with the exception of HT29 colon cancer cells (G% of 56.32). This result was unexpected, considering that the isomer **3e** was selected for investigation in the five-dose screening. It could probably be suggested that the change from the 1,3,4-oxadizole to the 1,2,4-oxadiazole core affects, in particular, the ability of the compound to interfere with biological targets.

Therefore, both compounds **1a** and **3e** were selected for the five-dose screening to measure the GI_50_s, which permitted us to further validate our tool.

#### 2.3.2. Five-Dose Antiproliferative Assay for the Most Active Derivatives, **1a** and **3e**

The two selected compounds, **1a** and **3e,** were tested with the five-dose assay by measuring the percentage of cell growth at five different concentrations (from 10^−8^ to 10^−4^ M), as described in detail in the Methods Section. For each selected compound, NCI provided the measured GI_50_, TGI, and LC_50_ values against the NCI60 cell lines, with the corresponding mean graphs (see Supporting Information S14).

In the first part of this section, attention is focused exclusively on the GI_50_ values, as these data allowed us to further assess the predictive ability of the proposed AAP protocol.

The comparison of the average predicted GI_50_ with the experimental values obtained by NCI for the two compounds confirmed that the protocol was able to predict with high accuracy the range of activity of both compounds against the full NCI60 (for **1a**, the average predicted GI_50_ was 5.39, whereas the average experimental GI_50_ was 5.49; for **3e**, the average predicted GI_50_ was 5.41, whereas the average experimental GI_50_ was 5.28). 

To further analyze the performance of the AAP protocol, the predicted GI_50_ values were matched to the experimental values; moreover, the |DTV(GI_50_)| was computed for the two tested compounds. This allowed us to calculate the average absolute error values for the compounds, which were 0.39 and 0.40 for **1a** and **3e**, respectively (see Supporting Information S15), indicating the capability to assign a GI_50_ value with an error of less than one order of magnitude. 

In general, considering the mean |DTV(GI_50_)| for each panel, the AAP returned very low errors in activity prediction against specific panels, such as, for example, the prostate cancer panel for **1a** (average |DTV(GI_50_)| for the panel of 0.07) and the colon cancer panel for **3e** (average |DTV(GI_50_)| for the panel of 0.25). Furthermore, a detailed analysis of specific cell lines revealed that the protocol was able to predict GI_50_ values for some specific cell lines with remarkable precision: for **1a**, the |DTV(GI_50_)| against HL-60TB and NCI-H322M was only 0.02; for **2a**, a |DTV(GI_50_)| of only 0.03 was computed for HT-29 and OVCAR-5. 

On the other hand, BT-549 (breast cancer panel) gave the worst predictions for both compounds, with |DTV(GI_50_)| values of 2.21 and 2.41; this evidence is consistent with the results presented in the previous section (tool validation), where this cell line showed the highest error. As previously demonstrated, high prediction error can be attributed to the lack of biological data for selected cell lines. This was confirmed by looking at the GI_50_(FP) values assigned to both compounds: the structure selected with the best score in the FP module was not tested against BT-549. Thus, the final GI_50_ relied solely on the CL protocol rather than combining outputs from both modules, which may have drastically affected the quality of the prediction.

**Figure 9:**
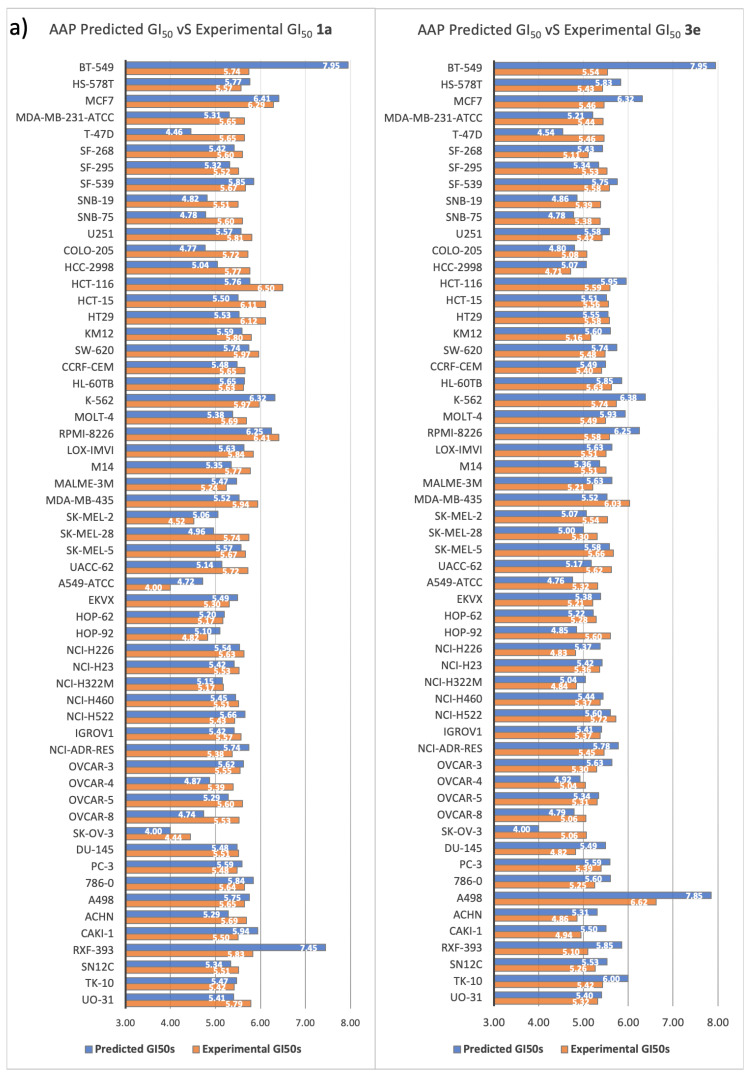

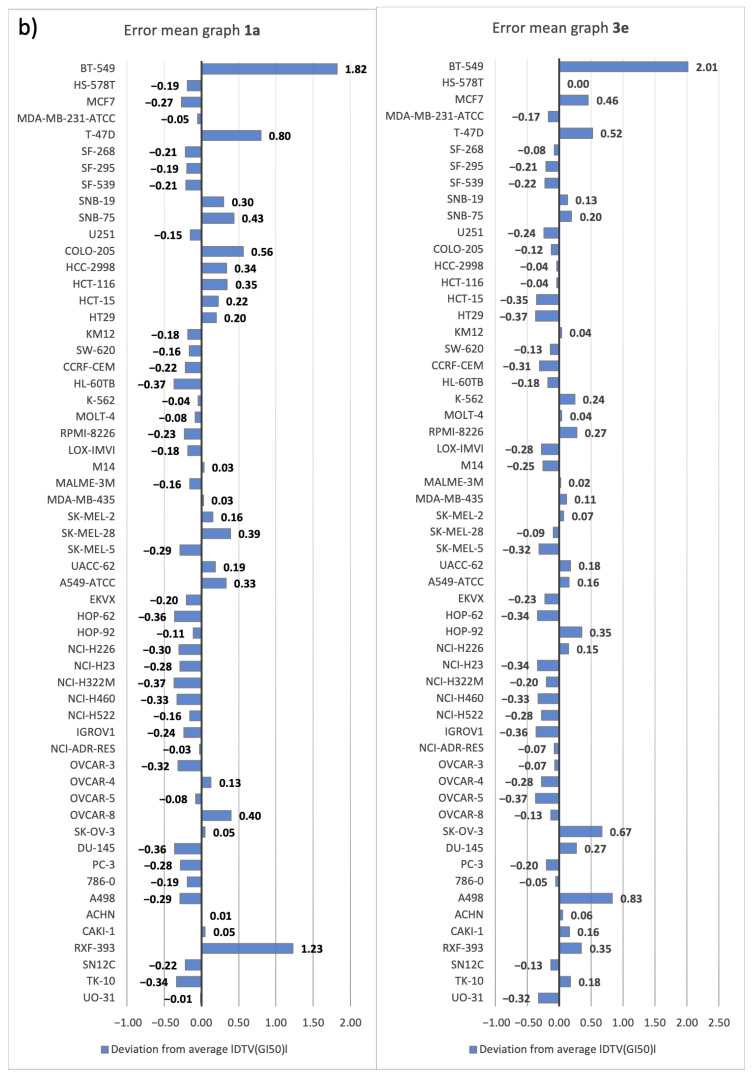
(**a**) Comparison between AAP-predicted GI_50_ values and the corresponding experimental GI_50_ values measured by NCI for the two selected compounds, **1a** and **3e** (inside each bar, the corresponding GI_50_ value is indicated). (**b**) Mean error graphs for the two selected compounds, **1a** and **3e**.

In Figure 9a, the two bar graphs depicting the comparisons between predicted GI_50_ and experimental GI_50_ are reported to graphically appreciate the excellent performance of our protocol; in Figure 9b, instead, the mean error graphs are shown to highlight the cell lines for which the highest/lowest errors were recorded.

In Table 5, the full NCI output data, (GI_50_s, TGI, and LC_50_ values) of compounds **1a**, **3e,** and curcumin are shown (the mean graphs and the full NCI schedules are reported in Supporting Information S14). The analysis of the values allowed us to highlight the noteworthy antiproliferative activity of the two selected curcumin-like compounds compared to curcumin; indeed, the protocol predicted a higher activity with respect to the parent compound. 

Through the evaluation of the GI_50_ values (the most diagnostic parameter used to compare antiproliferative potential), it emerged that the average GI_50_ values were higher for both the tested compounds, in the high micromolar range, than for curcumin (5.59 for **1a** and 5.37 for **3e** vs. 5.16 for curcumin), confirming the in silico predictions. 

With respect to the tumor subpanels, the most active compound, the dione **1a**, proved to be particularly effective against leukemia, colon cancer, and breast cancer. In fact, the calculated average GI_50_ values for these subpanels (5.87, 6.00, and 5.79, respectively) were always higher than the overall average GI_50_ value (5.59). In detail, among these subpanels, several cell lines showed remarkable sensitivity to the compound, with excellent GI_50_ values in the low micromolar range: RPMI-8226 (6.41), HCT-116 (6.5, the most sensitive cell line), HCT-15 (6.11), HT-29 (6.12), and MCF-7 (6.29). Moreover, the analysis of TGI values showed that the dione **1a** was the most active compound of the series (overall average TGI of 4.81), confirming its selectivity against the aforementioned subpanels and cell lines, especially against colon cancer (average TGI of 5.48). This trend was also confirmed at the LC_50_ level, with a strong cytotoxic effect against colon cancer cell lines (average GI_50_ of 4.91 for colon cancer). Interestingly, it is important to highlight the very low toxicity against RPMI-8226 (LC_50_ < 4), which proved to be one of the most sensitive (GI_50_ in the sub-micromolar range), demonstrating the high potency and low toxicity of the compound against this cell line.

The curcumin-like **3e**, although less active than the previous one, was more effective than curcumin. Remarkable results were obtained against the leukemia subpanel, with a panel average of 5.57, much higher than the average value for the full NCI60 (5.37). Moreover, the oxadiazole derivative exhibited high potency against two cell lines, MDA-MB-435 (melanoma) and A498 (renal cancer), with excellent GI_50_ values in the low micromolar range (6.03 and 6.61, respectively). In terms of the TGI level, it was slightly less effective than curcumin, but the average LC_50_ value of 4.01, even against the most susceptible cells, indicated high potency with low cytotoxicity, even at high concentrations.

**Figure 10:**
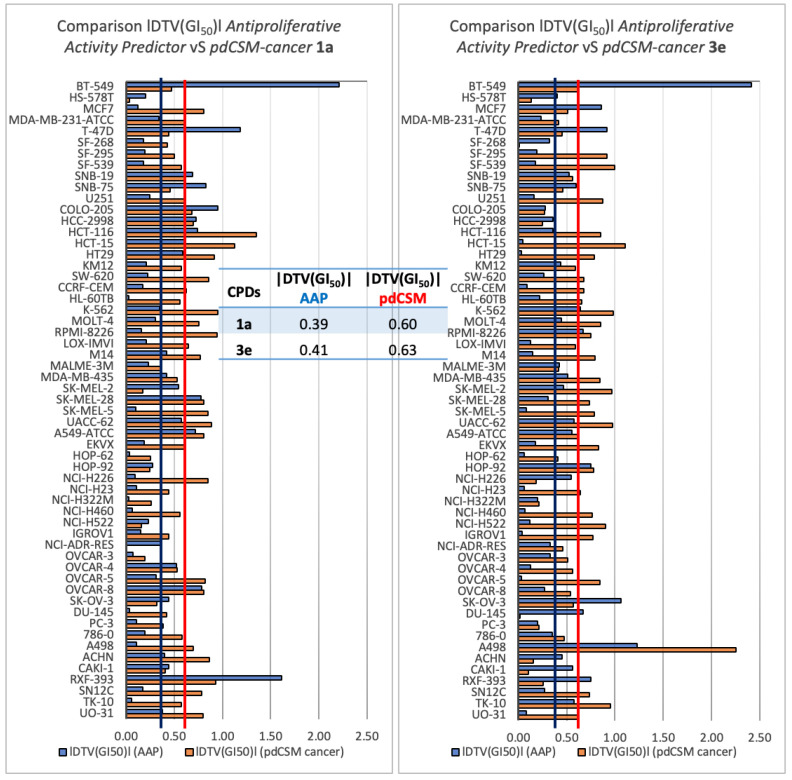
Performance of AAP vs. pdCSM-cancer tools: blue and red vertical lines indicate the |DTV(GI_50_)| for the AAP and pdCSM tools, respectively.

To further evaluate the prediction capability of the AAP, the pdCSM-cancer tool was selected as a comparative approach. Thus, structures **1a** and **3a** were submitted to pdCSM-cancer and compared with the results obtained by the AAP. The obtained data are reported in Figure 10.

It is noteworthy that the AAP tool showed |DTV(GI_50_)| values of 0.39 and 0.41 for the compounds **1a** and **3a**, while the pdCSM-cancer tool showed values of 0.60 and 0.63, respectively. The cell line that showed a low grade of reliability was BT-549, not only in these cases in the study but also in the external test screening. A change in dataset could solve this problem.

## 3. Materials and Methods

### 3.1. Computational Studies

#### 3.1.1. Hardware

The DRUDIT web service runs on four servers that are automatically selected according to the number of jobs and online availability. Each server can support up to 10 simultaneous jobs, while the exceeding jobs are placed in a queue. 

#### 3.1.2. Software

DRUDIT consists of several software modules implemented in C and JAVA and running on MacOS Mojave.

#### 3.1.3. Database Selection and Dataset Building

The NCI60 database, containing both antiproliferative and chemical data of thousands of compounds, was selected as a reliable source for the building of the protocol.

In detail, since the presented tool is based on molecular descriptors, the 2D chemical structures of the NCI-tested compounds (.mol files, only available until the June 2016 release) and the corresponding growth inhibition data were retrieved from the NCI website (284,176 chemical structures) [38,53]. Among these thousands of compounds, only those tested with the five-dose assay, which provided GI_50_ data, were selected to build and validate the model. In particular, the structures were split into two sets: a training set containing more than 38 k compounds released until 2014 (NCI2014DB) was used to build the protocol, and a test set containing about 100 compounds that were first released in 2016 (NCI2016DB) was used to validate the AAP tool.

#### 3.1.4. MOLDESTO: A New Software for Molecular Descriptor Calculations

MOLDESTO (molecular descriptor tool), as described previously [28], is a software tool implemented in DRUDIT that represents the evolution of our expertise in the calculation/manipulation of molecular descriptors [30]. It is currently able to calculate more than 1000 molecular descriptors (1D, 2D, and 3D) for each input structure (the full list of molecular descriptors calculated by MOLDESTO is reported in Supporting Information S1). The input structures can be drawn directly in the web interface or uploaded as commonly used molecule file formats (e.g., SMILES, SDF, Inchi, Mdl, and Mol2). The software is provided with a caching system to boost the calculation speed of previously submitted structures.

#### 3.1.5. DRUDIT Settings for Antiproliferative Activity Predictor (AAP) Tool

The AAP tool comprises the fingerprint (FP) and cell line (CL) modules, which cooperate simultaneously to assign the predicted GI_50_ values to an input structure. In each module, the performed calculation is dynamic; indeed, it can be modulated by appropriately tuning the values of the available parameters (three for each module, see below).

The FP module parameters are a choice of biological activity, such as GI_50_, TGI, LC_50_, or G% (in this work only the first choice was considered); N (-b), the best number of the dynamically selected molecular descriptors; Z (-m), the number of descriptors for which |v-m|/m < <value> applies (v: descriptor value, m: target mean); and G (-c), the max number of zero percentage values per descriptor. The DRUDIT parameters for the CL module are a choice of biological activity as GI_50_, TGI, LC_50_, or G% (in this work only the first parameter was considered); N (-b), the best number of dynamically selected molecular descriptors; Z (-m), the max number of zero percentage values per descriptor; and G (-f), the Gaussian smoothing function to be used (a, b, or c mode).

### 3.2. Chemistry

All solvents and reagents were used as received unless otherwise stated. Melting points were determined on a hot-stage apparatus. The ^1^H-NMR and ^13^C-NMR spectra were recorded at the indicated frequencies; the residual solvent peak was used as a reference. Chromatography was performed using silica gel (0.040–0.063 mm) and mixtures of ethyl acetate and petroleum ether (fraction boiling in the range of 40–60 °C) in various ratios (*v*/*v*). Compounds **1d**–**o** [36], **2a**–**c** [36], **2d** [35], **3a**–**g** [36], and **3h** [35] were prepared as previously reported. Compounds **1a**–**c** and **2e**–**h** were prepared by adapting previously reported methods. The synthetic details and spectroscopic characterizations of all compounds are reported in Supporting Information S12.

### 3.3. NCI60 Antiproliferative Screenings

#### 3.3.1. Compound Selection Guidelines

The compounds to be screened were selected according to precise and rigorous guidelines; in general, submission was encouraged for molecules that bring some novelties (novel heterocyclic ring systems and privileged scaffolds) to the NCI collection and compounds that emerged from computer-aided drug design. In addition, in the case of a series of analogues, it was preferred to select only the one that was expected to provide the greatest information. On the other hand, the submission of compounds with the following features was discouraged: excessive flexibility; the presence of non-drug-like functional groups (nitro, nitroso, diazo, imine, etc.); and the presence of chemical portions that could affect the reliability of the assays (PAINS) [54].

#### 3.3.2. One-Dose Assay

All compounds submitted to NCI were first assayed against the NCI60 DB in a one-dose screen (concentration of 10^5^ M); this kind of assay aims to determine the G% (growth inhibition percent) of the compounds against the considered cells. The results were plotted in a one-dose graph showing the G% of the single compound against the 60 cell lines. This first assay was considered passed only for the most promising compounds (satisfaction of predetermined threshold criteria); in this case, the compound passed to the five-dose screen (for further experimental details about the standardized assay procedures, see [55,56]).

#### 3.3.3. Five-Dose Assay

The most active compounds were submitted to a multiple-dose screen using five different concentrations (ranging from 10^−8^ to 10^−4^ M). The dose–response curves obtained from this assay permitted the extrapolation of the GI_50_ (the molar concentration of the compound that inhibits 50% of cell growth), TGI (the molar concentration of the compound leading to total inhibition of cell growth), and LC_50_ (the molar concentration of the compound that induces 50% cell death) values of the selected compounds against each cancer cell line. For each of the mentioned parameters, a mean graph midpoint (MG_MID) was calculated, providing an average activity parameter over all cell lines (for further experimental details about the standardized assay procedures, see [55,56]).

## 4. Conclusions

In the field of antitumor drug discovery, in vitro antiproliferative assays still represent one of the most important tools for identifying new small molecules against cancer. In recent years, numerous projects and online databases have been launched aiming to collect both drug response and cellular data, with NCI60 undoubtedly being the best-known and most complete [1].

To avoid the high failure rate and the enormous number of resources invested to perform such intensive in vitro screenings, computational chemistry and biology, in the last few years, have sought to develop in silico techniques that are able to predict the antiproliferative potential of new small molecules in the early stage of the drug discovery pipeline.

In this light, we have presented the antiproliferative activity predictor (AAP), a new molecular-descriptor-based tool capable of reliably predicting the anticancer potential (expressed as GI_50_, the most diagnostic parameter to measure anticancer drug responses) of small molecule libraries against the full NCI60 DB.

Using both the structural and antiproliferative data (GI_50_) of thousands of compounds stored in the NCI DB, we applied our expertise in manipulating molecular descriptors to build two convergent modules, the FP (fingerprint) and the CL (cell line); as shown, these operate synergistically to assign GI_50_s values to the input structures. 

Both internal and external validation were performed to validate the CL module and the entire tool, demonstrating the reliability and the robustness of the proposed protocol. Interestingly, the possibility to appropriately tune the available parameters (N, Z, and G) allows researchers to address the screening to a specific subpanel/cell line or class of compounds. An important aspect that should be highlighted is the deep correlation between the quality of the prediction and the availability of biological data. In the case of the lack of sufficient GI_50_ data, as for a cell line (M19-MEL) or for some ranges of activity (structures with GI_50_ values in the range of 7–8), the prediction is negatively affected.

Moreover, the application of the AAP tool to quickly screen an in-house structure database of curcumin-like compounds permitted us to further corroborate the already obtained encouraging results: compounds **1a** and **3e**, predicted to be highly active by the protocol, were found to be significantly antiproliferative for several human cancer cell lines in the five-dose NCI screen. This further goal confirmed that the AAP could be an invaluable help in the in silico design of new effective anticancer small molecules, permitting the selection of the most promising molecules in the first phases of the drug design process.

It is worth noting that the integration of the AAP tool into our free and easy-to-use DRUDIT web service (available online at https://www.drudit.com (accessed on 15 November 2022)) provides an interesting tool for the entire medicinal chemistry community.

In the future, we plan to continuously update the training set with new NCI-tested compounds to cover more and more chemical spaces, activities, and cell lines. Finally, the extension of the AAP’s potential for the calculation of more parameters, such as TGI or LC_50_, could also be interesting. The possibility to calculate not only the GI_50_ but also these parameters (evaluated in vitro by the NCI) could allow the identification and eventually the elimination of small molecules with toxicity profiles that are unacceptable for further development. 

## Figures and Tables

**Table 1:** Overview of DRUDIT runs for parameter tuning. Absolute deviation from true values (|DTV(GI_50_)|) is reported, and run codes (1–18) are given in parentheses.

Z	N	G		
		a	b	c
50	240	1.22 (1)	1.23 (2)	1.23 (3)
	500	1.22 (7)	1.30 (8)	1.31 (9)
	760	1.32 (13)	1.44 (14)	1.42 (15)
100	240	1.31 (4)	1.64(5)	1.72 (6)
	500	1.23 (10)	1.51 (11)	1.53 (12)
	760	1.28 (16)	1.42 (17)	1.44 (18)

**Table 2:** Tuning of DRUDIT parameters for each cancer cell line.

PANELS	CELL LINES	RUN	AVERAGE |DTV(GI_50_)|
**Breast Cancer**	BT-549	4	1.35
	HS-578T	3	1.30
	MCF7	1/7	1.30
	MDA-MB-231-ATCC	7	1.22
	T-47D	7	1.16
**CNS Cancer**	SF-268	7	1.17
	SF-295	1	1.25
	SF-539	4	1.18
	SNB-19	7	1.15
	SNB-75	1	1.16
	U251	1	1.24
**Colon Cancer**	COLO-205	10	1.13
	HCC-2998	1	1.09
	HCT-116	2/7	1.13
	HCT-15	2	1.21
	HT29	1	1.14
	KM12	1	1.19
	SW-620	2	1.14
**Leukemia**	CCRF-CEM	7	1.13
	HL-60TB	7	1.22
	K-562	2	1.27
	MOLT-4	3	1.12
	RPMI-8226	10	1.12
	SR	2	1.28
**Melanoma**	LOX-IMVI	3	1.16
	M14	1/3	1.20
	MALME-3M	10	1.19
	MDA-MB-435	3	1.22
	SK-MEL-2	3	1.03
	SK-MEL-28	2/3	0.97
	SK-MEL-5	2	1.26
	UACC-257	2	1.07
	UACC-62	10	1.31
**Non-Small-Cell Lung Cancer**	A549-ATCC	3	1.18
	EKVX	1	1.02
	HOP-62	1/8	1.19
	HOP-92	10	1.21
	NCI-H226	7	1.07
	NCI-H23	4	1.16
	NCI-H322M	1	1.10
	NCI-H460	2	1.26
	NCI-H522	7	1.09
**Ovarian Cancer**	IGROV1	1/3	1.25
	NCI-ADR-RES	4	1.31
	OVCAR-3	1/4	1.22
	OVCAR-4	7	1.00
	OVCAR-5	16	1.02
	OVCAR-8	1	1.14
	SK-OV-3	7	1.18
**Prostate Cancer**	DU-145	10	1.19
	PC-3	2	1.19
**Renal Cancer**	786-0	10	1.16
	A498	1	1.21
	ACHN	7	1.19
	CAKI-1	1	1.11
	RXF-393	10	1.12
	SN12C	1/10	1.16
	TK-10	10	0.99
	UO-31	2	1.16

**Table 3:** Best parameter combinations for each panel.

PANELS	RUN	AVERAGE|DTV(GI_50_)|
**Breast Cancer**	1/3	1.37
**CNS Cancer**	1	1.23
**Colon Cancer**	1	1.19
**Leukemia**	2	1.23
**Melanoma**	3	1.18
**Non-Small-Cell Lung Cancer**	2/7	1.20
**Ovarian Cancer**	1	1.21
**Prostate Cancer**	1	1.23
**Renal Cancer**	10	1.15

**Table 4:** G% values determined for the five selected compounds against the NCI60 panel with the one-dose assay.

PANEL ^1^	1a	1b	1c	2a	3e
Leukemia	14.84	78.71	96.37	77.47	18.53
Non-Small-Cell Lung Cancer	61.79	98.02	95.47	84.39	29.86
Colon Cancer	−16.06	80.65	95.84	86.52	21.07
CNS Cancer	32.98	98.55	101.40	98.19	18.66
Melanoma	24.26	97.49	100.59	96.74	22.40
Ovarian Cancer	46.48	103.35	101.45	95.29	32.68
Renal Cancer	21.07	100.02	100.31	94.36	34.17
Prostate Cancer	33.06	102.04	101.62	88.89	40.34
Breast Cancer	18.21	86.77	99.62	88.93	24.71
*Overall average*	26.29	93.96	99.19	90.09	26.93

^1^ For each compound, the average G% values against the nine subpanels are reported.

**Table 5:** DTP NCI five-dose screening for compounds **1a**, **3e, and curcumin**.

PANEL	CELL LINE ^1^	1a			3e			Curcumin		
		GI_50_	TGI	LC_50_	GI_50_	TGI	LC_50_	GI_50_	TGI	LC_50_
**Leukemia**	CCRF-CEM	5.65	4.7	4	5.4	4	4	5.52	4.81	4
	HL-60(TB)	5.63	4.77	4	5.63	5.07	4	5.14	4.60	4.04
	K-562	5.97	4	4	5.74	4	4	5.51	4.26	4
	MOLT-4	5.69	4.72	4	5.49	4	4	5.33	4.75	4.12
	RPMI-8226	6.41	5.63	4	5.58	4	4	5.68	5.20	4
	*Panel average*	5.87	4.76	4	5.57	4.21	4	5.43	4.72	4.03
**Non-Small-Cell Lung Cancer**	A549/ATCC	4	4	4	5.32	4	4	4.89	4.50	4.11
	EKVX	5.3	4	4	5.21	4	4	4.82	4.45	4.10
	HOP-62	5.17	4	4	5.28	4	4	5.44	4.72	4.24
	HOP-92	4.82	4.08	4	5.6	4.63	4	NT	NT	NT
	NCI-H226	5.63	NT ^1^	4	4.83	4	4	4.73	4.27	4
	NCI-H23	5.52	4	4	5.36	4	4	5.25	4.50	4
	NCI-H322M	5.17	4	4	4.84	4	4	4.78	4.49	4.21
	NCI-H460	5.51	4.95	4	5.37	4	4	5.09	4.64	4.22
	NCI-H522	5.43	4.72	4	5.72	5.17	4.02	5.27	4.78	4.07
	*Panel average*	5.17	4.22	4.00	5.28	4.20	4	5.03	4.54	4.12
**Colon Cancer**	COLO-205	5.72	5.31	4.4	5.08	4.01	4	4.87	4.54	4.21
	HCC-2998	5.77	5.49	5.22	4.71	4	4	5.52	5.09	4.53
	HCT-116	6.5	5.88	5.39	5.59	4.75	4	5.53	5.03	4.28
	HCT-15	6.11	5.21	4.25	5.56	4	4	5.39	4.73	4.14
	HT-29	6.12	5.58	5.09	5.58	4.97	4	5.29	4.49	4
	KM12	5.8	5.43	5.07	5.16	4	4	5.27	4.71	4.19
	SW-620	5.97	5.48	4.97	5.48	4	4	5.38	4.67	4.07
	*Panel average*	6.00	5.48	4.91	5.31	4.25	4	5.32	4.75	4.20
**CNS Cancer**	SF-268	5.6	5.07	4	5.11	4	4	5.15	4.44	4
	SF-295	5.52	4.51	4	5.53	4.75	4	5.10	4.68	4.32
	SF-539	5.67	5.29	4.22	5.57	5.03	4.09	5.55	5.05	4.48
	SNB-19	5.51	4.63	4	5.39	4.04	4	5.05	4.61	4.20
	SNB-75	5.6	4.47	4	5.38	4.34	4	5.17	4.74	4.35
	U251	5.81	5.44	5.07	5.42	4.73	4	5.33	4.78	4.31
	*Panel average*	5.62	4.90	4.22	5.40	4.48	4.02	5.22	4.72	4.28
**Melanoma**	LOX IMVI	5.84	5.49	5.15	5.51	4.54	4	5.57	5.07	4
	MALME-3M	5.24	4.1	4	5.21	4	4	4.85	4.56	4.27
	M14	5.77	5.36	4.24	5.51	4.58	4	5.42	4.80	4.35
	MDA-MB-435	5.94	5.53	5.11	6.03	5.41	4.19	5.53	4.92	4.40
	SK-MEL-2	4.51	4	4	5.54	4.73	4	4.78	4.39	4.06
	SK-MEL-28	5.74	5.4	NT	5.3	4	4	5.35	4.80	4.30
	SK-MEL-5	5.67	5.21	4	5.66	4.99	4	5.06	4.65	4.28
	UACC-257	5.61	5.13	4	4.97	4	4	4.94	4.62	4.31
	UACC-62	5.72	5.31	4.52	5.62	5	4	5.19	4.69	4.26
	*Panel average*	5.56	5.06	4.38	5.48	4.58	4.02	5.19	4.72	4.25
**Ovarian Cancer**	IGROV-1	5.57	NT	4	5.37	4	4	5.10	4.57	4.09
	OVCAR-3	5.55	5	4	5.3	4.09	4	5.18	4.61	4.17
	OVCAR-4	5.39	4	4	5.04	4	4	5.03	4.44	4
	OVCAR-5	5.6	5.09	4	5.31	4.27	4	4.78	4.45	4.12
	OVCAR-8	5.53	4	4	5.06	4	4	5.13	4.55	4.08
	NCI/ADR-RES	5.38	4	4	5.45	4	4	5.14	4.12	4
	SK-OV-3	4.44	4	4	5.06	4.03	4	5.05	4.68	4.33
	*Panel average*	5.35	4.35	4.00	5.23	4.06	4	5.06	4.49	4.11
**Renal Cancer**	786-0	5.64	5.1	4	5.25	4	4	5.48	4.97	4.42
	A-498	5.65	4.7	4	6.62	5.04	4	4.80	4.48	4.16
	ACHN	5.69	5.27	4	4.86	4	4	4.91	4.54	4.17
	CAKI-1	5.5	4	4	4.94	4	4	4.92	4.60	4.30
	RXF-393	5.83	5.52	5.2	5.1	4.11	4	5.52	4.95	4.27
	SN12C	5.51	4.75	4	5.26	4	4	5.08	4.60	4.20
	TK-10	5.42	4.6	4	5.42	4.56	4	4.85	4.51	4.18
	UO-31	5.79	5.47	5.14	5.32	4	4	4.95	4.61	4.27
	*Panel average*	5.63	4.93	4.29	5.35	4.21	4	5.06	4.66	4.25
**Prostate Cancer**	PC-3	5.48	4	4	5.39	4	4	5.06	4.59	4.15
	DU-145	5.51	4.85	4	4.82	4	4	4.81	4.53	4.25
	*Panel average*	5.50	4.43	4.00	5.11	4.00	4	4.93	4.56	4.20
**Breast Cancer**	MCF7	6.29	5.02	4	5.46	4	4	5.48	4.46	4
	MDA-MB-231/ATCC	5.65	5.22	4	5.44	4.36	4	4.75	4.25	4
	HS 578T	5.57	4	4	5.43	4.32	4	4.96	4.23	4
	BT-549	5.74	5.35	4.66	5.54	4.45	4	5.30	4.86	4.37
	T-47D	5.65	4	4	5.46	4.09	4	5.08	4.33	4
	MDA-MB-468	5.81	5.43	4	5.54	4.56	4	NT	NT	NT
	*Panel average*	5.79	4.84	4.11	5.48	4.30	4	5.11	4.43	4.07
**Overall average**		5.59	4.81	4.24	5.37	4.28	4.01	5.16	4.63	4.17
**Range**		4–6.5	4–5.88	4–5.39	4.71–6.62	4–5.41	4–4.19	4.73–5.68	4–5.20	4–4.53

^1^ NT = not tested against the cell line.

## Data Availability

Not applicable.

## Supplementary Materials

The following supporting information can be downloaded at: https://www.mdpi.com/article/10.3390/ijms232214374/s1.
