# Supplementary material for: Antiproliferative Activity Predictor: A New Reliable In Silico Tool for Drug Response Prediction against NCI60 Panel
Source: Int J Mol Sci. 2022 Nov 19;23(22):14374. doi: 10.3390/ijms232214374 (PMC9694168; doi:10.3390/ijms232214374)
Supplement: Supplementary file 1 [file ijms-23-14374-s001.zip › S1.pdf]

MOL_DES_ID	name
1000000	Total number of atoms
2000000	Number of C atoms
3000000	Number of H atoms
4000000	Number of O atoms
5000000	Number of N atoms
6000000	Number of S atoms
7000000	Number of F atoms
8000000	Number of Cl atoms
9000000	Number of Br atoms
10000000	Number of I atoms
11000000	Number of P atoms
12000000	Number of other atoms
13000000	Relative number of C atoms
14000000	Relative number of H atoms
15000000	Relative number of O atoms
16000000	Relative number of N atoms
17000000	Relative number of S atoms
18000000	Relative number of F atoms
19000000	Relative number of Cl atoms
20000000	Relative number of Br atoms
21000000	Relative number of I atoms
22000000	Relative number of P atoms
23000000	Relative number of others atoms
24000000	Total number of bonds
25000000	Number of single bonds
26000000	Relative number of single bonds
27000000	Number of double bonds
28000000	Relative number of double bonds

29000000	Number of triple bonds
30000000	Relative number of triple bonds
31000000	Number of aromatic bonds
32000000	Relative number of aromatic bonds
33000000	Number of Aromatic Atoms
34000000	Relative Number of Aromatic Atoms
35000000	Number of rings
35000000	Number of rings
36000000	Relative number of rings
36000000	Relative number of rings
37000000	Number of benzene rings
38000000	Molecular weight
39000000	Average atom weight
40000000	Wiener index
41000000	weiner polarity number
42000000	Randic index (order 0)
43000000	Kier&Hall index (order 0)
44000000	Randic index (order 1)
45000000	Randic index (order 2)
46000000	Randic index (order 3)
47000000	Kier&Hall index (order 1)
48000000	Kier&Hall index (order 2)
49000000	Kier&Hall index (order 3)
50000000	Information content (order 0)
51000000	Average Information content (order 0)
52000000	Structural Information content (order 0)
53000000	Average Structural Information content (order 0)
54000000	Complementary Information content (order 0)
55000000	Average Complementary Information content (order 0)

56000000	Bonding Information content (order 0)
57000000	Average Bonding Information content (order 0)
58000000	Average Information content (order 1)
59000000	Information content (order 1)
60000000	Average Structural Information content (order 1)
61000000	Structural Information content (order 1)
62000000	Average Complementary Information content (order 1)
63000000	Complementary Information content (order 1)
64000000	Bonding Information content (order 1)
65000000	Average Bonding Information content (order 1)
66000000	Average Information content (order 2)
67000000	Information content (order 2)
68000000	Average Structural Information content (order 2)
69000000	Structural Information content (order 2)
70000000	Average Complementary Information content (order 2)
71000000	Complementary Information content (order 2)
72000000	Bonding Information content (order 2)
73000000	Average Bonding Information content (order 2)
74000000	AIC3
75000000	IC3
76000000	ASIC3
77000000	SIC3
78000000	ACIC3
79000000	CIC3
80000000	BIC3
81000000	ABIC3
82000000	Balaban index
83000000	Kier shape index (order 1)
84000000	Kier shape index (order 2)

85000000	Kier shape index (order 3)
86000000	Kier flexibility index
87000000	Gravitation index (all bonds)
88000000	Gravitational Index Heavy atoms
89000000	SQRGravitational Index Heavy atoms
90000000	CRGravitational Index Heavy atoms
91000000	SQRGravitational Index
92000000	CRGravitational Index
93000000	Gravitational Index all pairs
94000000	Gravitational Index all pairs2
95000000	Gravitational Index all pairs3
96000000	Momento di inerzia A-codessastyle
97000000	Momento di inerzia B-codessastyle
98000000	Momento di inerzia C-codessastyle
99000000	Moments of inertia A
100000000	Moments of inertia B
101000000	Moments of inertia C
109000000	Molecular volume
110000000	Acidic Group
111000000	Sum atoms polarizzabilities
112000000	AutoCorrelationCharge1
113000000	AutoCorrelationCharge2
114000000	AutoCorrelationCharge3
115000000	AutoCorrelationCharge4
116000000	AutoCorrelationCharge5
117000000	AutoCorrelationMass1
118000000	AutoCorrelationMass2
119000000	AutoCorrelationMass3
120000000	AutoCorrelationMass4

121000000	AutoCorrelationMass5
122000000	AutoCorrelationPolarizzability1
123000000	AutoCorrelationPolarizzability2
124000000	AutoCorrelationPolarizzability3
125000000	AutoCorrelationPolarizzability4
126000000	AutoCorrelationPolarizzability5
127000000	Basic Group
128000000	nhigh lowest atom weighted BCUTS
129000000	nlow highest atom weighted BCUTS
130000000	nhigh lowest partial charge weighted BCUTS
131000000	nlow highest partial charge weighted BCUTS
132000000	nhigh lowest polarizability weighted BCUTS
133000000	nlow highest polarizability weighted BCUTS
134000000	BPOL
135000000	CarbonType-C1SP1
136000000	CarbonType-C2SP1
137000000	CarbonType-C1SP2
138000000	CarbonType-C2SP2
139000000	CarbonType-C3SP2
140000000	CarbonType-C1SP3
141000000	CarbonType-C2SP3
142000000	CarbonType-C3SP3
143000000	CarbonType-C4SP3
144000000	PPSA1 Partial positive surface area (Zefirov PC)
145000000	PPSA2 Total charge weighted PPSA (Zefirov PC)
146000000	PPSA3 Atomic charge weighted PPSA (Zefirov PC)
147000000	PNSA1 Partial negative surface area (Zefirov PC)
148000000	PNSA2 Total charge weighted PNSA (Zefirov PC)
149000000	PNSA3 Atomic charge weighted PNSA (Zefirov PC)

150000000	DPSA1 Difference in CPSAs (PPSA1-PNSA1) (Zefirov PC)
151000000	DPSA2 Difference in CPSAs (PPSA2-PNSA2) (Zefirov PC)
152000000	DPSA3 Difference in CPSAs (PPSA3-PNSA3) (Zefirov PC)
153000000	FPSA1 Fractional PPSA (PPSA-1/TMSA) (Zefirov PC)
154000000	FPSA2 Fractional PPSA (PPSA-2/TMSA) (Zefirov PC)
155000000	FPSA3 Fractional PPSA (PPSA-3/TMSA) (Zefirov PC)
156000000	FNSA1 Fractional PNSA (PNSA-1/TMSA) (Zefirov PC)
157000000	FNSA2 Fractional PNSA (PNSA-2/TMSA) (Zefirov PC)
158000000	FNSA3 Fractional PNSA (PNSA-3/TMSA) (Zefirov PC)
159000000	WPSA1 Weighted PPSA (PPSA1*TMSA/1000) (Zefirov PC)
160000000	WPSA2 Weighted PPSA (PPSA2*TMSA/1000) (Zefirov PC)
161000000	WPSA3 Weighted PPSA (PPSA3*TMSA/1000) (Zefirov PC)
162000000	WNSA1 Weighted PNSA (PNSA1*TMSA/1000) (Zefirov PC)
163000000	WNSA2 Weighted PNSA (PNSA2*TMSA/1000) (Zefirov PC)
164000000	WNSA3 Weighted PNSA (PNSA3*TMSA/1000) (Zefirov PC)
165000000	RPCG Relative positive charge (QMPOS/QTPLUS) (Zefirov PC)
166000000	RPCS Relative positive charged SA (SAMPOS*RPCG) (Zefirov PC)
166000000	RPCS Relative positive charged SA (SAMPOS*RPCG) (Zefirov PC)
167000000	RNCS Relative negative charged SA (SAMNEG*RNCG) (Zefirov PC)
168000000	THSA
169000000	TPSA
170000000	RHSA
171000000	RPSA
172000000	Max partial charge (Zefirov) for atoms for atom H
173000000	Max partial charge (Zefirov) for atoms for atom C
174000000	Min partial charge (Zefirov) for atoms for atom H
175000000	Min partial charge (Zefirov) for atoms for atom C
176000000	Max partial charge (Zefirov) for all atom types
177000000	Min partial charge (Zefirov) for all atom types

178000000	Polarity parameter (Zefirov)
179000000	HA dependent HDSA-1 (Zefirov PC)
180000000	HA dependent HDSA-1/TMSA (Zefirov PC)
181000000	HASA-1 (Zefirov PC)
182000000	HASA-1/TMSA (Zefirov PC)
183000000	HA dependent HDSA-2 (Zefirov PC)
184000000	HA dependent HDSA-2/TMSA (Zefirov PC)
185000000	HA dependent HDSA-2/SQRT(TMSA) (Zefirov PC)
186000000	HA dependent HDCA-1 (Zefirov PC)
187000000	HA dependent HDCA-1/TMSA (Zefirov PC)
188000000	HA dependent HDCA-2 (Zefirov PC)
189000000	HA dependent HDCA-2/TMSA (Zefirov PC)
190000000	HA dependent HDCA-2/SQRT(TMSA) (Zefirov PC)
191000000	HASA-2 (Zefirov PC)
192000000	HASA-2/TMSA (Zefirov PC)
193000000	HASA-2/SQRT(TMSA) (Zefirov PC)
194000000	HACA-1 (Zefirov PC)
195000000	HACA-1/TMSA (Zefirov PC)
196000000	HACA-2 (Zefirov PC)
197000000	HACA-2/TMSA (Zefirov PC)
198000000	HACA-2/SQRT(TMSA) (Zefirov PC)
199000000	Tot point-charge comp. of the molecular dipole
200000000	Tot hybridization comp. of the molecular dipole
201000000	Tot dipole of the molecule
202000000	Tot molecular 1-center E-N attraction
203000000	Tot molecular 1-center E-E repulsion
204000000	Tot molecular 2-center resonance energy
205000000	Tot molecular 2-center exchange energy
206000000	EXCHANGE + RESONANCE ENERGY

207000000	ELECTRON-ELECTRON REPULSION
208000000	ELECTRON-NUCLEAR ATTRACTION
209000000	NUCLEAR-NUCLEAR REPULSION
210000000	TOTAL ELECTROSTATIC INTERACTION
211000000	GRAND TOTAL OF TWO-CENTER TERMS
212000000	ETOT (EONE + ETWO)
213000000	Principal moment of inertia A
214000000	Relative principal moment of inertia A
215000000	Principal moment of inertia B
216000000	Relative principal moment of inertia B
217000000	Principal moment of inertia C
218000000	Relative principal moment of inertia C
219000000	Principal moment of inertia A IN UNITS OF 10**(-40)*GRAM-CM**2
220000000	Relative moment of inertia A IN UNITS OF 10**(-40)*GRAM-CM**2
221000000	Principal moment of inertia B IN UNITS OF 10**(-40)*GRAM-CM**2
222000000	Relative moment of inertia B IN UNITS OF 10**(-40)*GRAM-CM**2
223000000	Principal moment of inertia C IN UNITS OF 10**(-40)*GRAM-CM**2
224000000	Relative moment of inertia C IN UNITS OF 10**(-40)*GRAM-CM**2
225000000	ENERGY OF "REORIENTED" SYSTEM WITHOUT FIELD
226000000	PPSA-1 Partial positive surface area (MOPAC PC)
227000000	PPSA-2 Total charge weighted PPSA (MOPAC PC)
228000000	PPSA-3 Atomic charge weighted PPSA (MOPAC PC)
229000000	PNSA-1 Partial negative surface area (MOPAC PC)
230000000	PNSA-2 Total charge weighted PNSA (MOPAC PC)
231000000	PNSA-3 Atomic charge weighted PNSA (MOPAC PC)
232000000	DPSA-1 Difference in CPSAs (PPSA1-PNSA1) (MOPAC PC)
233000000	DPSA-2 Difference in CPSAs (PPSA2-PNSA2) (MOPAC PC)
234000000	DPSA-3 Difference in CPSAs (PPSA3-PNSA3) (MOPAC PC)
235000000	FPSA-1 Fractional PPSA (PPSA-1/TMSA) (MOPAC PC)

236000000	FPSA-2 Fractional PPSA (PPSA-2/TMSA) (MOPAC PC)
237000000	FPSA-3 Fractional PPSA (PPSA-3/TMSA) (MOPAC PC)
238000000	FNSA-1 Fractional PNSA (PNSA-1/TMSA) (MOPAC PC)
239000000	FNSA-2 Fractional PNSA (PNSA-2/TMSA) (MOPAC PC)
240000000	FNSA-3 Fractional PNSA (PNSA-3/TMSA) (MOPAC PC)
241000000	WPSA-1 Weighted PPSA (PPSA1*TMSA/1000) (MOPAC PC)
242000000	WPSA-2 Weighted PPSA (PPSA2*TMSA/1000) (MOPAC PC)
243000000	WPSA-3 Weighted PPSA (PPSA3*TMSA/1000) (MOPAC PC)
244000000	WNSA-1 Weighted PNSA (PNSA1*TMSA/1000) (MOPAC PC)
245000000	WNSA-2 Weighted PNSA (PNSA2*TMSA/1000) (MOPAC PC)
246000000	WNSA-3 Weighted PNSA (PNSA3*TMSA/1000) (MOPAC PC)
247000000	RPCG Relative positive charge (QMPOS/QTPLUS) (MOPAC PC)
248000000	RNCG Relative negative charge (QMNEG/QTMINUS) (MOPAC PC)
249000000	RPCS Relative positive charged SA (SAMPOS*RPCG) (MOPAC PC)
250000000	RNCS Relative negative charged SA (SAMNEG*RNCG) (MOPAC PC)
251000000	THSA Mopac
252000000	TPSA Mopac
253000000	RHSA Mopac
254000000	RPSA Mopac
255000000	Max H partial charge MOPAC
256000000	Max C partial charge MOPAC
257000000	Min H partial charge MOPAC
258000000	Min C partial charge MOPAC
259000000	Max ALL partial charge MOPAC
260000000	Min ALL partial charge MOPAC
261000000	Polarity Parameter (MOPAC)
262000000	HDSA H-donors surface area (MOPAC PC)
263000000	FHDSA Fractional HDSA (HDSA/TMSA) (MOPAC PC)
264000000	HASA H-acceptors surface area (MOPAC PC)

265000000	FHASA Fractional HASA (HASA/TMSA) (MOPAC PC)
266000000	HA dependent HDSA-2 (MOPAC PC)
267000000	HA dependent HDSA-2/TMSA (MOPAC PC)
268000000	HA dependent HDSA-2/SQRT(TMSA) (MOPAC PC)
269000000	HA dependent HDCA-1 (MOPAC PC)
270000000	HA dependent HDCA-1/TMSA (MOPAC PC)
271000000	HA dependent HDCA-2 (MOPAC PC)
272000000	HA dependent HDCA-2/TMSA (MOPAC PC)
273000000	HA dependent HDCA-2/SQRT(TMSA) (MOPAC PC)
274000000	HASA-2 (MOPAC PC)
275000000	HASA-2/TMSA (MOPAC PC)
276000000	HASA-2/SQRT(TMSA) (MOPAC PC)
277000000	HACA-1 (MOPAC PC)
278000000	HACA-1/TMSA (MOPAC PC)
279000000	HACA-2 (MOPAC PC)
280000000	HACA-2/TMSA (MOPAC PC)
281000000	HACA-2/SQRT(TMSA) (MOPAC PC)
282000000	Min atomic orbital electronic population
283000000	Max atomic orbital electronic population
284000000	Max SIGMA-SIGMA bond order
285000000	Max SIGMA-PI bond order
286000000	Max PI-PI bond order
287000000	Max bonding contribution of one MO
288000000	Max antibonding contribution of one MO
289000000	Avg valency for atom Cl
290000000	Avg bond order for atom Cl
291000000	Min valency for atom N
292000000	Max valency for atom N
293000000	Avg valency for atom N

294000000	Min (>0.1) bond order for atom N
295000000	Max bond order for atom N
296000000	Avg bond order for atom N
297000000	Min valency for atom C
298000000	Max valency for atom C
299000000	Avg valency for atom C
300000000	Min (>0.1) bond order for atom C
301000000	Max bond order for atom C
302000000	Avg bond order for atom C
303000000	Min valency for atom O
304000000	Max valency for atom O
305000000	Avg valency for atom O
306000000	Min (>0.1) bond order for atom O
307000000	Max bond order for atom O
308000000	Avg bond order for atom O
309000000	Min valency for atom H
310000000	Max valency for atom H
311000000	Avg valency for atom H
312000000	Min (>0.1) bond order for atom H
313000000	Max bond order for atom H
314000000	Avg bond order for atom H
315000000	Min e-e repulsion for atom N
316000000	Max e-e repulsion for atom N
317000000	Min e-n attraction for atom N
318000000	Max e-n attraction for atom N
319000000	Min atomic state energy for atom N
320000000	Max atomic state energy for atom N
321000000	Min e-e repulsion for atom C
322000000	Max e-e repulsion for atom C

323000000	Min e-n attraction for atom C
324000000	Max e-n attraction for atom C
325000000	Min atomic state energy for atom C
326000000	Max atomic state energy for atom C
327000000	Min e-e repulsion for atom O
328000000	Max e-e repulsion for atom O
329000000	Min e-n attraction for atom O
330000000	Max e-n attraction for atom O
331000000	Min atomic state energy for atom O
332000000	Max atomic state energy for atom O
333000000	Min e-e repulsion for atom H
334000000	Max e-e repulsion for atom H
335000000	Min e-n attraction for atom H
336000000	Max e-n attraction for atom H
337000000	Min atomic state energy for atom H
338000000	Max atomic state energy for atom H
339000000	Min resonance energy for bond C - N
340000000	Max resonance energy for bond C - N
341000000	Min exchange energy for bond C - N
342000000	Max exchange energy for bond C - N
343000000	Min e-e repulsion for bond C - N
344000000	Max e-e repulsion for bond C - N
345000000	Min e-n attraction for bond C - N
346000000	Max e-n attraction for bond C - N
347000000	Min n-n repulsion for bond C - N
348000000	Max n-n repulsion for bond C - N
349000000	Min coulombic interaction for bond C - N
350000000	Max coulombic interaction for bond C - N
351000000	Min total interaction for bond C - N

352000000	Max total interaction for bond C - N
353000000	Min resonance energy for interaction C - N in 2 bonds path
354000000	Max resonance energy for interaction C - N in 2 bonds path
355000000	Min exchange energy for interaction C - N in 2 bonds path
356000000	Max exchange energy for interaction C - N in 2 bonds path
357000000	Min e-e repulsion for interaction C - N in 2 bonds path
358000000	Max e-e repulsion for interaction C - N in 2 bonds path
359000000	Min e-n attraction for interaction C - N in 2 bonds path
360000000	Max e-n attraction for interaction C - N in 2 bonds path
361000000	Min n-n repulsion for interaction C - N in 2 bonds path
362000000	Max n-n repulsion for interaction C - N in 2 bonds path
363000000	Min coulumbic interaction for interaction C - N in 2 bonds path
364000000	Max coulumbic interaction for interaction C - N in 2 bonds path
365000000	Min total interaction for interaction C - N in 2 bonds path
366000000	Max total interaction for interaction C - N in 2 bonds path
367000000	Min resonance energy for interaction C - N in 3 bonds path
368000000	Max resonance energy for interaction C - N in 3 bonds path
369000000	Min exchange energy for interaction C - N in 3 bonds path
370000000	Max exchange energy for interaction C - N in 3 bonds path
371000000	Min e-e repulsion for interaction C - N in 3 bonds path
372000000	Max e-e repulsion for interaction C - N in 3 bonds path
373000000	Min e-n attraction for interaction C - N in 3 bonds path
374000000	Max e-n attraction for interaction C - N in 3 bonds path
375000000	Min n-n repulsion for interaction C - N in 3 bonds path
376000000	Max n-n repulsion for interaction C - N in 3 bonds path
377000000	Min coulumbic interaction for interaction C - N in 3 bonds path
378000000	Max coulumbic interaction for interaction C - N in 3 bonds path
379000000	Min total interaction for interaction C - N in 3 bonds path
380000000	Max total interaction for interaction C - N in 3 bonds path

381000000	Min resonance energy for interaction C - N in > 3 bonds path
382000000	Max resonance energy for interaction C - N in > 3 bonds path
383000000	Min exchange energy for interaction C - N in > 3 bonds path
384000000	Max exchange energy for interaction C - N in > 3 bonds path
385000000	Min e-e repulsion for interaction C - N in > 3 bonds path
386000000	Max e-e repulsion for interaction C - N in > 3 bonds path
387000000	Min e-n attraction for interaction C - N in > 3 bonds path
388000000	Max e-n attraction for interaction C - N in > 3 bonds path
389000000	Min n-n repulsion for interaction C - N in > 3 bonds path
390000000	Max n-n repulsion for interaction C - N in > 3 bonds path
391000000	Min coulumbic interaction for interaction C - N in > 3 bonds path
392000000	Max coulumbic interaction for interaction C - N in > 3 bonds path
393000000	Min total interaction for interaction C - N in > 3 bonds path
394000000	Max total interaction for interaction C - N in > 3 bonds path
395000000	Min resonance energy for bond C - C
396000000	Max resonance energy for bond C - C
397000000	Min exchange energy for bond C - C
398000000	Max exchange energy for bond C - C
399000000	Min e-e repulsion for bond C - C
400000000	Max e-e repulsion for bond C - C
401000000	Min e-n attraction for bond C - C
402000000	Max e-n attraction for bond C - C
403000000	Min n-n repulsion for bond C - C
404000000	Max n-n repulsion for bond C - C
405000000	Min coulombic interaction for bond C - C
406000000	Max coulombic interaction for bond C - C
407000000	Min total interaction for bond C - C
408000000	Max total interaction for bond C - C
409000000	Min resonance energy for interaction C - C in 2 bonds path

410000000	Max resonance energy for interaction C - C in 2 bonds path
411000000	Min exchange energy for interaction C - C in 2 bonds path
412000000	Max exchange energy for interaction C - C in 2 bonds path
413000000	Min e-e repulsion for interaction C - C in 2 bonds path
414000000	Max e-e repulsion for interaction C - C in 2 bonds path
415000000	Min e-n attraction for interaction C - C in 2 bonds path
416000000	Max e-n attraction for interaction C - C in 2 bonds path
417000000	Min n-n repulsion for interaction C - C in 2 bonds path
418000000	Max n-n repulsion for interaction C - C in 2 bonds path
419000000	Min coulumbic interaction for interaction C - C in 2 bonds path
420000000	Max coulumbic interaction for interaction C - C in 2 bonds path
421000000	Min total interaction for interaction C - C in 2 bonds path
422000000	Max total interaction for interaction C - C in 2 bonds path
423000000	Min resonance energy for interaction C - C in 3 bonds path
424000000	Max resonance energy for interaction C - C in 3 bonds path
425000000	Min exchange energy for interaction C - C in 3 bonds path
426000000	Max exchange energy for interaction C - C in 3 bonds path
427000000	Min e-e repulsion for interaction C - C in 3 bonds path
428000000	Max e-e repulsion for interaction C - C in 3 bonds path
429000000	Min e-n attraction for interaction C - C in 3 bonds path
430000000	Max e-n attraction for interaction C - C in 3 bonds path
431000000	Min n-n repulsion for interaction C - C in 3 bonds path
432000000	Max n-n repulsion for interaction C - C in 3 bonds path
433000000	Min coulumbic interaction for interaction C - C in 3 bonds path
434000000	Max coulumbic interaction for interaction C - C in 3 bonds path
435000000	Min total interaction for interaction C - C in 3 bonds path
436000000	Max total interaction for interaction C - C in 3 bonds path
437000000	Min resonance energy for interaction C - C in > 3 bonds path
438000000	Max resonance energy for interaction C - C in > 3 bonds path

439000000	Min exchange energy for interaction C - C in > 3 bonds path
440000000	Max exchange energy for interaction C - C in > 3 bonds path
441000000	Min e-e repulsion for interaction C - C in > 3 bonds path
442000000	Max e-e repulsion for interaction C - C in > 3 bonds path
443000000	Min e-n attraction for interaction C - C in > 3 bonds path
444000000	Max e-n attraction for interaction C - C in > 3 bonds path
445000000	Min n-n repulsion for interaction C - C in > 3 bonds path
446000000	Max n-n repulsion for interaction C - C in > 3 bonds path
447000000	Min coulumbic interaction for interaction C - C in > 3 bonds path
448000000	Max coulumbic interaction for interaction C - C in > 3 bonds path
449000000	Min total interaction for interaction C - C in > 3 bonds path
450000000	Max total interaction for interaction C - C in > 3 bonds path
451000000	Min resonance energy for bond C - O
452000000	Max resonance energy for bond C - O
453000000	Min exchange energy for bond C - O
454000000	Max exchange energy for bond C - O
455000000	Min e-e repulsion for bond C - O
456000000	Max e-e repulsion for bond C - O
457000000	Min e-n attraction for bond C - O
458000000	Max e-n attraction for bond C - O
459000000	Min n-n repulsion for bond C - O
460000000	Max n-n repulsion for bond C - O
461000000	Min coulombic interaction for bond C - O
462000000	Max coulombic interaction for bond C - O
463000000	Min total interaction for bond C - O
464000000	Max total interaction for bond C - O
465000000	Min resonance energy for interaction C - O in 2 bonds path
466000000	Max resonance energy for interaction C - O in 2 bonds path
467000000	Min exchange energy for interaction C - O in 2 bonds path

468000000	Max exchange energy for interaction C - O in 2 bonds path
469000000	Min e-e repulsion for interaction C - O in 2 bonds path
470000000	Max e-e repulsion for interaction C - O in 2 bonds path
471000000	Min e-n attraction for interaction C - O in 2 bonds path
472000000	Max e-n attraction for interaction C - O in 2 bonds path
473000000	Min n-n repulsion for interaction C - O in 2 bonds path
474000000	Max n-n repulsion for interaction C - O in 2 bonds path
475000000	Min coulumbic interaction for interaction C - O in 2 bonds path
476000000	Max coulumbic interaction for interaction C - O in 2 bonds path
477000000	Min total interaction for interaction C - O in 2 bonds path
478000000	Max total interaction for interaction C - O in 2 bonds path
479000000	Min resonance energy for interaction C - O in 3 bonds path
480000000	Max resonance energy for interaction C - O in 3 bonds path
481000000	Min exchange energy for interaction C - O in 3 bonds path
482000000	Max exchange energy for interaction C - O in 3 bonds path
483000000	Min e-e repulsion for interaction C - O in 3 bonds path
484000000	Max e-e repulsion for interaction C - O in 3 bonds path
485000000	Min e-n attraction for interaction C - O in 3 bonds path
486000000	Max e-n attraction for interaction C - O in 3 bonds path
487000000	Min n-n repulsion for interaction C - O in 3 bonds path
488000000	Max n-n repulsion for interaction C - O in 3 bonds path
489000000	Min coulumbic interaction for interaction C - O in 3 bonds path
490000000	Max coulumbic interaction for interaction C - O in 3 bonds path
491000000	Min total interaction for interaction C - O in 3 bonds path
492000000	Max total interaction for interaction C - O in 3 bonds path
493000000	Min resonance energy for interaction C - O in > 3 bonds path
494000000	Max resonance energy for interaction C - O in > 3 bonds path
495000000	Min exchange energy for interaction C - O in > 3 bonds path
496000000	Max exchange energy for interaction C - O in > 3 bonds path

497000000	Min e-e repulsion for interaction C - O in > 3 bonds path
498000000	Max e-e repulsion for interaction C - O in > 3 bonds path
499000000	Min e-n attraction for interaction C - O in > 3 bonds path
500000000	Max e-n attraction for interaction C - O in > 3 bonds path
501000000	Min n-n repulsion for interaction C - O in > 3 bonds path
502000000	Max n-n repulsion for interaction C - O in > 3 bonds path
503000000	Min coulumbic interaction for interaction C - O in > 3 bonds path
504000000	Max coulumbic interaction for interaction C - O in > 3 bonds path
505000000	Min total interaction for interaction C - O in > 3 bonds path
506000000	Max total interaction for interaction C - O in > 3 bonds path
507000000	Min resonance energy for bond H - C
508000000	Max resonance energy for bond H - C
509000000	Min exchange energy for bond H - C
510000000	Max exchange energy for bond H - C
511000000	Min e-e repulsion for bond H - C
512000000	Max e-e repulsion for bond H - C
513000000	Min e-n attraction for bond H - C
514000000	Max e-n attraction for bond H - C
515000000	Min n-n repulsion for bond H - C
516000000	Max n-n repulsion for bond H - C
517000000	Min coulombic interaction for bond H - C
518000000	Max coulombic interaction for bond H - C
519000000	Min total interaction for bond H - C
520000000	Max total interaction for bond H - C
521000000	Min resonance energy for interaction C - H in 2 bonds path
522000000	Max resonance energy for interaction C - H in 2 bonds path
523000000	Min exchange energy for interaction C - H in 2 bonds path
524000000	Max exchange energy for interaction C - H in 2 bonds path
525000000	Min e-e repulsion for interaction C - H in 2 bonds path

526000000	Max e-e repulsion for interaction C - H in 2 bonds path
527000000	Min e-n attraction for interaction C - H in 2 bonds path
528000000	Max e-n attraction for interaction C - H in 2 bonds path
529000000	Min n-n repulsion for interaction C - H in 2 bonds path
530000000	Max n-n repulsion for interaction C - H in 2 bonds path
531000000	Min coulumbic interaction for interaction C - H in 2 bonds path
532000000	Max coulumbic interaction for interaction C - H in 2 bonds path
533000000	Min total interaction for interaction C - H in 2 bonds path
534000000	Max total interaction for interaction C - H in 2 bonds path
535000000	Min resonance energy for interaction C - H in 3 bonds path
536000000	Max resonance energy for interaction C - H in 3 bonds path
537000000	Min exchange energy for interaction C - H in 3 bonds path
538000000	Max exchange energy for interaction C - H in 3 bonds path
539000000	Min e-e repulsion for interaction C - H in 3 bonds path
540000000	Max e-e repulsion for interaction C - H in 3 bonds path
541000000	Min e-n attraction for interaction C - H in 3 bonds path
542000000	Max e-n attraction for interaction C - H in 3 bonds path
543000000	Min n-n repulsion for interaction C - H in 3 bonds path
544000000	Max n-n repulsion for interaction C - H in 3 bonds path
545000000	Min coulumbic interaction for interaction C - H in 3 bonds path
546000000	Max coulumbic interaction for interaction C - H in 3 bonds path
547000000	Min total interaction for interaction C - H in 3 bonds path
548000000	Max total interaction for interaction C - H in 3 bonds path
549000000	Min resonance energy for interaction C - H in > 3 bonds path
550000000	Max resonance energy for interaction C - H in > 3 bonds path
551000000	Min exchange energy for interaction C - H in > 3 bonds path
552000000	Max exchange energy for interaction C - H in > 3 bonds path
553000000	Min e-e repulsion for interaction C - H in > 3 bonds path
554000000	Max e-e repulsion for interaction C - H in > 3 bonds path

555000000 Min e-n attraction for interaction C - H in > 3 bonds path
556000000 Max e-n attraction for interaction C - H in > 3 bonds path
557000000 Min n-n repulsion for interaction C - H in > 3 bonds path
558000000 Max n-n repulsion for interaction C - H in > 3 bonds path
559000000 Min coulumbic interaction for interaction C - H in > 3 bonds path
560000000 Max coulumbic interaction for interaction C - H in > 3 bonds path
561000000 Min total interaction for interaction C - H in > 3 bonds path
562000000 Max total interaction for interaction C - H in > 3 bonds path
563000000 Min resonance energy for interaction N - H in 2 bonds path
564000000 Max resonance energy for interaction N - H in 2 bonds path
565000000 Min exchange energy for interaction N - H in 2 bonds path
566000000 Max exchange energy for interaction N - H in 2 bonds path
567000000 Min e-e repulsion for interaction N - H in 2 bonds path
568000000 Max e-e repulsion for interaction N - H in 2 bonds path
569000000 Min e-n attraction for interaction N - H in 2 bonds path
570000000 Max e-n attraction for interaction N - H in 2 bonds path
571000000 Min n-n repulsion for interaction N - H in 2 bonds path
572000000 Max n-n repulsion for interaction N - H in 2 bonds path
573000000 Min coulumbic interaction for interaction N - H in 2 bonds path
574000000 Max coulumbic interaction for interaction N - H in 2 bonds path
575000000 Min total interaction for interaction N - H in 2 bonds path
576000000 Max total interaction for interaction N - H in 2 bonds path
577000000 Min resonance energy for interaction N - H in 3 bonds path
578000000 Max resonance energy for interaction N - H in 3 bonds path
579000000 Min exchange energy for interaction N - H in 3 bonds path
580000000 Max exchange energy for interaction N - H in 3 bonds path
581000000 Min e-e repulsion for interaction N - H in 3 bonds path
582000000 Max e-e repulsion for interaction N - H in 3 bonds path
583000000 Min e-n attraction for interaction N - H in 3 bonds path

584000000	Max e-n attraction for interaction N - H in 3 bonds path
585000000	Min n-n repulsion for interaction N - H in 3 bonds path
586000000	Max n-n repulsion for interaction N - H in 3 bonds path
587000000	Min coulumbic interaction for interaction N - H in 3 bonds path
588000000	Max coulumbic interaction for interaction N - H in 3 bonds path
589000000	Min total interaction for interaction N - H in 3 bonds path
590000000	Max total interaction for interaction N - H in 3 bonds path
591000000	Min resonance energy for interaction N - H in > 3 bonds path
592000000	Max resonance energy for interaction N - H in > 3 bonds path
593000000	Min exchange energy for interaction N - H in > 3 bonds path
594000000	Max exchange energy for interaction N - H in > 3 bonds path
595000000	Min e-e repulsion for interaction N - H in > 3 bonds path
596000000	Max e-e repulsion for interaction N - H in > 3 bonds path
597000000	Min e-n attraction for interaction N - H in > 3 bonds path
598000000	Max e-n attraction for interaction N - H in > 3 bonds path
599000000	Min n-n repulsion for interaction N - H in > 3 bonds path
600000000	Max n-n repulsion for interaction N - H in > 3 bonds path
601000000	Min coulumbic interaction for interaction N - H in > 3 bonds path
602000000	Max coulumbic interaction for interaction N - H in > 3 bonds path
603000000	Min total interaction for interaction N - H in > 3 bonds path
604000000	Max total interaction for interaction N - H in > 3 bonds path
605000000	eccentricConnectivityIndex
606000000	FMFDescriptor
607000000	FractionalPSA
608000000	FragmentComplexity
609000000	count of H-acceptor sites (Zefirov PC)
610000000	count of H-donors sites (Zefirov PC)
611000000	HybridationRatio
612000000	[LiD1]-*

613000000	[BeD2](-*)-*
614000000	[BeD4](-*)(-*)(-*)-*
615000000	[BD2H](-*)-*
616000000	[BD3](-*)(-*)-*
617000000	[BD4](-*)(-*)(-*)-*
618000000	[CD1H3]-*
620000000	[CD2H2](-*)-*
621000000	[CD1H]#*
624000000	[CD3H](-*)(-*)-*
626000000	[CD2H0](#*)-*
628000000	[CD4H0](-*)(-*)(-*)-*
629000000	[ND1H3]-*
630000000	[ND1H2]-*
631000000	[ND2H2](-*)-*
633000000	[ND2H](-*)-*
634000000	[N,nD2H](:*):*
635000000	[ND1H0]#*
636000000	[ND3H](-*)(-*)-*
638000000	[N,nD2H0](:*):*
639000000	[ND3H0](-*)(-*)-*
640000000	[ND3H0](~[OD1H0])(~[OD1H0])-,:*
641000000	[N,nD3H0](:*)(:*)-,:*
642000000	[ND4H0](-*)(-*)(-*)-*
643000000	[OD1H]-*
645000000	[OD2H0](-*)-*
646000000	[O,oD2H0](:*):*
647000000	[FD1]-*
648000000	[SiD1H3]-*
649000000	[SiD2H2](-*)-*

650000000	[SiD3H1](-*)(-*)-*
651000000	[SiD4H0](-*)(-*)(-*)-*
652000000	[PD1H2]-*
653000000	[PD2H1](-*)-*
654000000	[PD3H0](-*)(-*)-*
656000000	[PD5H0](-*)(-*)(-*)(-*)-*
657000000	[SD1H1]-*
659000000	[SD2H0](-*)-*
660000000	[S,sD2H0](:*):*
662000000	[SD4H0](~[OD1H0])(~[OD1H0])(-*)-*
663000000	[ClD1]-*
664000000	[GeD1H3](-*)
665000000	[GeD2H2](-*)-*
666000000	[GeD3H1](-*)(-*)-*
667000000	[GeD4H0](-*)(-*)(-*)-*
668000000	[AsD1H2]-*
669000000	[AsD2H1](-*)-*
670000000	[AsD3H0](-*)(-*)-*
672000000	[AsD5H0](-*)(-*)(-*)(-*)-*
673000000	[SeD1H1]-*
675000000	[SeD2H0](-*)-*
676000000	[SeD2H0](:*):*
679000000	[BrD1]-*
680000000	[SnD1H3]-*
681000000	[SnD2H2](-*)-*
682000000	[SnD3H1](-*)(-*)-*
683000000	[SnD4H0](-*)(-*)(-*)-*
684000000	[ID1]-*
685000000	[PbD1H3]-*

686000000	[PbD2H2](-*)-*
687000000	[PbD3H1](-*)(-*)-*
688000000	[PbD4H0](-*)(-*)(-*)-*
689000000	LargestChain
690000000	LargestPISystem
690000000	LargestPISystem
691000000	maximum L/B ratio
692000000	LOBMIN
693000000	longestAliphaticChain
694000000	mannholdLogP
695000000	molecular distance edge between all primary carbons
696000000	molecular distance edge between all primary and secondary carbons
697000000	molecular distance edge between all primary and tertiary carbons
698000000	molecular distance edge between all primary and quaternary carbons
699000000	molecular distance edge between all secondary carbons
700000000	molecular distance edge between all secondary and tertiary carbons
701000000	molecular distance edge between all secondary and quaternary carbons
702000000	molecular distance edge between all tertiary carbons
703000000	molecular distance edge between all tertiary and quaternary carbons
704000000	molecular distance edge between all quaternary carbons
705000000	molecular distance edge between all primary oxygens
706000000	molecular distance edge between all primary and secondary oxygens
707000000	molecular distance edge between all secondary oxygens
708000000	molecular distance edge between all primary nitrogens
709000000	molecular distance edge between all primary and secondary nitrogens
710000000	molecular distance edge between all primary and tertiary niroqens
711000000	molecular distance edge between all secondary nitroqens
712000000	molecular distance edge between all secondary and tertiary nitrogens
713000000	molecular distance edge between all tertiary nitrogens

714000000	PetitJeanNumber
715000000	PetitJeanShapeTopological
716000000	PetitJeanShape D2H](:*):*
717000000	RotatableBondCount-withTerminalwithoutCN
718000000	RotatableBondCount-withTerminalwithCN
719000000	RotatableBondCount-withoutTerminalwithoutCN
720000000	RotatableBondCount-withoutTerminalwithCN
721000000	LipinskyRuleOfI D3H0](:*)(:*)-*
722000000	NSmallRings D3H0](:*)(:*):*
723000000	NAromaticRings
724000000	NRingsBlocks
725000000	NARomaticBlocks
726000000	NRingswith3atoms
727000000	NRingswith4atoms
728000000	NRingswith5atoms
729000000	NRingswith6atoms
730000000	NRingswith7atoms
731000000	NRingswith8atoms
732000000	NRingswith9atoms
733000000	Vertex adjacency information
824000000	XLogP
825000000	ZagrebIndex
826000000	Broto-Moreau autocorrelation lag 0 weighted by mass
827000000	Broto-Moreau autocorrelation lag 1 weighted by mass
828000000	Broto-Moreau autocorrelation lag 2 weighted by mass
829000000	Broto-Moreau autocorrelation lag 3 weighted by mass
830000000	Broto-Moreau autocorrelation lag 4 weighted by mass
831000000	Broto-Moreau autocorrelation lag 5 weighted by mass
832000000	Broto-Moreau autocorrelation lag 6 weighted by mass

833000000	Broto-Moreau autocorrelation lag 7 weighted by mass
834000000	Broto-Moreau autocorrelation lag 8 weighted by mass
835000000	Broto-Moreau autocorrelation lag 0 weighted by van der Waals volumes
836000000	Broto-Moreau autocorrelation lag 1 weighted by van der Waals volumes
837000000	Broto-Moreau autocorrelation lag 2 weighted by van der Waals volumes
838000000	Broto-Moreau autocorrelation lag 3 weighted by van der Waals volumes
839000000	Broto-Moreau autocorrelation lag 4 weighted by van der Waals volumes
840000000	Broto-Moreau autocorrelation lag 5 weighted by van der Waals volumes
841000000	Broto-Moreau autocorrelation lag 6 weighted by van der Waals volumes
842000000	Broto-Moreau autocorrelation lag 7 weighted by van der Waals volumes
843000000	Broto-Moreau autocorrelation lag 8 weighted by van der Waals volumes
844000000	Broto-Moreau autocorrelation lag 0 weighted by Sanderson electronegativities
845000000	Broto-Moreau autocorrelation lag 1 weighted by Sanderson electronegativities
846000000	Broto-Moreau autocorrelation lag 2 weighted by Sanderson electronegativities
847000000	Broto-Moreau autocorrelation lag 3 weighted by Sanderson electronegativities
848000000	Broto-Moreau autocorrelation lag 4 weighted by Sanderson electronegativities
849000000	Broto-Moreau autocorrelation lag 5 weighted by Sanderson electronegativities
850000000	Broto-Moreau autocorrelation lag 6 weighted by Sanderson electronegativities
851000000	Broto-Moreau autocorrelation lag 7 weighted by Sanderson electronegativities
852000000	Broto-Moreau autocorrelation lag 8 weighted by Sanderson electronegativities
853000000	Broto-Moreau autocorrelation lag 0 weighted by polarizabilities
854000000	Broto-Moreau autocorrelation lag 1 weighted by polarizabilities
855000000	Broto-Moreau autocorrelation lag 2 weighted by polarizabilities
856000000	Broto-Moreau autocorrelation lag 3 weighted by polarizabilities
857000000	Broto-Moreau autocorrelation lag 4 weighted by polarizabilities
858000000	Broto-Moreau autocorrelation lag 5 weighted by polarizabilities
859000000	Broto-Moreau autocorrelation lag 6 weighted by polarizabilities
860000000	Broto-Moreau autocorrelation lag 7 weighted by polarizabilities
861000000	Broto-Moreau autocorrelation lag 8 weighted by polarizabilities

862000000	Broto-Moreau autocorrelation lag 0 weighted by first ionization potential
863000000	Broto-Moreau autocorrelation lag 1 weighted by first ionization potential
864000000	Broto-Moreau autocorrelation lag 2 weighted by first ionization potential
865000000	Broto-Moreau autocorrelation lag 3 weighted by first ionization potential
866000000	Broto-Moreau autocorrelation lag 4 weighted by first ionization potential
867000000	Broto-Moreau autocorrelation lag 5 weighted by first ionization potential
868000000	Broto-Moreau autocorrelation lag 6 weighted by first ionization potential
869000000	Broto-Moreau autocorrelation lag 7 weighted by first ionization potential
870000000	Broto-Moreau autocorrelation lag 8 weighted by first ionization potential
871000000	Broto-Moreau autocorrelation lag 0 weighted by I-state
872000000	Broto-Moreau autocorrelation lag 1 weighted by I-state
873000000	Broto-Moreau autocorrelation lag 2 weighted by I-state
874000000	Broto-Moreau autocorrelation lag 3 weighted by I-state
875000000	Broto-Moreau autocorrelation lag 4 weighted by I-state
876000000	Broto-Moreau autocorrelation lag 5 weighted by I-state
877000000	Broto-Moreau autocorrelation lag 6 weighted by I-state
878000000	Broto-Moreau autocorrelation lag 7 weighted by I-state
879000000	Broto-Moreau autocorrelation lag 8 weighted by I-state
880000000	Average Broto-Moreau autocorrelation lag 0 weighted by mass
881000000	Average Broto-Moreau autocorrelation lag 1 weighted by mass
882000000	Average Broto-Moreau autocorrelation lag 2 weighted by mass
883000000	Average Broto-Moreau autocorrelation lag 3 weighted by mass
884000000	Average Broto-Moreau autocorrelation lag 4 weighted by mass
885000000	Average Broto-Moreau autocorrelation lag 5 weighted by mass
886000000	Average Broto-Moreau autocorrelation lag 6 weighted by mass
887000000	Average Broto-Moreau autocorrelation lag 7 weighted by mass
888000000	Average Broto-Moreau autocorrelation lag 8 weighted by mass
889000000	Average Broto-Moreau autocorrelation lag 0 weighted by van der Waals volumes
890000000	Average Broto-Moreau autocorrelation lag 1 weighted by van der Waals volumes

891000000	Average Broto-Moreau autocorrelation lag 2 weighted by van der Waals volumes
892000000	Average Broto-Moreau autocorrelation lag 3 weighted by van der Waals volumes
893000000	Average Broto-Moreau autocorrelation lag 4 weighted by van der Waals volumes
894000000	Average Broto-Moreau autocorrelation lag 5 weighted by van der Waals volumes
895000000	Average Broto-Moreau autocorrelation lag 6 weighted by van der Waals volumes
896000000	Average Broto-Moreau autocorrelation lag 7 weighted by van der Waals volumes
897000000	Average Broto-Moreau autocorrelation lag 8 weighted by van der Waals volumes
898000000	Average Broto-Moreau autocorrelation lag 0 weighted by Sanderson electronegativities
899000000	Average Broto-Moreau autocorrelation lag 1 weighted by Sanderson electronegativities
900000000	Average Broto-Moreau autocorrelation lag 2 weighted by Sanderson electronegativities
901000000	Average Broto-Moreau autocorrelation lag 3 weighted by Sanderson electronegativities
902000000	Average Broto-Moreau autocorrelation lag 4 weighted by Sanderson electronegativities
903000000	Average Broto-Moreau autocorrelation lag 5 weighted by Sanderson electronegativities
904000000	Average Broto-Moreau autocorrelation lag 6 weighted by Sanderson electronegativities
905000000	Average Broto-Moreau autocorrelation lag 7 weighted by Sanderson electronegativities
906000000	Average Broto-Moreau autocorrelation lag 8 weighted by Sanderson electronegativities
907000000	Average Broto-Moreau autocorrelation lag 0 weighted by polarizabilities
908000000	Average Broto-Moreau autocorrelation lag 1 weighted by polarizabilities
909000000	Average Broto-Moreau autocorrelation lag 2 weighted by polarizabilities
910000000	Average Broto-Moreau autocorrelation lag 3 weighted by polarizabilities
911000000	Average Broto-Moreau autocorrelation lag 4 weighted by polarizabilities
912000000	Average Broto-Moreau autocorrelation lag 5 weighted by polarizabilities
913000000	Average Broto-Moreau autocorrelation lag 6 weighted by polarizabilities
914000000	Average Broto-Moreau autocorrelation lag 7 weighted by polarizabilities
915000000	Average Broto-Moreau autocorrelation lag 8 weighted by polarizabilities
916000000	Average Broto-Moreau autocorrelation lag 0 weighted by first ionization potential
917000000	Average Broto-Moreau autocorrelation lag 1 weighted by first ionization potential
918000000	Average Broto-Moreau autocorrelation lag 2 weighted by first ionization potential
919000000	Average Broto-Moreau autocorrelation lag 3 weighted by first ionization potential

920000000	Average Broto-Moreau autocorrelation lag 4 weighted by first ionization potential
921000000	Average Broto-Moreau autocorrelation lag 5 weighted by first ionization potential
922000000	Average Broto-Moreau autocorrelation lag 6 weighted by first ionization potential
923000000	Average Broto-Moreau autocorrelation lag 7 weighted by first ionization potential
924000000	Average Broto-Moreau autocorrelation lag 8 weighted by first ionization potential
925000000	Average Broto-Moreau autocorrelation lag 0 weighted by I-state
926000000	Average Broto-Moreau autocorrelation lag 1 weighted by I-state
927000000	Average Broto-Moreau autocorrelation lag 2 weighted by I-state
928000000	Average Broto-Moreau autocorrelation lag 3 weighted by I-state
929000000	Average Broto-Moreau autocorrelation lag 4 weighted by I-state
930000000	Average Broto-Moreau autocorrelation lag 5 weighted by I-state
931000000	Average Broto-Moreau autocorrelation lag 6 weighted by I-state
932000000	Average Broto-Moreau autocorrelation lag 7 weighted by I-state
933000000	Average Broto-Moreau autocorrelation lag 8 weighted by I-state
934000000	Centered Broto-Moreau autocorrelation lag 0 weighted by charges
935000000	Centered Broto-Moreau autocorrelation lag 1 weighted by charges
936000000	Centered Broto-Moreau autocorrelation lag 2 weighted by charges
937000000	Centered Broto-Moreau autocorrelation lag 3 weighted by charges
938000000	Centered Broto-Moreau autocorrelation lag 4 weighted by charges
939000000	Centered Broto-Moreau autocorrelation lag 5 weighted by charges
940000000	Centered Broto-Moreau autocorrelation lag 6 weighted by charges
941000000	Centered Broto-Moreau autocorrelation lag 7 weighted by charges
942000000	Centered Broto-Moreau autocorrelation lag 8 weighted by charges
943000000	Centered Broto-Moreau autocorrelation lag 0 weighted by mass
944000000	Centered Broto-Moreau autocorrelation lag 1 weighted by mass
945000000	Centered Broto-Moreau autocorrelation lag 2 weighted by mass
946000000	Centered Broto-Moreau autocorrelation lag 3 weighted by mass
947000000	Centered Broto-Moreau autocorrelation lag 4 weighted by mass
948000000	Centered Broto-Moreau autocorrelation lag 5 weighted by mass

949000000	Centered Broto-Moreau autocorrelation lag 6 weighted by mass
950000000	Centered Broto-Moreau autocorrelation lag 7 weighted by mass
951000000	Centered Broto-Moreau autocorrelation lag 8 weighted by mass
952000000	Centered Broto-Moreau autocorrelation lag 0 weighted by van der Waals volumes
953000000	Centered Broto-Moreau autocorrelation lag 1 weighted by van der Waals volumes
954000000	Centered Broto-Moreau autocorrelation lag 2 weighted by van der Waals volumes
955000000	Centered Broto-Moreau autocorrelation lag 3 weighted by van der Waals volumes
956000000	Centered Broto-Moreau autocorrelation lag 4 weighted by van der Waals volumes
957000000	Centered Broto-Moreau autocorrelation lag 5 weighted by van der Waals volumes
958000000	Centered Broto-Moreau autocorrelation lag 6 weighted by van der Waals volumes
959000000	Centered Broto-Moreau autocorrelation lag 7 weighted by van der Waals volumes
960000000	Centered Broto-Moreau autocorrelation lag 8 weighted by van der Waals volumes
961000000	Centered Broto-Moreau autocorrelation lag 0 weighted by Sanderson electronegativities
962000000	Centered Broto-Moreau autocorrelation lag 1 weighted by Sanderson electronegativities
963000000	Centered Broto-Moreau autocorrelation lag 2 weighted by Sanderson electronegativities
964000000	Centered Broto-Moreau autocorrelation lag 3 weighted by Sanderson electronegativities
965000000	Centered Broto-Moreau autocorrelation lag 4 weighted by Sanderson electronegativities
966000000	Centered Broto-Moreau autocorrelation lag 5 weighted by Sanderson electronegativities
967000000	Centered Broto-Moreau autocorrelation lag 6 weighted by Sanderson electronegativities
968000000	Centered Broto-Moreau autocorrelation lag 7 weighted by Sanderson electronegativities
969000000	Centered Broto-Moreau autocorrelation lag 8 weighted by Sanderson electronegativities
970000000	Centered Broto-Moreau autocorrelation lag 0 weighted by polarizabilities
971000000	Centered Broto-Moreau autocorrelation lag 1 weighted by polarizabilities
972000000	Centered Broto-Moreau autocorrelation lag 2 weighted by polarizabilities
973000000	Centered Broto-Moreau autocorrelation lag 3 weighted by polarizabilities
974000000	Centered Broto-Moreau autocorrelation lag 4 weighted by polarizabilities
975000000	Centered Broto-Moreau autocorrelation lag 5 weighted by polarizabilities
976000000	Centered Broto-Moreau autocorrelation lag 6 weighted by polarizabilities
977000000	Centered Broto-Moreau autocorrelation lag 7 weighted by polarizabilities

978000000	Centered Broto-Moreau autocorrelation lag 8 weighted by polarizabilities
979000000	Centered Broto-Moreau autocorrelation lag 0 weighted by first ionization potential
980000000	Centered Broto-Moreau autocorrelation lag 1 weighted by first ionization potential
981000000	Centered Broto-Moreau autocorrelation lag 2 weighted by first ionization potential
982000000	Centered Broto-Moreau autocorrelation lag 3 weighted by first ionization potential
983000000	Centered Broto-Moreau autocorrelation lag 4 weighted by first ionization potential
984000000	Centered Broto-Moreau autocorrelation lag 5 weighted by first ionization potential
985000000	Centered Broto-Moreau autocorrelation lag 6 weighted by first ionization potential
986000000	Centered Broto-Moreau autocorrelation lag 7 weighted by first ionization potential
987000000	Centered Broto-Moreau autocorrelation lag 8 weighted by first ionization potential
988000000	Centered Broto-Moreau autocorrelation lag 0 weighted by I-state
989000000	Centered Broto-Moreau autocorrelation lag 1 weighted by I-state
990000000	Centered Broto-Moreau autocorrelation lag 2 weighted by I-state
991000000	Centered Broto-Moreau autocorrelation lag 3 weighted by I-state
992000000	Centered Broto-Moreau autocorrelation lag 4 weighted by I-state
993000000	Centered Broto-Moreau autocorrelation lag 5 weighted by I-state
994000000	Centered Broto-Moreau autocorrelation lag 6 weighted by I-state
995000000	Centered Broto-Moreau autocorrelation lag 7 weighted by I-state
996000000	Centered Broto-Moreau autocorrelation lag 8 weighted by I-state
997000000	Average centered Broto-Moreau autocorrelation lag 0 weighted by charges
998000000	Average centered Broto-Moreau autocorrelation lag 1 weighted by charges
999000000	Average centered Broto-Moreau autocorrelation lag 2 weighted by charges
1000000000	Average centered Broto-Moreau autocorrelation lag 3 weighted by charges
1001000000	Average centered Broto-Moreau autocorrelation lag 4 weighted by charges
1002000000	Average centered Broto-Moreau autocorrelation lag 5 weighted by charges
1003000000	Average centered Broto-Moreau autocorrelation lag 6 weighted by charges
1004000000	Average centered Broto-Moreau autocorrelation lag 7 weighted by charges
1005000000	Average centered Broto-Moreau autocorrelation lag 8 weighted by charges
1006000000	Average centered Broto-Moreau autocorrelation lag 0 weighted by mass

1007000000	Average centered Broto-Moreau autocorrelation lag 1 weighted by mass
1008000000	Average centered Broto-Moreau autocorrelation lag 2 weighted by mass
1009000000	Average centered Broto-Moreau autocorrelation lag 3 weighted by mass
1010000000	Average centered Broto-Moreau autocorrelation lag 4 weighted by mass
1011000000	Average centered Broto-Moreau autocorrelation lag 5 weighted by mass
1012000000	Average centered Broto-Moreau autocorrelation lag 6 weighted by mass
1013000000	Average centered Broto-Moreau autocorrelation lag 7 weighted by mass
1014000000	Average centered Broto-Moreau autocorrelation lag 8 weighted by mass
1015000000	Average centered Broto-Moreau autocorrelation lag 0 weighted by van der Waals volumes
1016000000	Average centered Broto-Moreau autocorrelation lag 1 weighted by van der Waals volumes
1017000000	Average centered Broto-Moreau autocorrelation lag 2 weighted by van der Waals volumes
1018000000	Average centered Broto-Moreau autocorrelation lag 3 weighted by van der Waals volumes
1019000000	Average centered Broto-Moreau autocorrelation lag 4 weighted by van der Waals volumes
1020000000	Average centered Broto-Moreau autocorrelation lag 5 weighted by van der Waals volumes
1021000000	Average centered Broto-Moreau autocorrelation lag 6 weighted by van der Waals volumes
1022000000	Average centered Broto-Moreau autocorrelation lag 7 weighted by van der Waals volumes
1023000000	Average centered Broto-Moreau autocorrelation lag 8 weighted by van der Waals volumes
1024000000	Average centered Broto-Moreau autocorrelation lag 0 weighted by Sanderson electronegativities
1025000000	Average centered Broto-Moreau autocorrelation lag 1 weighted by Sanderson electronegativities
1026000000	Average centered Broto-Moreau autocorrelation lag 2 weighted by Sanderson electronegativities
1027000000	Average centered Broto-Moreau autocorrelation lag 3 weighted by Sanderson electronegativities
1028000000	Average centered Broto-Moreau autocorrelation lag 4 weighted by Sanderson electronegativities
1029000000	Average centered Broto-Moreau autocorrelation lag 5 weighted by Sanderson electronegativities
1030000000	Average centered Broto-Moreau autocorrelation lag 6 weighted by Sanderson electronegativities
1031000000	Average centered Broto-Moreau autocorrelation lag 7 weighted by Sanderson electronegativities
1032000000	Average centered Broto-Moreau autocorrelation lag 8 weighted by Sanderson electronegativities
1033000000	Average centered Broto-Moreau autocorrelation lag 0 weighted by polarizabilities
1034000000	Average centered Broto-Moreau autocorrelation lag 1 weighted by polarizabilities
1035000000	Average centered Broto-Moreau autocorrelation lag 2 weighted by polarizabilities

1036000000 Average centered Broto-Moreau autocorrelation lag 3 weighted by polarizabilities
1037000000 Average centered Broto-Moreau autocorrelation lag 4 weighted by polarizabilities
1038000000 Average centered Broto-Moreau autocorrelation lag 5 weighted by polarizabilities
1039000000 Average centered Broto-Moreau autocorrelation lag 6 weighted by polarizabilities
1040000000 Average centered Broto-Moreau autocorrelation lag 7 weighted by polarizabilities
1041000000 Average centered Broto-Moreau autocorrelation lag 8 weighted by polarizabilities
1042000000 Average centered Broto-Moreau autocorrelation lag 0 weighted by first ionization potential
1043000000 Average centered Broto-Moreau autocorrelation lag 1 weighted by first ionization potential
1044000000 Average centered Broto-Moreau autocorrelation lag 2 weighted by first ionization potential
1045000000 Average centered Broto-Moreau autocorrelation lag 3 weighted by first ionization potential
1046000000 Average centered Broto-Moreau autocorrelation lag 4 weighted by first ionization potential
1047000000 Average centered Broto-Moreau autocorrelation lag 5 weighted by first ionization potential
1048000000 Average centered Broto-Moreau autocorrelation lag 6 weighted by first ionization potential
1049000000 Average centered Broto-Moreau autocorrelation lag 7 weighted by first ionization potential
1050000000 Average centered Broto-Moreau autocorrelation lag 8 weighted by first ionization potential
1051000000 Average centered Broto-Moreau autocorrelation lag 0 weighted by I-state
1052000000 Average centered Broto-Moreau autocorrelation lag 1 weighted by I-state
1053000000 Average centered Broto-Moreau autocorrelation lag 2 weighted by I-state
1054000000 Average centered Broto-Moreau autocorrelation lag 3 weighted by I-state
1055000000 Average centered Broto-Moreau autocorrelation lag 4 weighted by I-state
1056000000 Average centered Broto-Moreau autocorrelation lag 5 weighted by I-state
1057000000 Average centered Broto-Moreau autocorrelation lag 6 weighted by I-state
1058000000 Average centered Broto-Moreau autocorrelation lag 7 weighted by I-state
1059000000 Average centered Broto-Moreau autocorrelation lag 8 weighted by I-state
1060000000 Moran autocorrelation lag 1 weighted by charges
1061000000 Moran autocorrelation lag 2 weighted by charges
1062000000 Moran autocorrelation lag 3 weighted by charges
1063000000 Moran autocorrelation lag 4 weighted by charges
1064000000 Moran autocorrelation lag 5 weighted by charges

1065000000	Moran autocorrelation lag 6 weighted by charges
1066000000	Moran autocorrelation lag 7 weighted by charges
1067000000	Moran autocorrelation lag 8 weighted by charges
1068000000	Moran autocorrelation lag 1 weighted by mass
1069000000	Moran autocorrelation lag 2 weighted by mass
1070000000	Moran autocorrelation lag 3 weighted by mass
1071000000	Moran autocorrelation lag 4 weighted by mass
1072000000	Moran autocorrelation lag 5 weighted by mass
1073000000	Moran autocorrelation lag 6 weighted by mass
1074000000	Moran autocorrelation lag 7 weighted by mass
1075000000	Moran autocorrelation lag 8 weighted by mass
1076000000	Moran autocorrelation lag 1 weighted by van der Waals volumes
1077000000	Moran autocorrelation lag 2 weighted by van der Waals volumes
1078000000	Moran autocorrelation lag 3 weighted by van der Waals volumes
1079000000	Moran autocorrelation lag 4 weighted by van der Waals volumes
1080000000	Moran autocorrelation lag 5 weighted by van der Waals volumes
1081000000	Moran autocorrelation lag 6 weighted by van der Waals volumes
1082000000	Moran autocorrelation lag 7 weighted by van der Waals volumes
1083000000	Moran autocorrelation lag 8 weighted by van der Waals volumes
1084000000	Moran autocorrelation lag 1 weighted by Sanderson electronegativities
1085000000	Moran autocorrelation lag 2 weighted by Sanderson electronegativities
1086000000	Moran autocorrelation lag 3 weighted by Sanderson electronegativities
1087000000	Moran autocorrelation lag 4 weighted by Sanderson electronegativities
1088000000	Moran autocorrelation lag 5 weighted by Sanderson electronegativities
1089000000	Moran autocorrelation lag 6 weighted by Sanderson electronegativities
1090000000	Moran autocorrelation lag 7 weighted by Sanderson electronegativities
1091000000	Moran autocorrelation lag 8 weighted by Sanderson electronegativities
1092000000	Moran autocorrelation lag 1 weighted by polarizabilities
1093000000	Moran autocorrelation lag 2 weighted by polarizabilities

1094000000	Moran autocorrelation lag 3 weighted by polarizabilities
1095000000	Moran autocorrelation lag 4 weighted by polarizabilities
1096000000	Moran autocorrelation lag 5 weighted by polarizabilities
1097000000	Moran autocorrelation lag 6 weighted by polarizabilities
1098000000	Moran autocorrelation lag 7 weighted by polarizabilities
1099000000	Moran autocorrelation lag 8 weighted by polarizabilities
1100000000	Moran autocorrelation lag 1 weighted by first ionization potential
1101000000	Moran autocorrelation lag 2 weighted by first ionization potential
1102000000	Moran autocorrelation lag 3 weighted by first ionization potential
1103000000	Moran autocorrelation lag 4 weighted by first ionization potential
1104000000	Moran autocorrelation lag 5 weighted by first ionization potential
1105000000	Moran autocorrelation lag 6 weighted by first ionization potential
1106000000	Moran autocorrelation lag 7 weighted by first ionization potential
1107000000	Moran autocorrelation lag 8 weighted by first ionization potential
1108000000	Moran autocorrelation lag 1 weighted by I-state
1109000000	Moran autocorrelation lag 2 weighted by I-state
1110000000	Moran autocorrelation lag 3 weighted by I-state
1111000000	Moran autocorrelation lag 4 weighted by I-state
1112000000	Moran autocorrelation lag 5 weighted by I-state
1113000000	Moran autocorrelation lag 6 weighted by I-state
1114000000	Moran autocorrelation lag 7 weighted by I-state
1115000000	Moran autocorrelation lag 8 weighted by I-state
1116000000	Geary autocorrelation lag 1 weighted by charges
1117000000	Geary autocorrelation lag 2 weighted by charges
1118000000	Geary autocorrelation lag 3 weighted by charges
1119000000	Geary autocorrelation lag 4 weighted by charges
1120000000	Geary autocorrelation lag 5 weighted by charges
1121000000	Geary autocorrelation lag 6 weighted by charges
1122000000	Geary autocorrelation lag 7 weighted by charges

1123000000	Geary autocorrelation lag 8 weighted by charges
1124000000	Geary autocorrelation lag 1 weighted by mass
1125000000	Geary autocorrelation lag 2 weighted by mass
1126000000	Geary autocorrelation lag 3 weighted by mass
1127000000	Geary autocorrelation lag 4 weighted by mass
1128000000	Geary autocorrelation lag 5 weighted by mass
1129000000	Geary autocorrelation lag 6 weighted by mass
1130000000	Geary autocorrelation lag 7 weighted by mass
1131000000	Geary autocorrelation lag 8 weighted by mass
1132000000	Geary autocorrelation lag 1 weighted by van der Waals volumes
1133000000	Geary autocorrelation lag 2 weighted by van der Waals volumes
1134000000	Geary autocorrelation lag 3 weighted by van der Waals volumes
1135000000	Geary autocorrelation lag 4 weighted by van der Waals volumes
1136000000	Geary autocorrelation lag 5 weighted by van der Waals volumes
1137000000	Geary autocorrelation lag 6 weighted by van der Waals volumes
1138000000	Geary autocorrelation lag 7 weighted by van der Waals volumes
1139000000	Geary autocorrelation lag 8 weighted by van der Waals volumes
1140000000	Geary autocorrelation lag 1 weighted by Sanderson electronegativities
1141000000	Geary autocorrelation lag 2 weighted by Sanderson electronegativities
1142000000	Geary autocorrelation lag 3 weighted by Sanderson electronegativities
1143000000	Geary autocorrelation lag 4 weighted by Sanderson electronegativities
1144000000	Geary autocorrelation lag 5 weighted by Sanderson electronegativities
1145000000	Geary autocorrelation lag 6 weighted by Sanderson electronegativities
1146000000	Geary autocorrelation lag 7 weighted by Sanderson electronegativities
1147000000	Geary autocorrelation lag 8 weighted by Sanderson electronegativities
1148000000	Geary autocorrelation lag 1 weighted by polarizabilities
1149000000	Geary autocorrelation lag 2 weighted by polarizabilities
1150000000	Geary autocorrelation lag 3 weighted by polarizabilities
1151000000	Geary autocorrelation lag 4 weighted by polarizabilities

1152000000	Geary autocorrelation lag 5 weighted by polarizabilities
1153000000	Geary autocorrelation lag 6 weighted by polarizabilities
1154000000	Geary autocorrelation lag 7 weighted by polarizabilities
1155000000	Geary autocorrelation lag 8 weighted by polarizabilities
1156000000	Geary autocorrelation lag 1 weighted by first ionization potential
1157000000	Geary autocorrelation lag 2 weighted by first ionization potential
1158000000	Geary autocorrelation lag 3 weighted by first ionization potential
1159000000	Geary autocorrelation lag 4 weighted by first ionization potential
1160000000	Geary autocorrelation lag 5 weighted by first ionization potential
1161000000	Geary autocorrelation lag 6 weighted by first ionization potential
1162000000	Geary autocorrelation lag 7 weighted by first ionization potential
1163000000	Geary autocorrelation lag 8 weighted by first ionization potential
1164000000	Geary autocorrelation lag 1 weighted by I-state
1165000000	Geary autocorrelation lag 2 weighted by I-state
1166000000	Geary autocorrelation lag 3 weighted by I-state
1167000000	Geary autocorrelation lag 4 weighted by I-state
1168000000	Geary autocorrelation lag 5 weighted by I-state
1169000000	Geary autocorrelation lag 6 weighted by I-state
1170000000	Geary autocorrelation lag 7 weighted by I-state
1171000000	Geary autocorrelation lag 8 weighted by I-state
1172000000	Largest absolute eigenvalue of Burden modified matrix - n 1 weighted by relative mass
1173000000	Largest absolute eigenvalue of Burden modified matrix - n 2 weighted by relative mass
1174000000	Largest absolute eigenvalue of Burden modified matrix - n 3 weighted by relative mass
1175000000	Largest absolute eigenvalue of Burden modified matrix - n 4 weighted by relative mass
1176000000	Largest absolute eigenvalue of Burden modified matrix - n 5 weighted by relative mass
1177000000	Largest absolute eigenvalue of Burden modified matrix - n 6 weighted by relative mass
1178000000	Largest absolute eigenvalue of Burden modified matrix - n 7 weighted by relative mass
1179000000	Largest absolute eigenvalue of Burden modified matrix - n 8 weighted by relative mass
1180000000	Smallest absolute eigenvalue of Burden modified matrix - n 1 weighted by relative mass

1181000000	Smallest absolute eigenvalue of Burden modified matrix - n 2 weighted by relative mass
1182000000	Smallest absolute eigenvalue of Burden modified matrix - n 3 weighted by relative mass
1183000000	Smallest absolute eigenvalue of Burden modified matrix - n 4 weighted by relative mass
1184000000	Smallest absolute eigenvalue of Burden modified matrix - n 5 weighted by relative mass
1185000000	Smallest absolute eigenvalue of Burden modified matrix - n 6 weighted by relative mass
1186000000	Smallest absolute eigenvalue of Burden modified matrix - n 7 weighted by relative mass
1187000000	Smallest absolute eigenvalue of Burden modified matrix - n 8 weighted by relative mass
1188000000	Largest absolute eigenvalue of Burden modified matrix - n 1 weighted by relative van der Waals volumes
1189000000	Largest absolute eigenvalue of Burden modified matrix - n 2 weighted by relative van der Waals volumes
1190000000	Largest absolute eigenvalue of Burden modified matrix - n 3 weighted by relative van der Waals volumes
1191000000	Largest absolute eigenvalue of Burden modified matrix - n 4 weighted by relative van der Waals volumes
1192000000	Largest absolute eigenvalue of Burden modified matrix - n 5 weighted by relative van der Waals volumes
1193000000	Largest absolute eigenvalue of Burden modified matrix - n 6 weighted by relative van der Waals volumes
1194000000	Largest absolute eigenvalue of Burden modified matrix - n 7 weighted by relative van der Waals volumes
1195000000	Largest absolute eigenvalue of Burden modified matrix - n 8 weighted by relative van der Waals volumes
1196000000	Smallest absolute eigenvalue of Burden modified matrix - n 1 weighted by relative van der Waals volumes
1197000000	Smallest absolute eigenvalue of Burden modified matrix - n 2 weighted by relative van der Waals volumes
1198000000	Smallest absolute eigenvalue of Burden modified matrix - n 3 weighted by relative van der Waals volumes
1199000000	Smallest absolute eigenvalue of Burden modified matrix - n 4 weighted by relative van der Waals volumes
1200000000	Smallest absolute eigenvalue of Burden modified matrix - n 5 weighted by relative van der Waals volumes
1201000000	Smallest absolute eigenvalue of Burden modified matrix - n 6 weighted by relative van der Waals volumes
1202000000	Smallest absolute eigenvalue of Burden modified matrix - n 7 weighted by relative van der Waals volumes
1203000000	Smallest absolute eigenvalue of Burden modified matrix - n 8 weighted by relative van der Waals volumes
1204000000	Largest absolute eigenvalue of Burden modified matrix - n 1 weighted by relative Sanderson electronegativities
1205000000	Largest absolute eigenvalue of Burden modified matrix - n 2 weighted by relative Sanderson electronegativities
1206000000	Largest absolute eigenvalue of Burden modified matrix - n 3 weighted by relative Sanderson electronegativities
1207000000	Largest absolute eigenvalue of Burden modified matrix - n 4 weighted by relative Sanderson electronegativities
1208000000	Largest absolute eigenvalue of Burden modified matrix - n 5 weighted by relative Sanderson electronegativities
1209000000	Largest absolute eigenvalue of Burden modified matrix - n 6 weighted by relative Sanderson electronegativities

1210000000 Largest absolute eigenvalue of Burden modified matrix - n 7 weighted by relative Sanderson electronegativities
1211000000 Largest absolute eigenvalue of Burden modified matrix - n 8 weighted by relative Sanderson electronegativities
1212000000 Smallest absolute eigenvalue of Burden modified matrix - n 1 weighted by relative Sanderson electronegativities
1213000000 Smallest absolute eigenvalue of Burden modified matrix - n 2 weighted by relative Sanderson electronegativities
1214000000 Smallest absolute eigenvalue of Burden modified matrix - n 3 weighted by relative Sanderson electronegativities
1215000000 Smallest absolute eigenvalue of Burden modified matrix - n 4 weighted by relative Sanderson electronegativities
1216000000 Smallest absolute eigenvalue of Burden modified matrix - n 5 weighted by relative Sanderson electronegativities
1217000000 Smallest absolute eigenvalue of Burden modified matrix - n 6 weighted by relative Sanderson electronegativities
1218000000 Smallest absolute eigenvalue of Burden modified matrix - n 7 weighted by relative Sanderson electronegativities
1219000000 Smallest absolute eigenvalue of Burden modified matrix - n 8 weighted by relative Sanderson electronegativities
1220000000 Largest absolute eigenvalue of Burden modified matrix - n 1 weighted by relative polarizabilities
1221000000 Largest absolute eigenvalue of Burden modified matrix - n 2 weighted by relative polarizabilities
1222000000 Largest absolute eigenvalue of Burden modified matrix - n 3 weighted by relative polarizabilities
1223000000 Largest absolute eigenvalue of Burden modified matrix - n 4 weighted by relative polarizabilities
1224000000 Largest absolute eigenvalue of Burden modified matrix - n 5 weighted by relative polarizabilities
1225000000 Largest absolute eigenvalue of Burden modified matrix - n 6 weighted by relative polarizabilities
1226000000 Largest absolute eigenvalue of Burden modified matrix - n 7 weighted by relative polarizabilities
1227000000 Largest absolute eigenvalue of Burden modified matrix - n 8 weighted by relative polarizabilities
1228000000 Smallest absolute eigenvalue of Burden modified matrix - n 1 weighted by relative polarizabilities
1229000000 Smallest absolute eigenvalue of Burden modified matrix - n 2 weighted by relative polarizabilities
1230000000 Smallest absolute eigenvalue of Burden modified matrix - n 3 weighted by relative polarizabilities
1231000000 Smallest absolute eigenvalue of Burden modified matrix - n 4 weighted by relative polarizabilities
1232000000 Smallest absolute eigenvalue of Burden modified matrix - n 5 weighted by relative polarizabilities
1233000000 Smallest absolute eigenvalue of Burden modified matrix - n 6 weighted by relative polarizabilities
1234000000 Smallest absolute eigenvalue of Burden modified matrix - n 7 weighted by relative polarizabilities
1235000000 Smallest absolute eigenvalue of Burden modified matrix - n 8 weighted by relative polarizabilities
1236000000 Largest absolute eigenvalue of Burden modified matrix - n 1 weighted by relative first ionization potential
1237000000 Largest absolute eigenvalue of Burden modified matrix - n 2 weighted by relative first ionization potential
1238000000 Largest absolute eigenvalue of Burden modified matrix - n 3 weighted by relative first ionization potential

1239000000 Largest absolute eigenvalue of Burden modified matrix - n 4 weighted by relative first ionization potential
1240000000 Largest absolute eigenvalue of Burden modified matrix - n 5 weighted by relative first ionization potential
1241000000 Largest absolute eigenvalue of Burden modified matrix - n 6 weighted by relative first ionization potential
1242000000 Largest absolute eigenvalue of Burden modified matrix - n 7 weighted by relative first ionization potential
1243000000 Largest absolute eigenvalue of Burden modified matrix - n 8 weighted by relative first ionization potential
1244000000 Smallest absolute eigenvalue of Burden modified matrix - n 1 weighted by relative first ionization potential
1245000000 Smallest absolute eigenvalue of Burden modified matrix - n 2 weighted by relative first ionization potential
1246000000 Smallest absolute eigenvalue of Burden modified matrix - n 3 weighted by relative first ionization potential
1247000000 Smallest absolute eigenvalue of Burden modified matrix - n 4 weighted by relative first ionization potential
1248000000 Smallest absolute eigenvalue of Burden modified matrix - n 5 weighted by relative first ionization potential
1249000000 Smallest absolute eigenvalue of Burden modified matrix - n 6 weighted by relative first ionization potential
1250000000 Smallest absolute eigenvalue of Burden modified matrix - n 7 weighted by relative first ionization potential
1251000000 Smallest absolute eigenvalue of Burden modified matrix - n 8 weighted by relative first ionization potential
1252000000 Largest absolute eigenvalue of Burden modified matrix - n 1 weighted by relative I-state
1253000000 Largest absolute eigenvalue of Burden modified matrix - n 2 weighted by relative I-state
1254000000 Largest absolute eigenvalue of Burden modified matrix - n 3 weighted by relative I-state
1255000000 Largest absolute eigenvalue of Burden modified matrix - n 4 weighted by relative I-state
1256000000 Largest absolute eigenvalue of Burden modified matrix - n 5 weighted by relative I-state
1257000000 Largest absolute eigenvalue of Burden modified matrix - n 6 weighted by relative I-state
1258000000 Largest absolute eigenvalue of Burden modified matrix - n 7 weighted by relative I-state
1259000000 Largest absolute eigenvalue of Burden modified matrix - n 8 weighted by relative I-state
1260000000 Smallest absolute eigenvalue of Burden modified matrix - n 1 weighted by relative I-state
1261000000 Smallest absolute eigenvalue of Burden modified matrix - n 2 weighted by relative I-state
1262000000 Smallest absolute eigenvalue of Burden modified matrix - n 3 weighted by relative I-state
1263000000 Smallest absolute eigenvalue of Burden modified matrix - n 4 weighted by relative I-state
1264000000 Smallest absolute eigenvalue of Burden modified matrix - n 5 weighted by relative I-state
1265000000 Smallest absolute eigenvalue of Burden modified matrix - n 6 weighted by relative I-state
1266000000 Smallest absolute eigenvalue of Burden modified matrix - n 7 weighted by relative I-state
1267000000 Smallest absolute eigenvalue of Burden modified matrix - n 8 weighted by relative I-state

1268000000	Number of 3-membered rings
1269000000	Number of 4-membered rings
1270000000	Number of 5-membered rings
1271000000	Number of 6-membered rings
1272000000	Number of 7-membered rings
1273000000	Number of 8-membered rings
1274000000	Number of 9-membered rings
1275000000	Number of 10-membered rings
1276000000	Number of 11-membered rings
1277000000	Number of 12-membered rings
1278000000	Number of >12-membered rings
1279000000	Number of fused rings
1280000000	Number of 4-membered fused rings
1281000000	Number of 5-membered fused rings
1282000000	Number of 6-membered fused rings
1283000000	Number of 7-membered fused rings
1284000000	Min resonance energy for bond H - N
1285000000	Max resonance energy for bond H - N
1286000000	Min exchange energy for bond H - N
1287000000	Max exchange energy for bond H - N
1288000000	Min e-e repulsion for bond H - N
1289000000	Max e-e repulsion for bond H - N
1290000000	Min e-n attraction for bond H - N
1291000000	Max e-n attraction for bond H - N
1292000000	Min n-n repulsion for bond H - N
1293000000	Max n-n repulsion for bond H - N
1294000000	Min coulombic interaction for bond H - N
1295000000	Max coulombic interaction for bond H - N
1296000000	Min total interaction for bond H - N

1297000000 Max total interaction for bond H - N
1298000000 Min valency for atom Cl
1299000000 Max valency for atom Cl
1300000000 Min (>0.1) bond order for atom Cl
1301000000 Max bond order for atom Cl
1302000000 Min e-e repulsion for atom Cl
1303000000 Max e-e repulsion for atom Cl
1304000000 Min e-n attraction for atom Cl
1305000000 Max e-n attraction for atom Cl
1306000000 Min atomic state energy for atom Cl
1307000000 Max atomic state energy for atom Cl
1308000000 Min resonance energy for bond C - Cl
1309000000 Max resonance energy for bond C - Cl
1310000000 Min exchange energy for bond C - Cl
1311000000 Max exchange energy for bond C - Cl
1312000000 Min e-e repulsion for bond C - Cl
1313000000 Max e-e repulsion for bond C - Cl
1314000000 Min e-n attraction for bond C - Cl
1315000000 Max e-n attraction for bond C - Cl
1316000000 Min n-n repulsion for bond C - Cl
1317000000 Max n-n repulsion for bond C - Cl
1318000000 Min coulombic interaction for bond C - Cl
1319000000 Max coulombic interaction for bond C - Cl
1320000000 Min total interaction for bond C - Cl
1321000000 Max total interaction for bond C - Cl
1322000000 Min resonance energy for interaction C - Cl in 2 bonds path
1323000000 Max resonance energy for interaction C - Cl in 2 bonds path
1324000000 Min exchange energy for interaction C - Cl in 2 bonds path
1325000000 Max exchange energy for interaction C - Cl in 2 bonds path

1326000000 Min e-e repulsion for interaction C - Cl in 2 bonds path
1327000000 Max e-e repulsion for interaction C - Cl in 2 bonds path
1328000000 Min e-n attraction for interaction C - Cl in 2 bonds path
1329000000 Max e-n attraction for interaction C - Cl in 2 bonds path
1330000000 Min n-n repulsion for interaction C - Cl in 2 bonds path
1331000000 Max n-n repulsion for interaction C - Cl in 2 bonds path
1332000000 Min coulumbic interaction for interaction C - Cl in 2 bonds path
1333000000 Max coulumbic interaction for interaction C - Cl in 2 bonds path
1334000000 Min total interaction for interaction C - Cl in 2 bonds path
1335000000 Max total interaction for interaction C - Cl in 2 bonds path
1336000000 Min resonance energy for interaction C - Cl in 3 bonds path
1337000000 Max resonance energy for interaction C - Cl in 3 bonds path
1338000000 Min exchange energy for interaction C - Cl in 3 bonds path
1339000000 Max exchange energy for interaction C - Cl in 3 bonds path
1340000000 Min e-e repulsion for interaction C - Cl in 3 bonds path
1341000000 Max e-e repulsion for interaction C - Cl in 3 bonds path
1342000000 Min e-n attraction for interaction C - Cl in 3 bonds path
1343000000 Max e-n attraction for interaction C - Cl in 3 bonds path
1344000000 Min n-n repulsion for interaction C - Cl in 3 bonds path
1345000000 Max n-n repulsion for interaction C - Cl in 3 bonds path
1346000000 Min coulumbic interaction for interaction C - Cl in 3 bonds path
1347000000 Max coulumbic interaction for interaction C - Cl in 3 bonds path
1348000000 Min total interaction for interaction C - Cl in 3 bonds path
1349000000 Max total interaction for interaction C - Cl in 3 bonds path
1350000000 Min resonance energy for interaction C - Cl in > 3 bonds path
1351000000 Max resonance energy for interaction C - Cl in > 3 bonds path
1352000000 Min exchange energy for interaction C - Cl in > 3 bonds path
1353000000 Max exchange energy for interaction C - Cl in > 3 bonds path
1354000000 Min e-e repulsion for interaction C - Cl in > 3 bonds path

1355000000	Max e-e repulsion for interaction C - Cl in > 3 bonds path
1356000000	Min e-n attraction for interaction C - Cl in > 3 bonds path
1357000000	Max e-n attraction for interaction C - Cl in > 3 bonds path
1358000000	Min n-n repulsion for interaction C - Cl in > 3 bonds path
1359000000	Max n-n repulsion for interaction C - Cl in > 3 bonds path
1360000000	Min coulumbic interaction for interaction C - Cl in > 3 bonds path
1361000000	Max coulumbic interaction for interaction C - Cl in > 3 bonds path
1362000000	Min total interaction for interaction C - Cl in > 3 bonds path
1363000000	Max total interaction for interaction C - Cl in > 3 bonds path
1364000000	[CD1H2]=*
1365000000	[CD2H](=*)-*
1367000000	[CD2H0](=*)=*
1368000000	[CD3H0](=*)(-*)-*
1369000000	[C,c
1370000000	[C,c
1371000000	[ND1H]=*
1372000000	[ND2H0](=*)-*
1373000000	[OD1H0]=*
1374000000	[PD4H0](=*)(-*)(-*)-*
1375000000	[SD1H0]=*
1376000000	[SD3H0](=*)(-*)-*
1377000000	[AsD4H0](=*)(-*)(-*)-*
1378000000	[SeD1H0]=*
1379000000	[SeD3H0](=*)(-*)-*
1380000000	[SeD4H0](=*)(=*)(-*)-*
1381000000	Number of 8-membered fused rings
1382000000	Number of 9-membered fused rings
1383000000	Number of 10-membered fused rings
1384000000	Number of 11-membered fused rings

1385000000	Number of 12-membered fused rings
1386000000	Number of >12-membered fused rings
1387000000	Number of rings (includes counts from fused rings)
1388000000	Number of 4-membered rings (includes counts from fused rings)
1389000000	Number of 5-membered rings (includes counts from fused rings)
1390000000	Number of 6-membered rings (includes counts from fused rings)
1391000000	Number of 7-membered rings (includes counts from fused rings)
1392000000	Number of 8-membered rings (includes counts from fused rings)
1393000000	Number of 9-membered rings (includes counts from fused rings)
1394000000	Number of 10-membered rings (includes counts from fused rings)
1395000000	Number of 11-membered rings (includes counts from fused rings)
1396000000	Number of 12-membered rings (includes counts from fused rings)
1397000000	Number of >12-membered rings (includes counts from fused rings)
1398000000	Number of rings containing heteroatoms (N, O, P, S, or halogens)
1399000000	Number of 3-membered rings containing heteroatoms (N, O, P, S, or halogens)
1400000000	Number of 4-membered rings containing heteroatoms (N, O, P, S, or halogens)
1401000000	Number of 5-membered rings containing heteroatoms (N, O, P, S, or halogens)
1402000000	Number of 6-membered rings containing heteroatoms (N, O, P, S, or halogens)
1403000000	Number of 7-membered rings containing heteroatoms (N, O, P, S, or halogens)
1404000000	Number of 8-membered rings containing heteroatoms (N, O, P, S, or halogens)
1405000000	Number of 9-membered rings containing heteroatoms (N, O, P, S, or halogens)
1406000000	Number of 10-membered rings containing heteroatoms (N, O, P, S, or halogens)
1407000000	Number of 11-membered rings containing heteroatoms (N, O, P, S, or halogens)
1408000000	Number of 12-membered rings containing heteroatoms (N, O, P, S, or halogens)
1409000000	Number of >12-membered rings containing heteroatoms (N, O, P, S, or halogens)
1410000000	Number of fused rings containing heteroatoms (N, O, P, S, or halogens)
1411000000	Number of 4-membered fused rings containing heteroatoms (N, O, P, S, or halogens)
1412000000	Number of 5-membered fused rings containing heteroatoms (N, O, P, S, or halogens)
1413000000	Number of 6-membered fused rings containing heteroatoms (N, O, P, S, or halogens)

1414000000	Number of 7-membered fused rings containing heteroatoms (N, O, P, S, or halogens)
1415000000	Number of 8-membered fused rings containing heteroatoms (N, O, P, S, or halogens)
1416000000	Number of 9-membered fused rings containing heteroatoms (N, O, P, S, or halogens)
1417000000	Number of 10-membered fused rings containing heteroatoms (N, O, P, S, or halogens)
1418000000	Number of 11-membered fused rings containing heteroatoms (N, O, P, S, or halogens)
1419000000	Number of 12-membered fused rings containing heteroatoms (N, O, P, S, or halogens)
1420000000	Number of >12-membered fused rings containing heteroatoms (N, O, P, S, or halogens)
1421000000	Number of rings (includes counts from fused rings) containing heteroatoms (N, O, P, S, or halogens)
1422000000	Number of 4-membered rings (includes counts from fused rings) containing heteroatoms (N, O, P, S, or halogens)
1423000000	Number of 5-membered rings (includes counts from fused rings) containing heteroatoms (N, O, P, S, or halogens)
1424000000	Number of 6-membered rings (includes counts from fused rings) containing heteroatoms (N, O, P, S, or halogens)
1425000000	Number of 7-membered rings (includes counts from fused rings) containing heteroatoms (N, O, P, S, or halogens)
1426000000	Number of 8-membered rings (includes counts from fused rings) containing heteroatoms (N, O, P, S, or halogens)
1427000000	Number of 9-membered rings (includes counts from fused rings) containing heteroatoms (N, O, P, S, or halogens)
1428000000	Number of 10-membered rings (includes counts from fused rings) containing heteroatoms (N, O, P, S, or halogens)
1429000000	Number of 11-membered rings (includes counts from fused rings) containing heteroatoms (N, O, P, S, or halogens)
1430000000	Number of 12-membered rings (includes counts from fused rings) containing heteroatoms (N, O, P, S, or halogens)
1431000000	Number of >12-membered rings (includes counts from fused rings) containing heteroatoms (N, O, P, S, or halogens)
