# Supplementary material for: Antiproliferative Activity Predictor: A New Reliable In Silico Tool for Drug Response Prediction against NCI60 Panel
Source: Int J Mol Sci. 2022 Nov 19;23(22):14374. doi: 10.3390/ijms232214374 (PMC9694168; doi:10.3390/ijms232214374)
Supplement: Supplementary file 1 [file ijms-23-14374-s001.zip › S12.pdf]

Chemistry

All solvent and reagents were used as received, unless otherwise stated. Melting points were determined on a hot-stage apparatus. ^{1}H-NMR and ^{13}C-NMR spectra were recorded at indicated frequencies, residual solvent peak was used as reference. Chromatography was performed by using silica gel (0.040–0.063 mm) and mixtures of ethyl acetate and petroleum ether (fraction boiling in the range of 40–60 °C) in various ratios (v/v). All solvent and reagents were used as received.

Compounds **1a-b,d-o** [1], **2a-c** [1], **2d** [2], **3a-g** [1] and **3h** [2] were prepared as previously reported. Characterization data are comparable with those reported in the literature. Compounds **1c** and **2e** were prepared adapting previously reported methods.

General procedure for the preparation of Cinnamils 1a-o.

Cinnamils **1** were prepared according to the known literature procedure.[1] In a 50 mL round bottom flask to a solution of diacetyl **5** (2.15 g, 25 mmol) in 10 mL methanol were added aldehyde **4a-o** (100 mmol, 4 equiv), acetic acid (0.03 equiv) and piperidine (0.03 equiv). The reaction mixture was refluxed at 83°C for 3h with stirring. The reaction mixture was cooled to room temperature, solvent was removed and cooled in an ice bath and the precipitate formed was filtered, washed with cold methanol and dried.

(1*E*,5*E*)-1,6-bis(3,4-dimethoxyphenyl)-1,5-hexadiene-3,4-dione 1a: Yield: 11%. m.p.= 162-163°C. ^{1}H-NMR (300 MHz, $CDCl_3$) δ: 7.84 (d, *J*= 15.9 Hz, 2H, =C*H*-), 7.37 (d, *J*= 15.9 Hz, 2H, =C*H*-), 7.27-7.20 (m, 4H, Ar), 6.91 (d, *J*= 8.1 Hz, 2H, Ar), 3.96 (s, 6H, OCH_3), 3.95 (s, 6H, OCH_3).

(1*E*,5*E*)-1,6-bis(4-methylphenyl)-1,5-hexadiene-3,4-dione 1b: Yield: 9%. m.p.= 185-186°C. ^{1}H-NMR (300 MHz, $CDCl_3$) δ: 7.85 (d, *J*= 16.3 Hz, 2H, =C*H*-), 7.57 (d, *J*= 7.9 Hz, 4H, Ar), 7.43 (d, *J*= 16.3 Hz, 2H, =C*H*-), 7.24 (d, *J*= 7.9 Hz, 4H, Ar), 2.41 (s, 6H, CH_3).

(1*E*,5*E*)-1,6-bis(4-benzyloxy-3-methoxyphenyl)-1,5-hexadiene-3,4-dione 1c: Yield: 18%. m.p.= 191-192°C. ^{1}H-NMR (300 MHz, $CDCl_3$) δ: 7.80 (d, *J*= 15.9 Hz, 2H, =C*H*-), 7.47-7.32 (m, 12H, Ar), 7.20-7.16 (m, 4H, Ar), 6.91 (d, *J*= 8.5 Hz, 2H, Ar), 5.22 (s, 4H, OCH_2), 3.96 (s, 6H, OCH_3). ^{13}C-NMR (75 MHz, $CDCl_3$) δ: 189.98, 152.08, 150.62, 148.46, 137.09, 129.36, 128.78, 127.91, 124.82, 118.46, 114.10, 111.38, 71.60, 56.80.

(1*E*,5*E*)-1,6-bis(4-methoxyphenyl)-1,5-hexadiene-3,4-dione 1d: Yield: 21%. m.p.= 170-171°C. ^{1}H-NMR (300 MHz, $CDCl_3$) δ: 7.83 (d, *J*= 15.9 Hz, 2H, =C*H*-), 7.62 (d, *J*= 9.0 Hz, 4H, Ar), 7.34 (d, *J*= 15.9 Hz, 2H, =C*H*-), 6.94 (d, *J*= 9.0 Hz, 4H, Ar), 3.86 (s, 6H, OCH_3).

(1*E*,5*E*)-1,6-diphenyl-1,5-hexadiene-3,4-dione 1e: Yield: 10%. m.p.= 167-168°C. ^{1}H-NMR (300 MHz, $CDCl_3$) δ: 7.88 (d, *J*= 16.0 Hz, 2H, =C*H*-), 7.67 (dd, *J*= 7.9, 2.0 Hz, 4H, Ar), 7.49 (d, *J*= 16.0 Hz, 2H, =C*H*-), 7.46–7.40 (m, 6H, Ar).

(1*E*,5*E*)-1,6-bis(4-fluorophenyl)hexa-1,5-diene-3,4-dione 1f: Yield: 11%. m.p.= 191-192°C. ^{1}H-NMR (300 MHz, $CDCl_3$) δ: 7.84 (d, *J*= 16.2 Hz, 2H, =C*H*-), 7.61-7.63 (m, 4H, Ar), 7.42 (d, *J*= 16.2 Hz, 2H, =C*H*-), 7.17-7.08 (m, 4H, Ar).

(*1E,5E*)-1,6-bis(4-(dimethylamino)phenyl)hexa-1,5-diene-3,4-dione 1g: Yield: 13%. m.p.= 247-248°C. ^{1}H-NMR (250MHz, $CDCl_3$) δ: 3.07 (s, 12H, NCH_3), 6,70 (d, 4H, *J*= 8.8 Hz, Ar), 7.24 (d, 2H, *J*=16.3 Hz, =C*H*-), 7.56 (d, 4H, *J*= 8.8, Ar), 7.80 (d, 2H, *J*= 16.3 Hz, =C*H*-).

(*1E,5E*)-1,6-bis(3,4-difluorophenyl)hexa-1,5-diene-3,4-dione 1i: Yield: 17%. m.p.= 218-220°C. ^{1}H-NMR (250MHz, $CDCl_3$) δ: 7.22-7.29 (m, 2H, Ar), 7.41-7.55 (m, 6H, =C*H*- + Ar), 7.79 (d, 2H, *J*= 15 Hz, =C*H*-).

(*1E,5E*)-1,6-bis(2-chlorophenyl)hexa-1,5-diene-3,4-dione 1k: Yield: 11%. m.p.= 137-138°C. ^{1}H-NMR (250MHz, $CDCl_3$) δ: 7.27-7.56 (m, 8H, =C*H*- + Ar), 7.79-7.93 (m, 2H, Ar), 8.34 (d, 2H, *J*= 16.3 Hz, =C*H*-).

(*1E,5E*)-1,6-bis(3-methoxyphenyl)hexa-1,5-diene-3,4-dione 1l: Yield: 18%. m.p.= 101-102°C. ^{1}H-NMR (250MHz, $CDCl_3$) δ 3.87 (s, 6H, OCH_3), 6.99-7.03 (m, 2H Ar), 7.18 (s, 2H, Ar), 7.24-7.28 (m, 2H, Ar), 7.36 (t, 2H, *J*= 7.7 Hz, Ar), 7.48 (d, 2H, *J*= 15 Hz, =C*H*-), 7.85 (d, 2H, *J*= 15 Hz, =C*H*-).

(*1E,5E*)-1,6-bis(3-methoxy-4-(methoxymethoxy)phenyl)hexa-1,5-diene-3,4-dione 1m: Yield: 12%. m.p.= 165-166°C. ^{1}H-NMR (250MHz, $CDCl_3$) δ: 3.53 (s, 6H, OCH_3), 3.96 (s, 6H, OCH_3), 5.31 (s, 4H, OCH_2O), 7.20-7.25 (m, 6H, Ar), 7.39 (d, 2H, *J*= 16.0 Hz, =C*H*-), 7.83 (d, 1H, *J*= 16.0 Hz, =C*H*-).

(*1E,5E*)-1,6-di(benzo[d][1,3]dioxol-5-yl)hexa-1,5-diene-3,4-dione 1n: Yield: 9%. m.p.= 255-256°C. ^{1}H-NMR (250MHz, $CDCl_3$) δ: 6.05 (s, 4H, OCH_2O), 6.84-6.87 (m, 2H, Ar), 7.13-7.18 (m, 4H, *Ar*), 7.31 (d, 2H, *J*= 16.5 Hz, =C*H*-), 7.78 (d, 2H, *J*= 16.5 Hz, =C*H*-).

4,4'-((*1E,5E*)-3,4-dioxohexa-1,5-diene-1,6-diyl)dibenzoic acid 1o: Yield: 14%. m.p.= 236°C (dec.). ^{1}H-NMR (250MHz, DMSO-*d*$_6$) δ 7.59 (d, 2H, *J*= 17.5 Hz, =CH-), 7.90 (d, 2H, *J*= 17.5 Hz, =CH-), 7.99-8.21 (m, 8H, Ar).

Preparation of 1,2,4-oxadiazole 2a-e.

Amidoxime **7** (1 mmol), ethyl ester **6** (1.5 mmol) and K_2CO_3 (3 mmol) were mixed in a glass tube under solvent free conditions and heated at 110°C until complete fusion. The reaction was monitored until completion via TLC. The crude mixture was treated with water (50 mL) and extracted with ethyl

acetate (100 mL). The organic layer was dried over anhydrous Na_2SO_4, filtered, concentrated under reduced pressure, and purified using column chromatography.

3,5-bis(3,4-dimethoxystyryl)-1,2,4-oxadiazole 2a: Yield: 76%. m.p.= 123-125°C. ^{1}H-NMR (250MHz, $CDCl_3$) δ 3.94 (s, 3H, OCH_3), 3.96 (s, 9H, OCH_3), 6.87-6.99 (m, 3H, =C*H*- + Ar), 7.14-7.22 (m, 5H, Ar), 7.68 (d, 1H, *J*= 16.2 Hz, =C*H*-), 7.80 (d, 1H, *J*= 16.2 Hz, =C*H*-).

3,5-bis(4-methoxystyryl)-1,2,4-oxadiazole 2b: Yield: 85%. m.p.= 163-165°C. ^{1}H-NMR (250MHz, $CDCl_3$) δ 3.85 (s, 3H, OCH_3), 3.87 (s, 3H, OCH_3), 6.87-6.99 (m, 6H, =C*H*- + Ar), 7.52-7.58 (m, 4H, Ar), 7.68 (d, 1H, *J*= 16.2 Hz, =C*H*-), 7.81 (d, 1H, *J*= 16.5 Hz, =C*H*-).

3,5-distyryl-1,2,4-oxadiazole 2c: Yield: 82%. m.p.= 113-115°C. ^{1}H-NMR (250MHz, $CDCl_3$) δ 7.05 (d, 1H, *J*= 16.5 Hz, =C*H*-), 7.11 (d, 1H, *J*= 16.2 Hz, =C*H*-), 7.38-7.47 (m, 6H, Ar), 7.60-7.64 (m, 4H, Ar), 7.77 (d, 1H, *J*= 16.2 Hz, =C*H*-), 7.89 (d, 1H, *J*= 16.5 Hz, =C*H*-).

5-(3,4-dimethoxystyryl)-3-(3-chloro-4-methoxystyryl)-1,2,4-oxadiazole 2d: Yield 62%, m.p.= 176–177 °C. ^{1}H-NMR (300 MHz, $CDCl_3$) δ: 3.97 (s, 9H, OCH_3), 6.92 (d, 1H, *J*= 16.2 Hz, =C*H*–), 6.98 (d, 1H, *J*= 16 Hz, =C*H*–), 6.90–7.03 (m, 2H, Ar), 7.18 (d, 2H, *J*= 7.8 Hz, Ar), 7.50 (dd, J_1= 8.8 Hz, J_2= 2 Hz, 1H, Ar), 7.70 (d, 1H, *J*= 16.0 Hz, =C*H*–), 7.77 (d, 1H, *J*= 16.2 Hz, =C*H*–).

3,5-bis(4-dimethylaminostyryl)-1,2,4-oxadiazole 2e: Yield 67%, m.p.= 219–221 °C. ^{1}H-NMR (250MHz, $CDCl_3$) δ 3.02 (s, 6H, N(C*H*$_3$)$_2$), 3.05 (s, 6H, N(C*H*$_3$)$_2$), 6.70-6.73 (m, 4H, Ar), 6.78 (d, 1H, *J*= 16.0 Hz, =C*H*-), 6.86 (d, 1H, *J*= 16.0 Hz, =C*H*-), 7.46-7.51 (m, 4H, Ar), 7.65 (d, 1H, *J*= 16.0 Hz, =C*H*-), 7.75 (d, 1H, *J*= 16.0 Hz, =C*H*-). ^{13}C-NMR (62.5 MHz, $CDCl_3$) δ: 176.78, 163.52, 143.49, 142.99, 139.48, 133.12, 130.26, 129.48, 112.82, 112.68, 112.01, 108.99, 105.91, 105.59, 40.94, 40.82.

General method of preparation of 1,3,4-oxadiazole 3a-h

To a solution of cinnamic acid **8** (1.2 mmol) in acetonitrile (7 mL), HOBt (1.2 mmol) and EDC (1.2 mmol) were added. The resulting mixture was stirred for 2 h. Hydrazine hydrate (2 mmol) was successively added and was stirred again for 1 h. The resulting mixture was poured into 10% NaOH aq and extracted with ethyl acetate. The organic layer was dried over sodium sulfate, and concentrated for the next step. To a solution of **8** (1.05 mmol) in acetonitrile (10 mL), was added 1-hydroxybenzotriazole hydrate (1.05 mmol), diisopropylethylamine (1.05 mmol) and EDC (1.05 mmol) and the resulting mixture was stirred for 1.5 h. Previously obtained Hydrazide was added and was stirred again until disappearance of the starting products. The resulting mixture was poured into

10% NaOH aq and extracted with ethyl acetate. The combined organic layers dried over sodium sulfate and concentrated for the obtainment of diacylhydrazide. Diacylhydrazide in acetonitrile (15 mL) was stirred for 1 h with 4-toluensulfonyl chloride (3 mmol) and diisopropylethylamine (2 mmol). At the end of the reaction the crude mixture was concentrated *in vacuo* end treated with water and NaOH. The aqueous phase was extracted with dichloromethane, dried over sodium sulfate and concentrated *in vacuo* The resulting crude was purified by chromatography.

2,5-bis(3,4-difluorostyryl)-1,3,4-oxadiazole 3a: Yield: 67%. m.p.= 233-235°C. ^{1}H-NMR (300MHz, $CDCl_3$) δ: 6.98 (d, 2H, *J*= 16.2 Hz, =C*H*-), 7.19-7.43 (m, 6H, Ar), 7.53 (d, 2H, *J*= 16.2 Hz, =C*H*-).
2,5-bis(4-chlorostyryl)-1,3,4-oxadiazole 3b: Yield: 82%. m.p.= 229-230°C. ^{1}H-NMR (300MHz, $CDCl_3$) δ: 7.03 (d, 2H, *J*= 16.2 Hz, =C*H*-), 7.40 (d, 4H, *J*= 7 Hz, Ar), 7.51 (d, 4H, *J*= 7 Hz, Ar), 7.56 (d, 2H, *J*= 16.2 Hz, =C*H*-).

2,5-bis((*E*)-2-(benzo[d][1,3]dioxol-5-yl)vinyl)-1,3,4-oxadiazole 3c: Yield: 74%. m.p.= 222-224°C. ^{1}H-NMR (250MHz, $CDCl_3$) δ: 6.04 (s, 4H, OCH_2O), 6.85-6.91 (m, 4H, =C*H*- + Ar), 7.04-7.10 (m, 4H, Ar), 7.50 (d, 2H, *J*= 16.2 Hz, =C*H*-).
4-((*1E*)-2-(5-(4-(dimethylamino)styryl)-1,3,4-oxadiazol-2-yl)vinyl)-N,N-dimethylbenzenamine 3d: Yield: 59%. m.p.= 217-219°C. ^{1}H-NMR (250MHz, $CDCl_3$) δ: 3.05 (s, 12H, N$(CH_3)_2$), 6.73-6.87 (m, 6H, =C*H*- + Ar), 7.46-7.54 (m, 6H, =C*H*- + Ar).
2,5-bis(3,4-dimethoxystyryl)-1,3,4-oxadiazole 3e: Yield: 74%. m.p.= 139-140°C. ^{1}H-NMR (250MHz, $CDCl_3$) δ: 3.94 (s, 6H, OCH_3), 3.96 (s, 6H, OCH_3), 6.89-6.97 (m, 4H, =C*H*- + Ar), 7.11-7.17 (m, 4H, Ar), 7.54 (d, 2H, *J*= 16.3 Hz, =C*H*-).
2,5-bis(4-methoxystyryl)-1,3,4-oxadiazole 3f: Yield: 83%. m.p.= 190-192°C. ^{1}H-NMR (250MHz, $CDCl_3$) δ: 3.83 (s, 6H, OCH_3), 6.85-6.93 (m, 6H, =C*H*- + Ar), 7.48-7.55 (m, 6H, =C*H*- + Ar).

2,5-bis(3-methoxy-4-(methoxymethoxy)styryl)-1,3,4-oxadiazole 3g: Yield: 67%. m.p.= 128°C (dec.). ^{1}H-NMR (250MHz, $CDCl_3$) δ: 3.67 (s, 6H, OCH_3), 3.94 (s, 6H, OCH_3), 5.26 (s, 4H, OCH_2O), 6.84 (d, 2H, *J*= 16.4 Hz, =C*H*-), 7.13-7.19 (m, 6H, Ar), 7.54 (d, 2H, *J*= 16.4 Hz, =C*H*-).

2,5-bis(4-fluoro-3-methoxystyryl)-1,3,4-oxadiazole 3h: Yield: 76%. m.p. = 186–188 °C. ^{1}H-NMR (300 MHz, $CDCl_3$) δ: 3.97 (s, 6H, OCH_3), 6.99 (d, 2H, *J*= 16.5 Hz, =CH–), 7.12–7.20 (m, 6H, Ar), 7.55 (d, 2H, *J*= 16.5 Hz, =CH–).

1. Lo Cascio, F.; Puangmalai, N.; Ellsworth, A.; Bucchieri, F.; Pace, A.; Palumbo Piccionello, A.; Kayed, R. Toxic Tau Oligomers Modulated by Novel Curcumin Derivatives. *Sci Rep* **2019**, *9*, 19011, doi:10.1038/s41598-019-55419-w.
2. Battisti, A.; Palumbo Piccionello, A.; Sgarbossa, A.; Vilasi, S.; Ricci, C.; Ghetti, F.; Spinozzi, F.; Marino Gammazza, A.; Giacalone, V.; Martorana, A.; et al. Curcumin-like compounds designed to modify amyloid beta peptide aggregation patterns. *RSC Advances* **2017**, *7*, 31714-31724, doi:10.1039/c7ra05300b.
