# Supplementary material for: Antiproliferative Activity Predictor: A New Reliable In Silico Tool for Drug Response Prediction against NCI60 Panel
Source: Int J Mol Sci. 2022 Nov 19;23(22):14374. doi: 10.3390/ijms232214374 (PMC9694168; doi:10.3390/ijms232214374)

Developmental Therapeutics Program
One Dose Mean Graph

NSC: D-785543 / 1	Conc: 1.00E-5 Molar	Test Date: Jul 20, 2015
Experiment ID: 1507OS27		Report Date: Sep 22, 2015

Panel/Cell Line	Growth Percent
Leukemia	
HL-60(TB)	12.07
K-562	14.77
MOLT-4	20.25
RPMI-8226	33.96
SR	11.58
Non-Small Cell Lung Cancer	
A549/ATCC	31.99
EKVX	36.85
HOP-62	35.27
HOP-92	20.40
NCI-H226	63.49
NCI-H23	25.37
NCI-H460	15.60
NCI-H522	9.91
Colon Cancer	
COLO 205	22.05
HCC-2998	54.91
HCT-116	4.83
HCT-15	21.55
HT29	-7.70
KM12	33.75
SW-620	18.08
CNS Cancer	
SF-268	41.89
SF-295	12.48
SF-539	-11.79
SNB-19	14.80
SNB-75	38.38
U251	16.20
Melanoma	
LOX IMVI	4.41
MALME-3M	57.12
M14	5.44
MDA-MB-435	10.38
SK-MEL-2	20.29
SK-MEL-28	30.55
SK-MEL-5	2.81
UACC-257	61.40
UACC-62	9.17
Ovarian Cancer	
IGROV1	22.51
OVCAR-3	17.60
OVCAR-4	57.56
OVCAR-5	41.26
OVCAR-8	42.08
NCI/ADR-RES	13.65
SK-OV-3	34.11
Renal Cancer	
786-0	12.80
A498	31.42
ACHN	45.37
CAKI-1	51.37
RXF 393	27.44
SN12C	22.09
TK-10	45.54
UO-31	37.36
Prostate Cancer	
PC-3	31.46
DU-145	49.21
Breast Cancer	
MCF7	22.83
MDA-MB-231/ATCC	17.75
HS 578T	43.58
BT-549	25.53
T-47D	19.54
MDA-MB-468	19.02
Mean	26.27
Delta	38.06
Range	75.28

Mean Growth Percent - Growth Percent

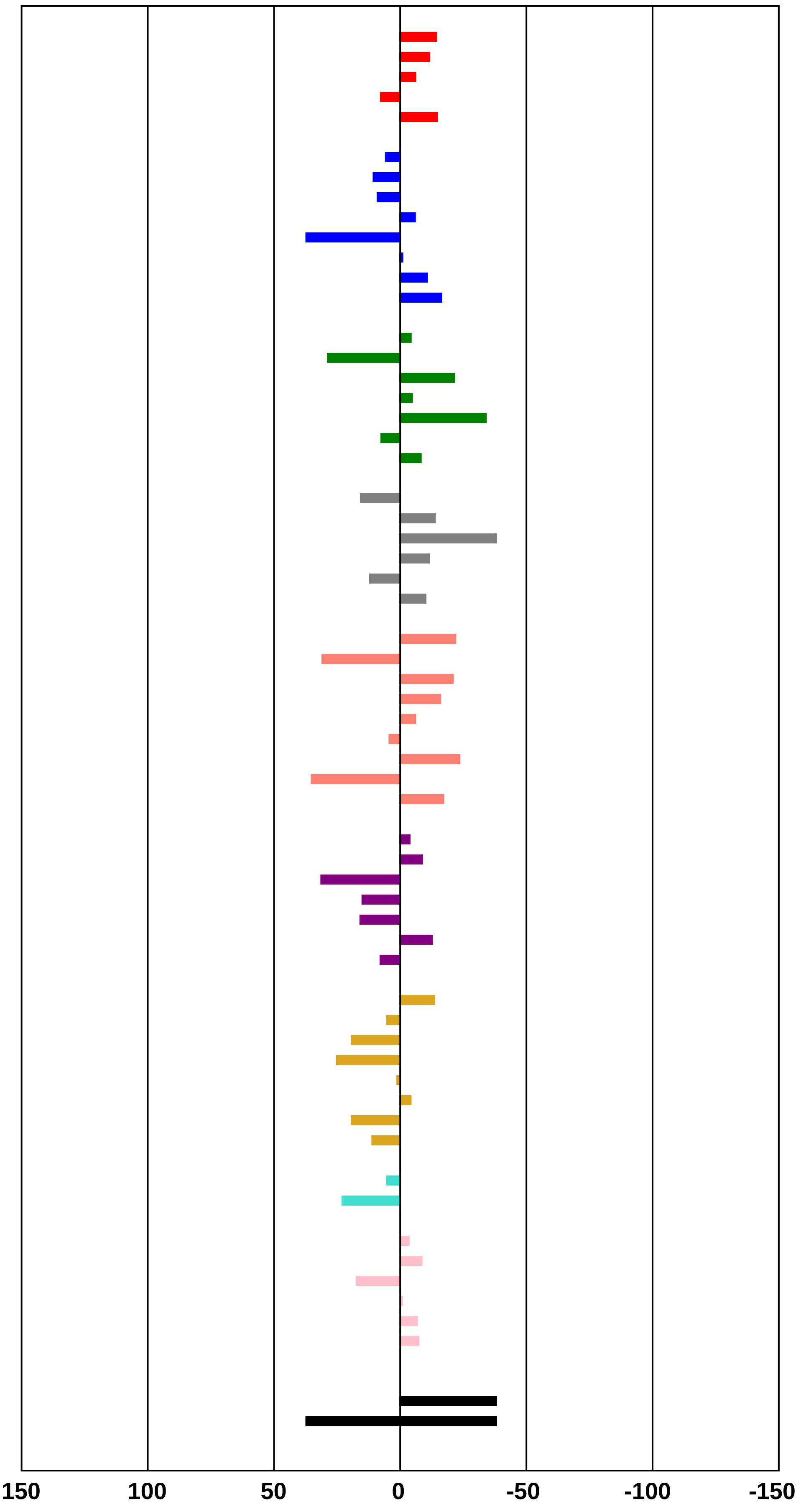

Supplement: Supplementary file 1 [file ijms-23-14374-s001.zip › S13 .zip!/Compound 3e - NCI OneDoseData.pdf]
