# Supplementary material for: Antiproliferative Activity Predictor: A New Reliable In Silico Tool for Drug Response Prediction against NCI60 Panel
Source: Int J Mol Sci. 2022 Nov 19;23(22):14374. doi: 10.3390/ijms232214374 (PMC9694168; doi:10.3390/ijms232214374)

# Developmental Therapeutics Program

## One Dose Mean Graph

**NSC:** D-785542 / 1 | **Conc:** 1.00E-5 Molar | **Test Date:** Jul 20, 2015

**Experiment ID:** 1507OS27 | **Report Date:** Sep 22, 2015

| Panel/Cell Line | Growth Percent |
|---|---|
| **Leukemia** | |
| HL-60(TB) | 88.74 |
| K-562 | 84.68 |
| MOLT-4 | 76.85 |
| RPMI-8226 | 72.43 |
| SR | 64.66 |
| **Non-Small Cell Lung Cancer** | |
| A549/ATCC | 81.61 |
| EKVX | 81.86 |
| HOP-62 | 99.84 |
| HOP-92 | 83.84 |
| NCI-H226 | 76.29 |
| NCI-H23 | 82.37 |
| NCI-H460 | 96.21 |
| NCI-H522 | 73.11 |
| **Colon Cancer** | |
| COLO 205 | 95.21 |
| HCC-2998 | 108.14 |
| HCT-116 | 71.33 |
| HCT-15 | 75.77 |
| HT29 | 56.32 |
| KM12 | 94.58 |
| SW-620 | 104.31 |
| **CNS Cancer** | |
| SF-268 | 106.66 |
| SF-295 | 97.35 |
| SF-539 | 88.71 |
| SNB-19 | 109.72 |
| SNB-75 | 95.07 |
| U251 | 91.63 |
| **Melanoma** | |
| LOX IMVI | 97.63 |
| MALME-3M | 99.27 |
| M14 | 104.95 |
| MDA-MB-435 | 96.60 |
| SK-MEL-2 | 94.95 |
| SK-MEL-28 | 104.53 |
| SK-MEL-5 | 71.45 |
| UACC-257 | 113.80 |
| UACC-62 | 87.44 |
| **Ovarian Cancer** | |
| IGROV1 | 90.46 |
| OVCAR-3 | 110.37 |
| OVCAR-4 | 80.92 |
| OVCAR-5 | 80.09 |
| OVCAR-8 | 102.39 |
| NCI/ADR-RES | 92.19 |
| SK-OV-3 | 110.61 |
| **Renal Cancer** | |
| 786-0 | 92.33 |
| A498 | 92.21 |
| ACHN | 99.78 |
| CAKI-1 | 88.44 |
| RXF 393 | 112.32 |
| SN12C | 88.25 |
| TK-10 | 97.49 |
| UO-31 | 84.05 |
| **Prostate Cancer** | |
| PC-3 | 78.94 |
| DU-145 | 98.83 |
| **Breast Cancer** | |
| MCF7 | 90.07 |
| MDA-MB-231/ATCC | 107.24 |
| HS 578T | 103.31 |
| BT-549 | 84.86 |
| T-47D | 61.61 |
| MDA-MB-468 | 86.46 |
| Mean | 90.71 |
| Delta | 34.39 |
| Range | 57.48 |

**Mean Growth Percent - Growth Percent**

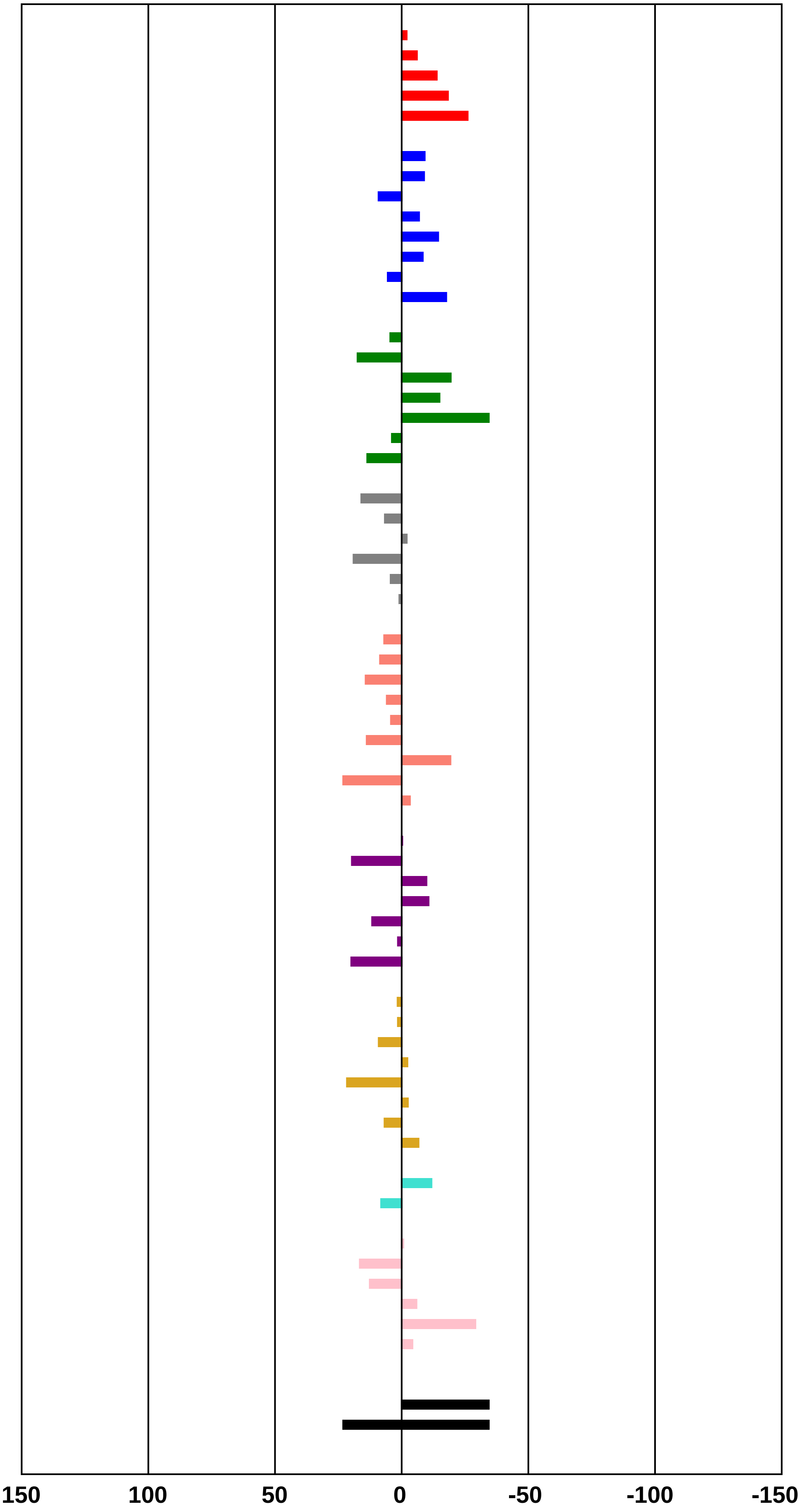

Supplement: Supplementary file 1 [file ijms-23-14374-s001.zip › S13 .zip!/Compound 2a - NCI OneDoseData.pdf]
