# Supplementary material for: Antiproliferative Activity Predictor: A New Reliable In Silico Tool for Drug Response Prediction against NCI60 Panel
Source: Int J Mol Sci. 2022 Nov 19;23(22):14374. doi: 10.3390/ijms232214374 (PMC9694168; doi:10.3390/ijms232214374)

Developmental Therapeutics Program

One Dose Mean Graph

**NSC:** D-785540 / 1	**Conc:** 1.00E-5 Molar
**Test Date:** Jul 20, 2015	**Experiment ID:** 1507OS27
**Report Date:** Sep 22, 2015	

Panel/Cell Line	Growth Percent
Leukemia	
HL-60(TB)	100.42
K-562	94.09
MOLT-4	99.58
RPMI-8226	95.62
SR	92.14
Non-Small Cell Lung Cancer	
A549/ATCC	80.40
EKVX	93.33
HOP-62	91.54
HOP-92	110.22
NCI-H226	108.87
NCI-H23	89.43
NCI-H460	107.91
NCI-H522	82.08
Colon Cancer	
COLO 205	107.16
HCC-2998	90.64
HCT-116	69.01
HCT-15	104.88
HT29	87.34
KM12	106.85
SW-620	104.97
CNS Cancer	
SF-268	110.01
SF-295	102.47
SF-539	100.23
SNB-19	104.76
SNB-75	93.29
U251	97.66
Melanoma	
LOX IMVI	96.25
MALME-3M	100.07
M14	102.18
MDA-MB-435	102.25
SK-MEL-2	101.04
SK-MEL-28	97.25
SK-MEL-5	105.00
UACC-257	102.33
UACC-62	98.92
Ovarian Cancer	
IGROV1	96.34
OVCAR-3	116.68
OVCAR-4	101.22
OVCAR-5	92.19
OVCAR-8	105.45
NCI/ADR-RES	97.85
SK-OV-3	100.45
Renal Cancer	
786-0	101.16
A498	105.68
ACHN	98.78
CAKI-1	96.42
RXF 393	113.89
SN12C	104.39
TK-10	92.28
UO-31	89.91
Prostate Cancer	
PC-3	91.84
DU-145	111.40
Breast Cancer	
MCF7	86.34
MDA-MB-231/ATCC	98.20
HS 578T	96.02
BT-549	108.75
T-47D	97.26
MDA-MB-468	111.16
Mean	99.03
Delta	30.02
Range	47.67

Mean Growth Percent - Growth Percent

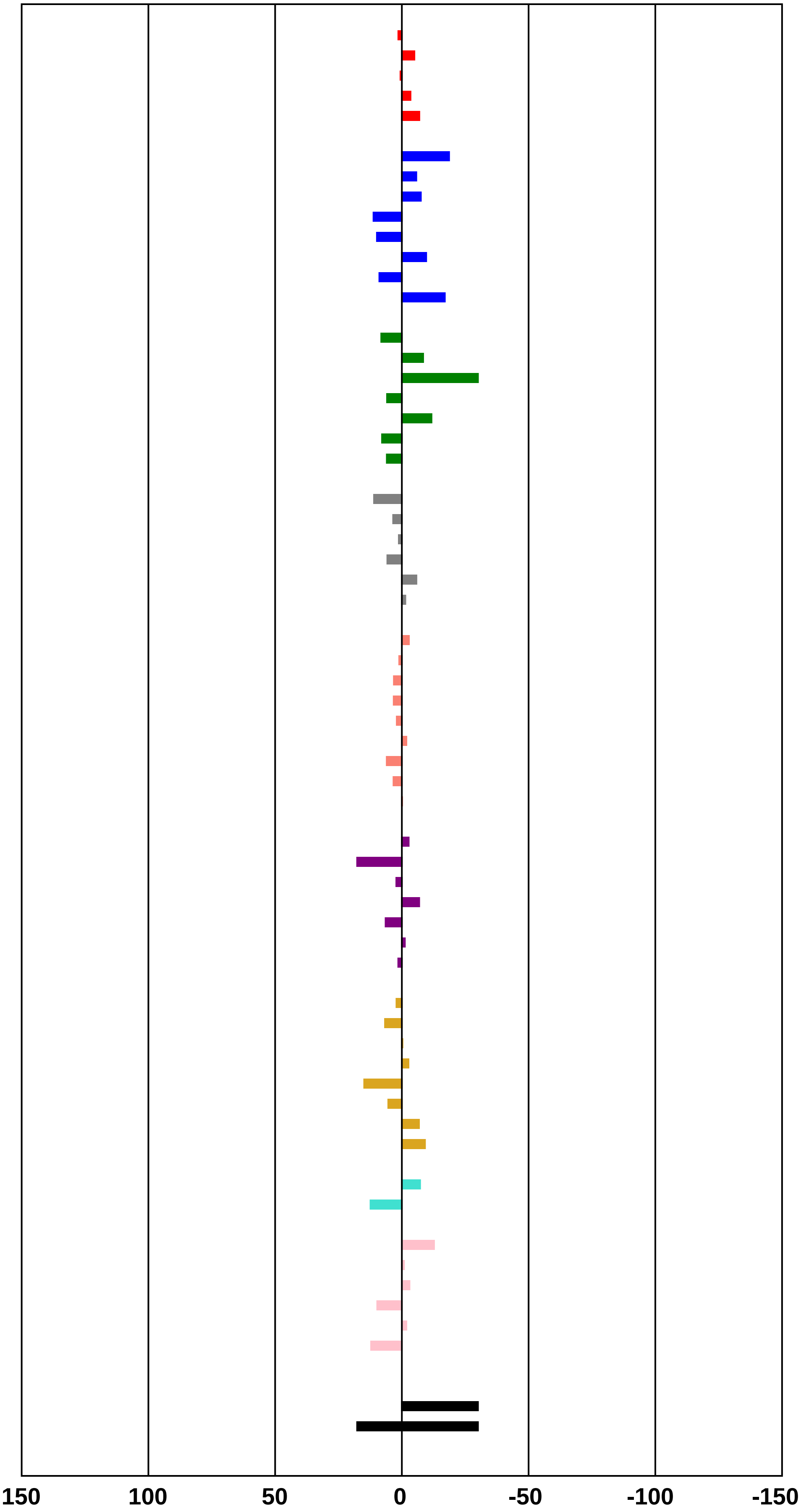

Supplement: Supplementary file 1 [file ijms-23-14374-s001.zip › S13 .zip!/Compound 1c - NCI OneDoseData.pdf]
