# Supplementary material for: Antiproliferative Activity Predictor: A New Reliable In Silico Tool for Drug Response Prediction against NCI60 Panel
Source: Int J Mol Sci. 2022 Nov 19;23(22):14374. doi: 10.3390/ijms232214374 (PMC9694168; doi:10.3390/ijms232214374)

Developmental Therapeutics Program

One Dose Mean Graph

**NSC:** D-785539 / 1	**Conc:** 1.00E-5 Molar
**Test Date:** Jul 20, 2015	**Experiment ID:** 1507OS27
**Report Date:** Sep 22, 2015	

Panel/Cell Line	Growth Percent
Leukemia	
HL-60(TB)	93.30
K-562	95.25
MOLT-4	91.81
RPMI-8226	45.15
SR	68.03
Non-Small Cell Lung Cancer	
A549/ATCC	86.21
EKVX	92.66
HOP-62	109.07
HOP-92	108.23
NCI-H226	106.59
NCI-H23	95.42
NCI-H460	104.32
NCI-H522	81.66
Colon Cancer	
COLO 205	110.34
HCC-2998	110.01
HCT-116	-36.84
HCT-15	95.49
HT29	91.36
KM12	93.80
SW-620	100.42
CNS Cancer	
SF-268	108.22
SF-295	100.74
SF-539	96.24
SNB-19	102.76
SNB-75	90.38
U251	92.98
Melanoma	
LOX IMVI	89.25
MALME-3M	96.78
M14	95.18
MDA-MB-435	76.95
SK-MEL-2	105.32
SK-MEL-28	108.04
SK-MEL-5	105.87
UACC-257	103.29
UACC-62	96.72
Ovarian Cancer	
IGROV1	94.41
OVCAR-3	112.03
OVCAR-4	98.64
OVCAR-5	97.82
OVCAR-8	108.56
NCI/ADR-RES	103.34
SK-OV-3	108.68
Renal Cancer	
786-0	96.94
A498	100.68
ACHN	103.97
CAKI-1	94.75
RXF 393	117.28
SN12C	104.79
TK-10	91.99
UO-31	89.74
Prostate Cancer	
PC-3	92.20
DU-145	111.88
Breast Cancer	
MCF7	23.84
MDA-MB-231/ATCC	106.06
HS 578T	92.59
BT-549	95.26
T-47D	92.38
MDA-MB-468	110.47
Mean	94.13
Delta	130.97
Range	154.12

Mean Growth Percent - Growth Percent

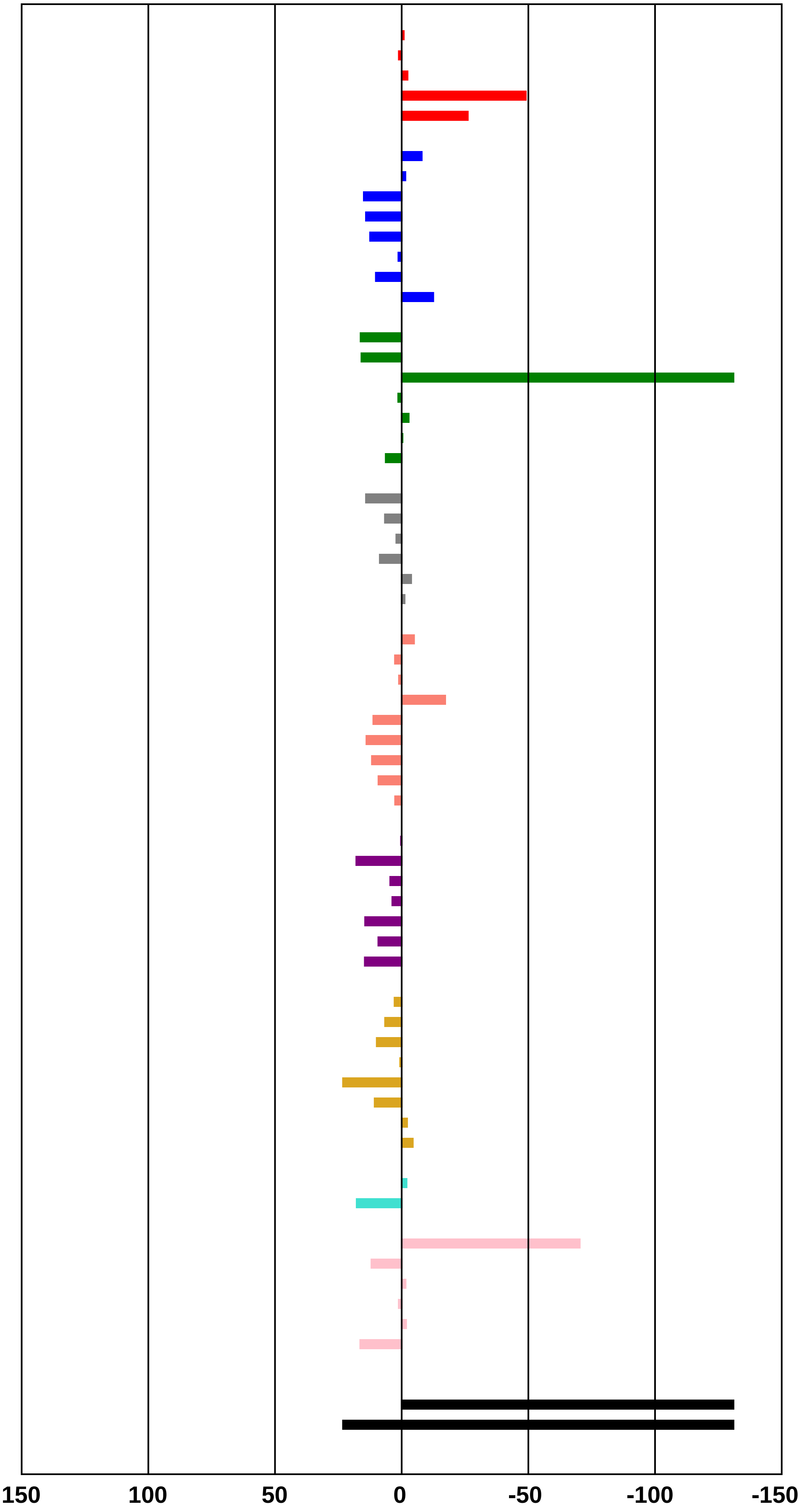

Supplement: Supplementary file 1 [file ijms-23-14374-s001.zip › S13 .zip!/Compound 1b - NCI OneDoseData.pdf]
