# Supplementary material for: Antiproliferative Activity Predictor: A New Reliable In Silico Tool for Drug Response Prediction against NCI60 Panel
Source: Int J Mol Sci. 2022 Nov 19;23(22):14374. doi: 10.3390/ijms232214374 (PMC9694168; doi:10.3390/ijms232214374)

Developmental Therapeutics Program

One Dose Mean Graph

**NSC:** D-785541 / 1	**Conc:** 1.00E-5 Molar	**Test Date:** Jul 20, 2015
**Experiment ID:** 1507OS27		**Report Date:** Sep 22, 2015

Panel/Cell Line	Growth Percent
Leukemia	
HL-60(TB)	35.40
K-562	7.11
MOLT-4	23.43
RPMI-8226	3.50
SR	4.78
Non-Small Cell Lung Cancer	
A549/ATCC	69.89
EKVX	62.59
HOP-62	93.17
HOP-92	108.64
NCI-H226	38.87
NCI-H23	21.61
NCI-H460	51.66
NCI-H522	47.85
Colon Cancer	
COLO 205	-20.85
HCC-2998	21.93
HCT-116	-74.85
HCT-15	-3.56
HT29	-35.20
KM12	-4.31
SW-620	4.43
CNS Cancer	
SF-268	13.17
SF-295	73.81
SF-539	6.56
SNB-19	33.06
SNB-75	64.88
U251	6.40
Melanoma	
LOX IMVI	-66.41
MALME-3M	54.45
M14	-10.50
MDA-MB-435	12.23
SK-MEL-2	96.60
SK-MEL-28	-9.43
SK-MEL-5	77.86
UACC-257	74.38
UACC-62	-10.83
Ovarian Cancer	
IGROV1	10.13
OVCAR-3	3.90
OVCAR-4	61.86
OVCAR-5	60.49
OVCAR-8	19.51
NCI/ADR-RES	71.71
SK-OV-3	97.78
Renal Cancer	
786-0	3.43
A498	72.88
ACHN	-0.68
CAKI-1	84.27
RXF 393	-63.27
SN12C	2.82
TK-10	68.56
UO-31	0.57
Prostate Cancer	
PC-3	43.62
DU-145	22.49
Breast Cancer	
MCF7	4.02
MDA-MB-231/ATCC	2.99
HS 578T	31.48
BT-549	14.85
T-47D	35.84
MDA-MB-468	20.08
Mean	26.58
Delta	101.43
Range	183.49

Mean Growth Percent - Growth Percent

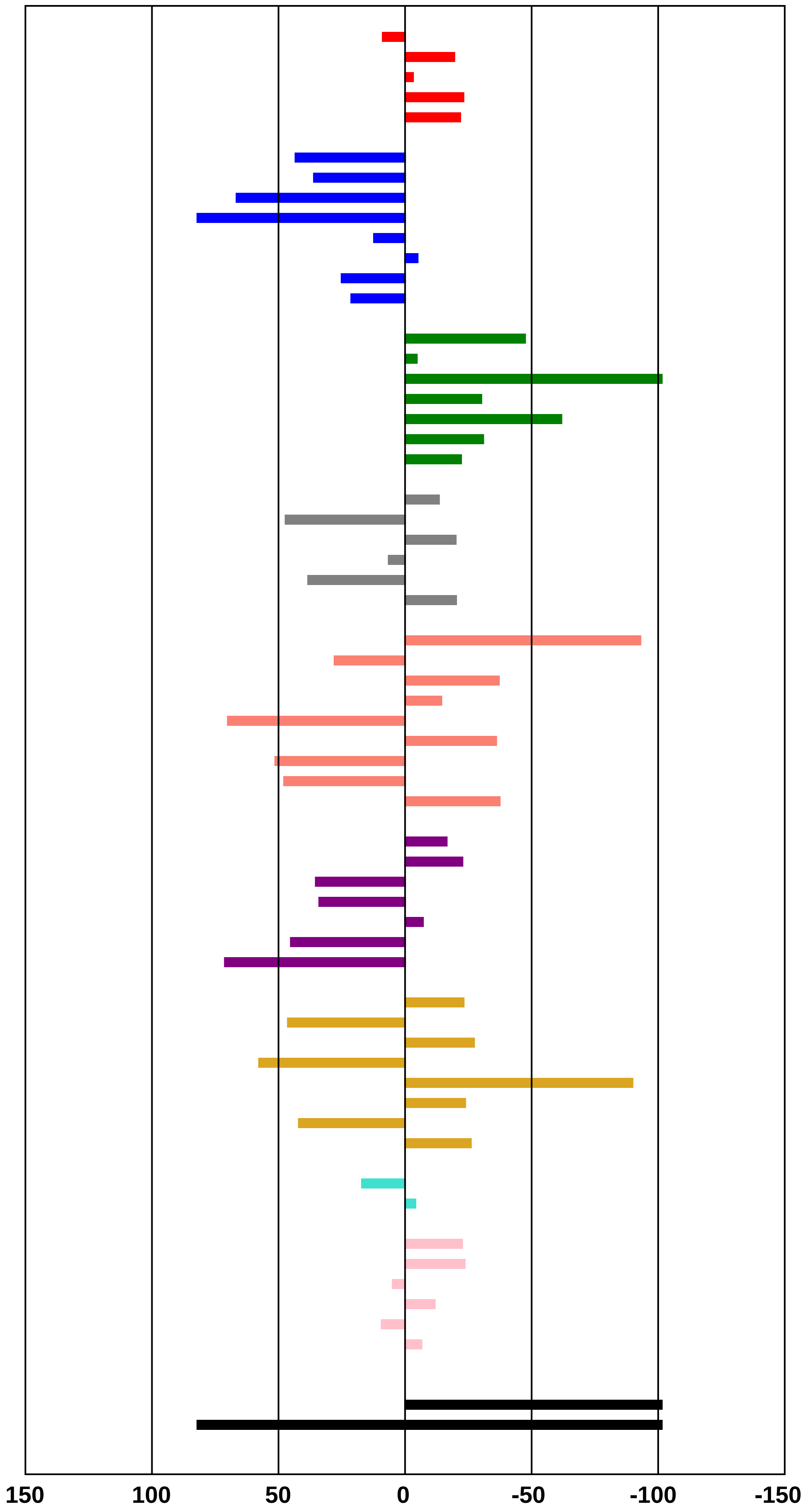

Supplement: Supplementary file 1 [file ijms-23-14374-s001.zip › S13 .zip!/Compound 1a- NCI OneDoseData.pdf]
