# Supplementary material for: Antiproliferative Activity Predictor: A New Reliable In Silico Tool for Drug Response Prediction against NCI60 Panel
Source: Int J Mol Sci. 2022 Nov 19;23(22):14374. doi: 10.3390/ijms232214374 (PMC9694168; doi:10.3390/ijms232214374)

National Cancer Institute Developmental Therapeutics Program<br>Dose Response Curves	NSC: D - 785543 / 1	SSPL: 0JPC	EXP. ID: 1508NS40
	Report Date: September 22, 2015	Test Date: August 17, 2015	

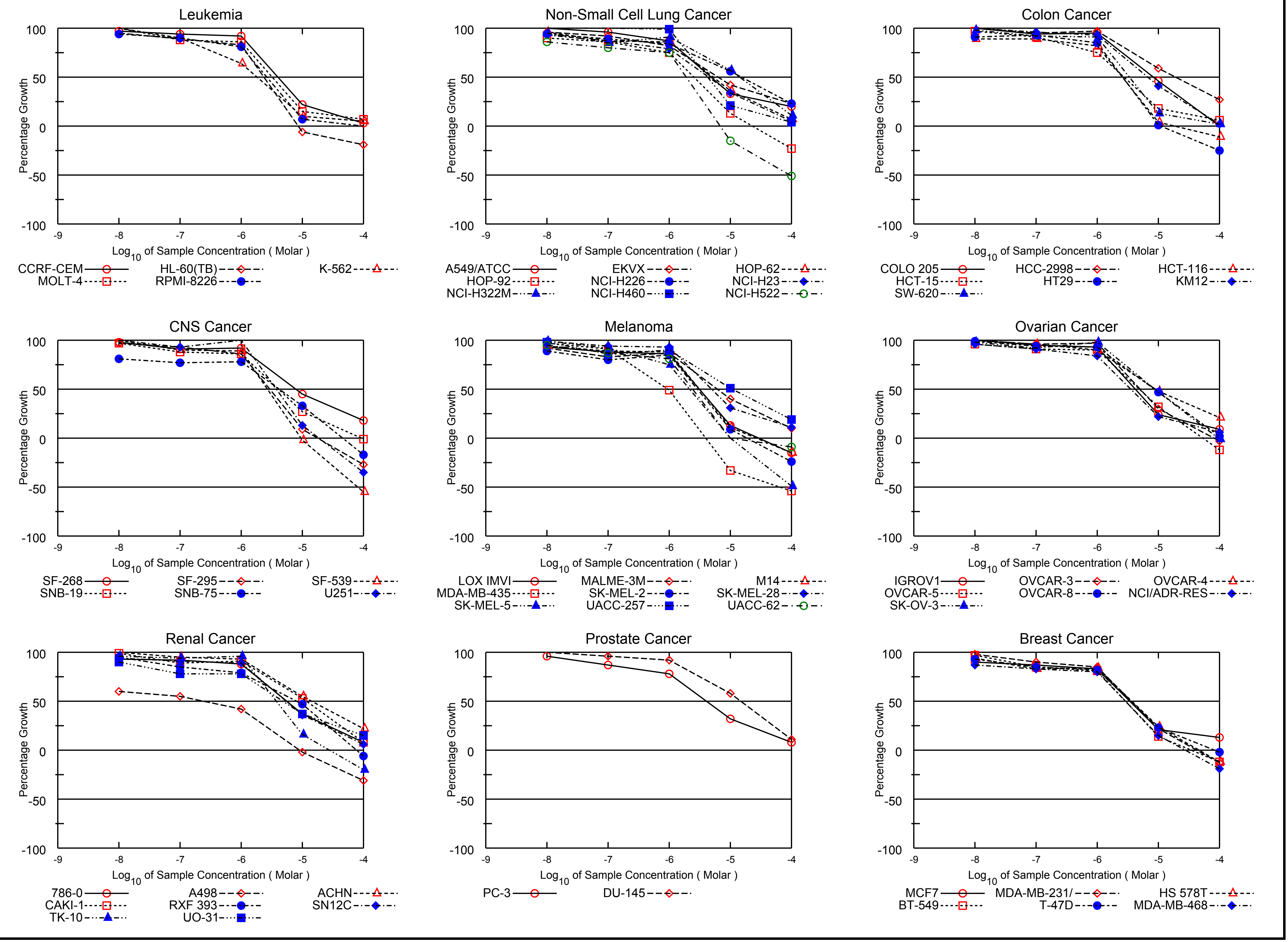

National Cancer Institute Developmental Therapeutics Program
In-Vitro Testing Results

NSC : D - 785543 / 1	Experiment ID : 1508NS40	Test Type : 08	Units : Molar
Report Date : September 22, 2015	Test Date : August 17, 2015	QNS :	MC :
COMI : NV2156	Stain Reagent : SRB Dual-Pass Related	SSPL : 0JPC	

			Log10 Concentration												
	Time		Mean Optical Densities					Percent Growth							
Panel/Cell Line	Zero	Ctrl	-8.0	-7.0	-6.0	-5.0	-4.0	-8.0	-7.0	-6.0	-5.0	-4.0	GI50	TGI	LC50
**Leukemia**															
CCRF-CEM	0.775	3.093	3.018	2.952	2.903	1.284	0.846	97	94	92	22	3	3.97E-6	> 1.00E-4	> 1.00E-4
HL-60(TB)	0.866	3.244	3.128	2.974	2.842	0.816	0.706	95	89	83	-6	-19	2.36E-6	8.60E-6	> 1.00E-4
K-562	0.299	2.258	2.259	2.067	1.559	0.490	0.395	100	90	64	10	5	1.83E-6	> 1.00E-4	> 1.00E-4
MOLT-4	0.563	2.584	2.577	2.340	2.311	0.868	0.700	100	88	86	15	7	3.24E-6	> 1.00E-4	> 1.00E-4
RPMI-8226	0.922	2.859	2.747	2.672	2.500	1.058	0.929	94	90	81	7	.	2.65E-6	> 1.00E-4	> 1.00E-4
**Non-Small Cell Lung Cancer**															
A549/ATCC	0.557	2.319	2.320	2.241	2.094	1.136	0.907	100	96	87	33	20	4.83E-6	> 1.00E-4	> 1.00E-4
EKVX	0.560	2.456	2.375	2.308	2.122	1.348	0.990	96	92	82	42	23	6.22E-6	> 1.00E-4	> 1.00E-4
HOP-62	0.812	1.984	1.933	1.836	1.820	1.232	0.894	96	87	86	36	7	5.21E-6	> 1.00E-4	> 1.00E-4
HOP-92	1.053	1.528	1.481	1.464	1.408	1.115	0.816	90	86	75	13	-23	2.52E-6	2.33E-5	> 1.00E-4
NCI-H226	0.865	2.444	2.353	2.274	2.186	1.744	1.230	94	89	84	56	23	1.49E-5	> 1.00E-4	> 1.00E-4
NCI-H23	0.580	2.366	2.245	2.130	1.999	1.182	0.677	93	87	79	34	5	4.40E-6	> 1.00E-4	> 1.00E-4
NCI-H322M	0.659	2.052	1.977	1.849	1.926	1.459	0.814	95	85	91	57	11	1.44E-5	> 1.00E-4	> 1.00E-4
NCI-H460	0.246	2.713	2.745	2.748	2.682	0.761	0.354	101	101	99	21	4	4.23E-6	> 1.00E-4	> 1.00E-4
NCI-H522	0.900	1.965	1.821	1.749	1.702	0.764	0.442	86	80	75	-15	-51	1.90E-6	6.80E-6	9.44E-5
**Colon Cancer**															
COLO 205	0.405	1.729	1.769	1.645	1.663	1.016	0.404	103	94	95	46	.	8.35E-6	9.82E-5	> 1.00E-4
HCC-2998	0.560	2.350	2.354	2.263	2.301	1.619	1.050	100	95	97	59	27	1.94E-5	> 1.00E-4	> 1.00E-4
HCT-116	0.281	2.540	2.301	2.287	2.139	0.366	0.249	89	89	82	4	-11	2.57E-6	1.77E-5	> 1.00E-4
HCT-15	0.234	1.994	1.940	1.837	1.560	0.558	0.337	97	91	75	18	6	2.78E-6	> 1.00E-4	> 1.00E-4
HT29	0.203	1.314	1.214	1.236	1.148	0.213	0.152	91	93	85	1	-25	2.61E-6	1.08E-5	> 1.00E-4
KM12	0.426	2.502	2.522	2.423	2.366	1.288	0.474	101	96	93	41	2	6.86E-6	> 1.00E-4	> 1.00E-4
SW-620	0.269	2.109	2.065	1.960	1.941	0.507	0.311	98	92	91	13	2	3.35E-6	> 1.00E-4	> 1.00E-4
**CNS Cancer**															
SF-268	0.469	1.905	1.877	1.782	1.788	1.113	0.723	98	91	92	45	18	7.77E-6	> 1.00E-4	> 1.00E-4
SF-295	0.632	2.292	2.241	2.177	2.064	0.783	0.459	97	93	86	9	-27	2.95E-6	1.77E-5	> 1.00E-4
SF-539	0.835	2.531	2.554	2.362	2.342	0.815	0.377	101	90	89	-2	-55	2.66E-6	9.40E-6	8.06E-5
SNB-19	0.604	2.047	2.002	1.869	1.842	1.001	0.598	97	88	86	27	-1	4.11E-6	9.17E-5	> 1.00E-4
SNB-75	0.719	1.396	1.269	1.237	1.248	0.943	0.596	81	77	78	33	-17	4.21E-6	4.55E-5	> 1.00E-4
U251	0.364	1.751	1.814	1.649	1.768	0.546	0.235	105	93	101	13	-35	3.81E-6	1.86E-5	> 1.00E-4
**Melanoma**															
LOX IMVI	0.363	2.708	2.553	2.424	2.390	0.657	0.310	93	88	86	13	-15	3.11E-6	2.90E-5	> 1.00E-4
MALME-3M	0.779	1.492	1.436	1.368	1.402	1.065	0.849	92	83	87	40	10	6.16E-6	> 1.00E-4	> 1.00E-4
M14	0.513	2.125	2.025	1.952	1.928	0.690	0.434	94	89	88	11	-15	3.10E-6	2.60E-5	> 1.00E-4
MDA-MB-435	0.432	2.331	2.274	2.167	1.356	0.289	0.199	97	91	49	-33	-54	9.29E-7	3.93E-6	6.46E-5
SK-MEL-2	0.988	2.330	2.189	2.061	2.133	1.107	0.750	89	80	85	9	-24	2.90E-6	1.86E-5	> 1.00E-4
SK-MEL-28	0.567	1.798	1.791	1.724	1.712	0.951	0.704	99	94	93	31	11	4.97E-6	> 1.00E-4	> 1.00E-4
SK-MEL-5	0.529	2.912	2.884	2.723	2.323	0.535	0.269	99	92	75	.	-49	2.17E-6	1.01E-5	> 1.00E-4
UACC-257	0.903	1.943	1.924	1.796	1.843	1.434	1.099	98	86	90	51	19	1.08E-5	> 1.00E-4	> 1.00E-4
UACC-62	0.961	2.937	2.838	2.677	2.554	0.958	0.876	95	87	81	.	-9	2.39E-6	9.91E-6	> 1.00E-4
**Ovarian Cancer**															
IGROV1	0.737	2.317	2.352	2.237	2.210	1.120	0.886	102	95	93	24	9	4.23E-6	> 1.00E-4	> 1.00E-4
OVCAR-3	0.492	1.606	1.634	1.566	1.571	0.825	0.477	102	96	97	30	-3	5.01E-6	8.08E-5	> 1.00E-4
OVCAR-4	0.604	1.481	1.503	1.448	1.392	1.027	0.786	102	96	90	48	21	9.08E-6	> 1.00E-4	> 1.00E-4
OVCAR-5	0.567	1.556	1.516	1.464	1.457	0.885	0.500	96	91	90	32	-12	4.90E-6	5.38E-5	> 1.00E-4
OVCAR-8	0.454	1.993	1.976	1.895	1.887	1.184	0.487	99	94	93	47	2	8.78E-6	> 1.00E-4	> 1.00E-4
NCI/ADR-RES	0.357	1.667	1.612	1.548	1.456	0.644	0.438	96	91	84	22	6	3.52E-6	> 1.00E-4	> 1.00E-4
SK-OV-3	0.962	2.213	2.190	2.109	2.182	1.547	0.948	98	92	98	47	-1	8.62E-6	9.33E-5	> 1.00E-4
**Renal Cancer**															
786-0	0.670	2.362	2.245	2.233	2.163	1.298	0.798	93	92	88	37	8	5.59E-6	> 1.00E-4	> 1.00E-4
A498	1.356	2.016	1.754	1.718	1.635	1.332	0.942	60	55	42	-2	-31	2.40E-7	9.10E-6	> 1.00E-4
ACHN	0.336	1.472	1.521	1.414	1.391	0.958	0.581	104	95	93	55	22	1.39E-5	> 1.00E-4	> 1.00E-4
CAKI-1	0.325	2.155	2.135	1.952	1.991	1.287	0.480	99	89	91	53	8	1.14E-5	> 1.00E-4	> 1.00E-4
RXF 393	0.568	1.356	1.313	1.238	1.188	0.936	0.536	95	85	79	47	-6	7.90E-6	7.81E-5	> 1.00E-4
SN12C	0.531	2.103	2.009	1.958	1.934	1.101	0.628	94	91	89	36	6	5.50E-6	> 1.00E-4	> 1.00E-4
TK-10	0.856	2.673	2.609	2.573	2.608	1.144	0.686	96	94	96	16	-20	3.77E-6	2.78E-5	> 1.00E-4
UO-31	0.686	2.380	2.215	2.007	2.006	1.310	0.947	90	78	78	37	15	4.78E-6	> 1.00E-4	> 1.00E-4
**Prostate Cancer**															
PC-3	0.732	2.688	2.609	2.441	2.257	1.359	0.880	96	87	78	32	8	4.07E-6	> 1.00E-4	> 1.00E-4
DU-145	0.332	1.733	1.786	1.680	1.622	1.150	0.487	104	96	92	58	11	1.50E-5	> 1.00E-4	> 1.00E-4
**Breast Cancer**															
MCF7	0.406	2.684	2.453	2.383	2.299	0.893	0.700	90	87	83	21	13	3.44E-6	> 1.00E-4	> 1.00E-4
MDA-MB-231/ATCC	0.541	1.490	1.467	1.395	1.351	0.751	0.473	98	90	85	22	-13	3.62E-6	4.34E-5	> 1.00E-4
HS 578T	0.766	1.821	1.755	1.645	1.651	1.024	0.677	94	83	84	24	-12	3.71E-6	4.75E-5	> 1.00E-4
BT-549	0.905	1.857	1.833	1.712	1.674	1.041	0.801	97	85	81	14	-12	2.90E-6	3.57E-5	> 1.00E-4
T-47D	0.504	1.153	1.110	1.059	1.036	0.654	0.493	93	85	82	23	-2	3.48E-6	8.13E-5	> 1.00E-4
MDA-MB-468	0.652	1.619	1.498	1.451	1.422	0.798	0.526	87	83	80	15	-19	2.88E-6	2.75E-5	> 1.00E-4

National Cancer Institute Developmental Therapeutics Program Mean Graphs	NSC : D - 785543/1	Units :Molar	SSPL :0JPC	EXP. ID :1508NS40
	Report Date :September 22, 2015		Test Date :August 17, 2015	

Panel/Cell Line	Log_{10}GI50	Log_{10}TGI	Log_{10}LC50
**Leukemia**			
CCRF-CEM	-5.40	> -4.00	> -4.00
HL-60(TB)	-5.63	-5.07	> -4.00
K-562	-5.74	> -4.00	> -4.00
MOLT-4	-5.49	> -4.00	> -4.00
RPMI-8226	-5.58	> -4.00	> -4.00
**Non-Small Cell Lung Cancer**			
A549/ATCC	-5.32	> -4.00	> -4.00
EKVX	-5.21	> -4.00	> -4.00
HOP-62	-5.28	> -4.00	> -4.00
HOP-92	-5.60	-4.63	> -4.00
NCI-H226	-4.83	> -4.00	> -4.00
NCI-H23	-5.36	> -4.00	> -4.00
NCI-H322M	-4.84	> -4.00	> -4.00
NCI-H460	-5.37	> -4.00	> -4.00
NCI-H522	-5.72	-5.17	-4.02
**Colon Cancer**			
COLO 205	-5.08	-4.01	> -4.00
HCC-2998	-4.71	> -4.00	> -4.00
HCT-116	-5.59	-4.75	> -4.00
HCT-15	-5.56	> -4.00	> -4.00
HT29	-5.58	-4.97	> -4.00
KM12	-5.16	> -4.00	> -4.00
SW-620	-5.48	> -4.00	> -4.00
**CNS Cancer**			
SF-268	-5.11	> -4.00	> -4.00
SF-295	-5.53	-4.75	> -4.00
SF-539	-5.57	-5.03	-4.09
SNB-19	-5.39	-4.04	> -4.00
SNB-75	-5.38	-4.34	> -4.00
U251	-5.42	-4.73	> -4.00
**Melanoma**			
LOX IMVI	-5.51	-4.54	> -4.00
MALME-3M	-5.21	> -4.00	> -4.00
M14	-5.51	-4.58	> -4.00
MDA-MB-435	-6.03	-5.41	-4.19
SK-MEL-2	-5.54	-4.73	> -4.00
SK-MEL-28	-5.30	> -4.00	> -4.00
SK-MEL-5	-5.66	-4.99	> -4.00
UACC-257	-4.97	> -4.00	> -4.00
UACC-62	-5.62	-5.00	> -4.00
**Ovarian Cancer**			
IGROV1	-5.37	> -4.00	> -4.00
OVCAR-3	-5.30	-4.09	> -4.00
OVCAR-4	-5.04	> -4.00	> -4.00
OVCAR-5	-5.31	-4.27	> -4.00
OVCAR-8	-5.06	> -4.00	> -4.00
NCI/ADR-RES	-5.45	> -4.00	> -4.00
SK-OV-3	-5.06	-4.03	> -4.00
**Renal Cancer**			
786-0	-5.25	> -4.00	> -4.00
A498	-6.62	-5.04	> -4.00
ACHN	-4.86	> -4.00	> -4.00
CAKI-1	-4.94	> -4.00	> -4.00
RXF 393	-5.10	-4.11	> -4.00
SN12C	-5.26	> -4.00	> -4.00
TK-10	-5.42	-4.56	> -4.00
UO-31	-5.32	> -4.00	> -4.00
**Prostate Cancer**			
PC-3	-5.39	> -4.00	> -4.00
DU-145	-4.82	> -4.00	> -4.00
**Breast Cancer**			
MCF7	-5.46	> -4.00	> -4.00
MDA-MB-231/ATCC	-5.44	-4.36	> -4.00
HS 578T	-5.43	-4.32	> -4.00
BT-549	-5.54	-4.45	> -4.00
T-47D	-5.46	-4.09	> -4.00
MDA-MB-468	-5.54	-4.56	> -4.00
_MID	-5.37	-4.28	-4.01
Delta	1.25	1.13	0.18
Range	1.91	1.41	0.19

GI50 / TGI / LC50 axes: +3 +2 +1 0 -1 -2 -3

**National Cancer Institute Developmental Therapeutics Program** **Dose Response Curves**	NSC : D - 785543/1	SSPL:0JPC	EXP. ID:1508NS40
	Report Date:September 22, 2015	Test Date:August 17, 2015	

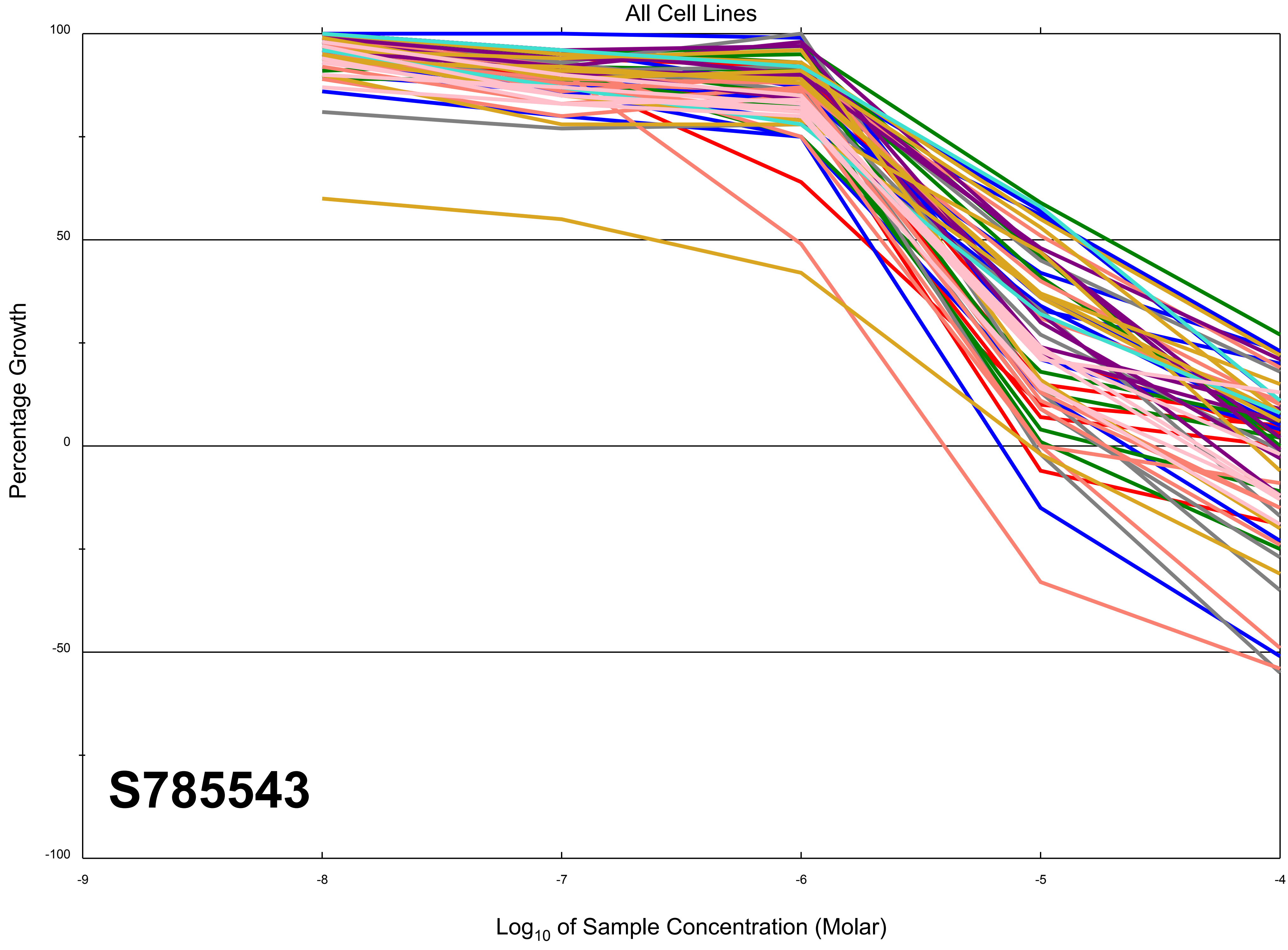

Supplement: Supplementary file 1 [file ijms-23-14374-s001.zip › S14 .zip!/Compound 3e -AllReports5DOSE.pdf]
