# Supplementary material for: Antiproliferative Activity Predictor: A New Reliable In Silico Tool for Drug Response Prediction against NCI60 Panel
Source: Int J Mol Sci. 2022 Nov 19;23(22):14374. doi: 10.3390/ijms232214374 (PMC9694168; doi:10.3390/ijms232214374)

National Cancer Institute Developmental Therapeutics Program
Dose Response Curves

NSC: D - 785541 / 1		SSPL: 0JPC	EXP. ID: 1508NS40
Report Date: September 22, 2015		Test Date: August 17, 2015	

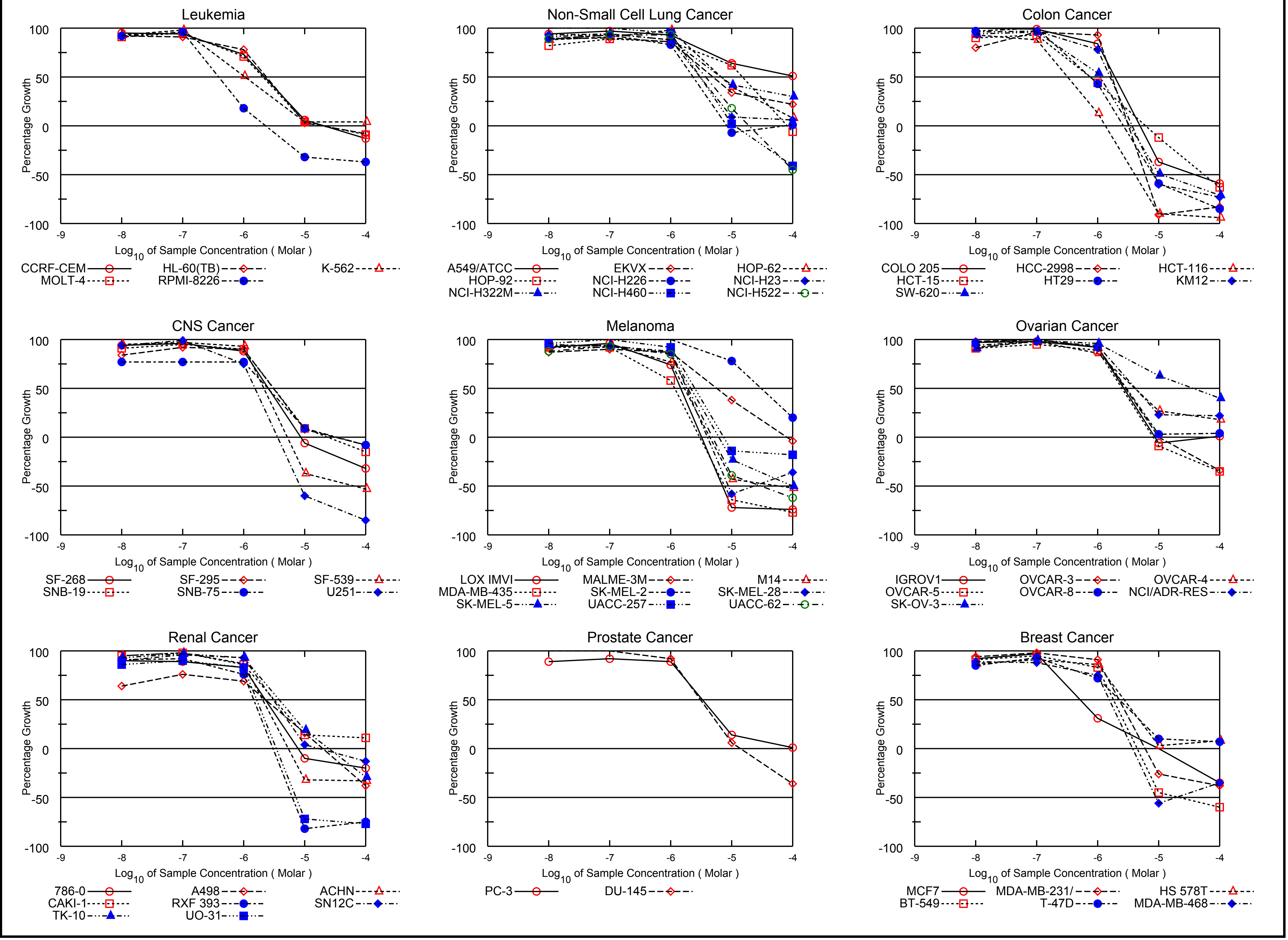

National Cancer Institute Developmental Therapeutics Program
In-Vitro Testing Results

NSC : D - 785541 / 1	Experiment ID : 1508NS40	Test Type : 08	Units : Molar
Report Date : September 22, 2015	Test Date : August 17, 2015	QNS :	MC :
COMI : NV1994	Stain Reagent : SRB Dual-Pass Related	SSPL : 0JPC	

			Log10 Concentration													
	Time		Mean Optical Densities					Percent Growth							
Panel/Cell Line	Zero	Ctrl	-8.0	-7.0	-6.0	-5.0	-4.0	-8.0	-7.0	-6.0	-5.0	-4.0	GI50	TGI	LC50
**Leukemia**															
CCRF-CEM	0.775	3.093	2.968	2.959	2.477	0.909	0.671	95	94	73	6	-13	2.22E-6	2.00E-5	> 1.00E-4
HL-60(TB)	0.866	3.244	3.051	3.037	2.729	0.926	0.793	92	91	78	3	-8	2.36E-6	1.69E-5	> 1.00E-4
K-562	0.299	2.258	2.120	2.220	1.306	0.383	0.370	93	98	51	4	4	1.07E-6	> 1.00E-4	> 1.00E-4
MOLT-4	0.563	2.584	2.409	2.492	2.001	0.638	0.511	91	95	71	4	-9	2.06E-6	1.93E-5	> 1.00E-4
RPMI-8226	0.922	2.859	2.700	2.774	1.278	0.630	0.578	92	96	18	-32	-37	3.89E-7	2.33E-6	> 1.00E-4
**Non-Small Cell Lung Cancer**															
A549/ATCC	0.557	2.319	2.212	2.271	2.199	1.687	1.461	94	97	93	64	51	> 1.00E-4	> 1.00E-4	> 1.00E-4
EKVX	0.560	2.456	2.251	2.275	2.195	1.212	0.986	89	90	86	34	22	5.00E-6	> 1.00E-4	> 1.00E-4
HOP-62	0.812	1.984	1.841	1.877	1.959	1.285	0.903	88	91	98	40	8	6.80E-6	> 1.00E-4	> 1.00E-4
HOP-92	1.053	1.528	1.444	1.475	1.460	1.348	0.995	82	89	86	62	-6	1.51E-5	8.28E-5	> 1.00E-4
NCI-H226	0.865	2.444	2.276	2.330	2.183	0.801	0.885	89	93	83	-7	1	2.33E-6	.	> 1.00E-4
NCI-H23	0.580	2.366	2.235	2.260	2.144	0.734	0.686	93	94	88	9	6	2.99E-6	> 1.00E-4	> 1.00E-4
NCI-H322M	0.659	2.052	1.928	1.971	1.886	1.244	1.076	91	94	88	42	30	6.69E-6	> 1.00E-4	> 1.00E-4
NCI-H460	0.246	2.713	2.714	2.731	2.594	0.300	0.144	100	101	95	2	-41	3.06E-6	1.12E-5	> 1.00E-4
NCI-H522	0.900	1.965	1.845	1.889	1.894	1.090	0.497	89	93	93	18	-45	3.75E-6	1.93E-5	> 1.00E-4
**Colon Cancer**															
COLO 205	0.405	1.729	1.731	1.715	1.513	0.256	0.167	100	99	84	-37	-59	1.90E-6	4.95E-6	3.96E-5
HCC-2998	0.560	2.350	1.995	2.279	2.228	0.051	0.095	80	96	93	-91	-83	1.72E-6	3.21E-6	6.00E-6
HCT-116	0.281	2.540	2.382	2.267	0.565	0.030	0.018	93	88	13	-90	-94	3.19E-7	1.33E-6	4.10E-6
HCT-15	0.234	1.994	1.812	1.870	1.017	0.207	0.088	90	93	44	-12	-63	7.69E-7	6.18E-6	5.65E-5
HT29	0.203	1.314	1.285	1.373	0.677	0.083	0.031	97	105	43	-59	-85	7.63E-7	2.62E-6	8.14E-6
KM12	0.426	2.502	2.416	2.449	2.050	0.170	0.117	96	97	78	-60	-73	1.60E-6	3.67E-6	8.44E-6
SW-620	0.269	2.109	1.991	2.036	1.254	0.136	0.078	94	96	54	-49	-71	1.08E-6	3.31E-6	1.06E-5
**CNS Cancer**															
SF-268	0.469	1.905	1.822	1.842	1.734	0.440	0.317	94	96	88	-6	-32	2.53E-6	8.58E-6	> 1.00E-4
SF-295	0.632	2.292	2.032	2.157	2.118	0.763	0.581	84	92	90	8	-8	3.05E-6	3.12E-5	> 1.00E-4
SF-539	0.835	2.531	2.444	2.474	2.409	0.523	0.389	95	97	93	-37	-53	2.13E-6	5.16E-6	6.09E-5
SNB-19	0.604	2.047	1.922	1.971	1.904	0.730	0.514	91	95	90	9	-15	3.11E-6	2.33E-5	> 1.00E-4
SNB-75	0.719	1.396	1.239	1.242	1.241	0.777	0.665	77	77	77	9	-8	2.49E-6	3.39E-5	> 1.00E-4
U251	0.364	1.751	1.673	1.732	1.405	0.146	0.056	94	99	75	-60	-85	1.53E-6	3.60E-6	8.45E-6
**Melanoma**															
LOX IMVI	0.363	2.708	2.528	2.610	2.099	0.101	0.095	92	96	74	-72	-74	1.46E-6	3.21E-6	7.04E-6
MALME-3M	0.779	1.492	1.398	1.423	1.400	1.053	0.745	87	90	87	38	-4	5.77E-6	7.88E-5	> 1.00E-4
M14	0.513	2.125	2.019	2.029	1.762	0.293	0.245	93	94	77	-43	-52	1.69E-6	4.40E-6	5.73E-5
MDA-MB-435	0.432	2.331	2.179	2.186	1.527	0.157	0.099	92	92	58	-64	-77	1.16E-6	2.98E-6	7.70E-6
SK-MEL-2	0.988	2.330	2.359	2.422	2.340	2.035	1.261	102	107	101	78	20	3.06E-5	> 1.00E-4	> 1.00E-4
SK-MEL-28	0.567	1.798	1.733	1.714	1.654	0.240	0.362	95	93	88	-58	-36	1.83E-6	4.02E-6	.
SK-MEL-5	0.529	2.912	2.771	2.763	2.575	0.408	0.265	94	94	86	-23	-50	2.14E-6	6.15E-6	> 1.00E-4
UACC-257	0.903	1.943	1.897	1.989	1.858	0.775	0.739	96	104	92	-14	-18	2.48E-6	7.34E-6	> 1.00E-4
UACC-62	0.961	2.937	2.697	2.791	2.644	0.589	0.363	88	93	85	-39	-62	1.92E-6	4.87E-6	3.01E-5
**Ovarian Cancer**															
IGROV1	0.737	2.317	2.274	2.292	2.192	0.691	0.750	97	98	92	-6	1	2.68E-6	.	> 1.00E-4
OVCAR-3	0.492	1.606	1.583	1.616	1.515	0.490	0.323	98	101	92	.	-34	2.84E-6	9.90E-6	> 1.00E-4
OVCAR-4	0.604	1.481	1.407	1.460	1.358	0.841	0.762	92	98	86	27	18	4.07E-6	> 1.00E-4	> 1.00E-4
OVCAR-5	0.567	1.556	1.470	1.505	1.447	0.516	0.371	91	95	89	-9	-35	2.50E-6	8.08E-6	> 1.00E-4
OVCAR-8	0.454	1.993	1.942	2.007	1.874	0.499	0.518	97	101	92	3	4	2.97E-6	> 1.00E-4	> 1.00E-4
NCI/ADR-RES	0.357	1.667	1.593	1.647	1.587	0.665	0.651	94	98	94	23	22	4.20E-6	> 1.00E-4	> 1.00E-4
SK-OV-3	0.962	2.213	2.102	2.195	2.168	1.750	1.460	91	99	96	63	40	3.64E-5	> 1.00E-4	> 1.00E-4
**Renal Cancer**															
786-0	0.670	2.362	2.195	2.182	2.079	0.606	0.536	90	89	83	-10	-20	2.28E-6	7.88E-6	> 1.00E-4
A498	1.356	2.016	1.780	1.860	1.809	1.463	0.841	64	76	69	16	-38	2.26E-6	1.99E-5	> 1.00E-4
ACHN	0.336	1.472	1.420	1.446	1.327	0.229	0.225	95	98	87	-32	-33	2.05E-6	5.40E-6	> 1.00E-4
CAKI-1	0.325	2.155	2.062	2.113	1.902	0.580	0.528	95	98	86	14	11	3.17E-6	> 1.00E-4	> 1.00E-4
RXF 393	0.568	1.356	1.287	1.295	1.168	0.104	0.144	91	92	76	-82	-75	1.46E-6	3.04E-6	6.30E-6
SN12C	0.531	2.103	1.953	2.040	1.998	0.597	0.464	90	96	93	4	-13	3.06E-6	1.77E-5	> 1.00E-4
TK-10	0.856	2.673	2.547	2.625	2.549	1.206	0.607	93	97	93	19	-29	3.83E-6	2.50E-5	> 1.00E-4
UO-31	0.686	2.380	2.140	2.205	2.088	0.189	0.155	86	90	83	-72	-77	1.63E-6	3.41E-6	7.17E-6
**Prostate Cancer**															
PC-3	0.732	2.688	2.482	2.540	2.473	1.001	0.759	89	92	89	14	1	3.30E-6	> 1.00E-4	> 1.00E-4
DU-145	0.332	1.733	1.743	1.743	1.617	0.422	0.213	101	101	92	6	-36	3.08E-6	1.42E-5	> 1.00E-4
**Breast Cancer**															
MCF7	0.406	2.684	2.509	2.620	1.112	0.403	0.264	92	97	31	.	-35	5.16E-7	9.48E-6	> 1.00E-4
MDA-MB-231/ATCC	0.541	1.490	1.437	1.475	1.409	0.400	0.336	94	98	91	-26	-38	2.25E-6	6.00E-6	> 1.00E-4
HS 578T	0.766	1.821	1.685	1.727	1.672	0.797	0.855	87	91	86	3	8	2.71E-6	> 1.00E-4	> 1.00E-4
BT-549	0.905	1.857	1.772	1.814	1.693	0.499	0.363	91	95	83	-45	-60	1.81E-6	4.45E-6	2.18E-5
T-47D	0.504	1.153	1.054	1.108	0.972	0.568	0.551	85	93	72	10	7	2.26E-6	> 1.00E-4	> 1.00E-4
MDA-MB-468	0.652	1.619	1.511	1.507	1.375	0.287	0.425	89	88	75	-56	-35	1.55E-6	3.73E-6	.

National Cancer Institute Developmental Therapeutics Program Mean Graphs	NSC : D - 785541/1	Units :Molar	SSPL :0JPC	EXP. ID :1508NS40
	Report Date :September 22, 2015		Test Date :August 17, 2015	

Panel/Cell Line	Log_{10}GI50	Log_{10}TGI	Log_{10}LC50
**Leukemia**			
CCRF-CEM	-5.65	-4.70	> -4.00
HL-60(TB)	-5.63	-4.77	> -4.00
K-562	-5.97	> -4.00	> -4.00
MOLT-4	-5.69	-4.72	> -4.00
RPMI-8226	-6.41	-5.63	> -4.00
**Non-Small Cell Lung Cancer**			
A549/ATCC	> -4.00	> -4.00	> -4.00
EKVX	-5.30	> -4.00	> -4.00
HOP-62	-5.17	> -4.00	> -4.00
HOP-92	-4.82	-4.08	> -4.00
NCI-H226	-5.63		> -4.00
NCI-H23	-5.52	> -4.00	> -4.00
NCI-H322M	-5.17	> -4.00	> -4.00
NCI-H460	-5.51	-4.95	> -4.00
NCI-H522	-5.43	-4.72	> -4.00
**Colon Cancer**			
COLO 205	-5.72	-5.31	-4.40
HCC-2998	-5.77	-5.49	-5.22
HCT-116	-6.50	-5.88	-5.39
HCT-15	-6.11	-5.21	-4.25
HT29	-6.12	-5.58	-5.09
KM12	-5.80	-5.43	-5.07
SW-620	-5.97	-5.48	-4.97
**CNS Cancer**			
SF-268	-5.60	-5.07	> -4.00
SF-295	-5.52	-4.51	> -4.00
SF-539	-5.67	-5.29	-4.22
SNB-19	-5.51	-4.63	> -4.00
SNB-75	-5.60	-4.47	> -4.00
U251	-5.81	-5.44	-5.07
**Melanoma**			
LOX IMVI	-5.84	-5.49	-5.15
MALME-3M	-5.24	-4.10	> -4.00
M14	-5.77	-5.36	-4.24
MDA-MB-435	-5.94	-5.53	-5.11
SK-MEL-2	-4.51	> -4.00	> -4.00
SK-MEL-28	-5.74	-5.40	
SK-MEL-5	-5.67	-5.21	> -4.00
UACC-257	-5.61	-5.13	> -4.00
UACC-62	-5.72	-5.31	-4.52
**Ovarian Cancer**			
IGROV1	-5.57		> -4.00
OVCAR-3	-5.55	-5.00	> -4.00
OVCAR-4	-5.39	> -4.00	> -4.00
OVCAR-5	-5.60	-5.09	> -4.00
OVCAR-8	-5.53	> -4.00	> -4.00
NCI/ADR-RES	-5.38	> -4.00	> -4.00
SK-OV-3	-4.44	> -4.00	> -4.00
**Renal Cancer**			
786-0	-5.64	-5.10	> -4.00
A498	-5.65	-4.70	> -4.00
ACHN	-5.69	-5.27	> -4.00
CAKI-1	-5.50	> -4.00	> -4.00
RXF 393	-5.83	-5.52	-5.20
SN12C	-5.51	-4.75	> -4.00
TK-10	-5.42	-4.60	> -4.00
UO-31	-5.79	-5.47	-5.14
**Prostate Cancer**			
PC-3	-5.48	> -4.00	> -4.00
DU-145	-5.51	-4.85	> -4.00
**Breast Cancer**			
MCF7	-6.29	-5.02	> -4.00
MDA-MB-231/ATCC	-5.65	-5.22	> -4.00
HS 578T	-5.57	> -4.00	> -4.00
BT-549	-5.74	-5.35	-4.66
T-47D	-5.65	> -4.00	> -4.00
MDA-MB-468	-5.81	-5.43	
_MID	-5.59	-4.81	-4.24
Delta	0.91	1.07	1.15
Range	2.5	1.88	1.39

+3 +2 +1 0 -1 -2 -3

National Cancer Institute Developmental Therapeutics Program Dose Response Curves	NSC : D - 785541/1	SSPL:0JPC	EXP. ID:1508NS40
	Report Date:September 22, 2015	Test Date:August 17, 2015	

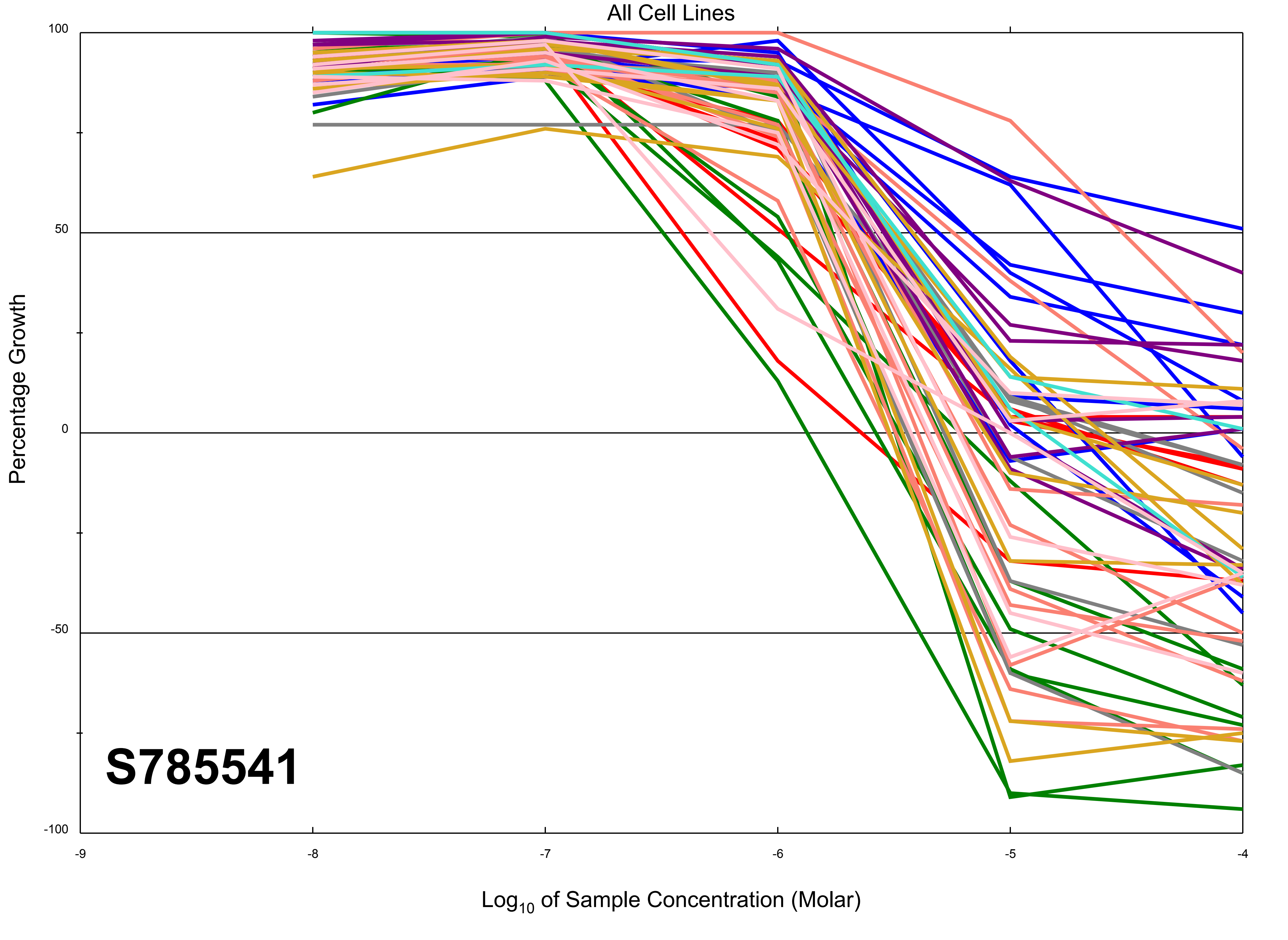

Supplement: Supplementary file 1 [file ijms-23-14374-s001.zip › S14 .zip!/Compound 1a -AllReports-5DOSE.pdf]
